# Quantification of full and empty particles of adeno-associated virus vectors via a novel dual fluorescence-linked immunosorbent assay

**DOI:** 10.1016/j.omtm.2024.101291

**Published:** 2024-06-24

**Authors:** Sereirath Soth, Mikako Takakura, Masahiro Suekawa, Takayuki Onishi, Kiichi Hirohata, Tamami Hashimoto, Takahiro Maruno, Mitsuko Fukuhara, Yasuo Tsunaka, Tetsuo Torisu, Susumu Uchiyama

**Affiliations:** 1 Department of Biotechnology, Graduate School of Engineering, Osaka University, 2-1 Yamadaoka, Suita, Osaka 565-0871, Japan; 2 Exploratory Research Center on Life and Living Systems, National Institutes of Natural Sciences, 5-1 Higashiyama, Myodaiji, Okazaki, Aichi 444-8787, Japan

**Keywords:** adeno-associated virus, analytical ultracentrifugation, digital polymerase chain reaction, enzyme-linked immunosorbent assay, dual fluorescence-linked immunosorbent assay, mass photometry

## Abstract

The adeno-associated virus (AAV) vector is one of the most advanced platforms for gene therapy because of its low immunogenicity and non-pathogenicity. The concentrations of both AAV vector empty particles, which do not contain DNA and do not show any efficacy, and AAV vector full particles (FPs), which contain DNA, are important quality attributes. In this study, a dual fluorescence-linked immunosorbent assay (dFLISA), which uses two fluorescent dyes to quantify capsid and genome titers in a single analysis, was established. In dFLISA, capture of AAV particles, detection of capsid proteins, and release and detection of the viral genome are performed in the same well. We demonstrated that the capsid and genomic titers determined by dFLISA were comparable with those of analytical ultracentrifugation. The FP ratios determined by dFLISA were in good agreement with the expected values. In addition, we showed that dFLISA can quantify the genomic and capsid titers of crude samples. dFLISA can be easily modified for measuring other AAV vector serotypes and AAV vectors with different genome lengths. These features make dFLISA a valuable tool for the future development of AAV-based gene therapies.

## Graphical abstract

**Figure:**
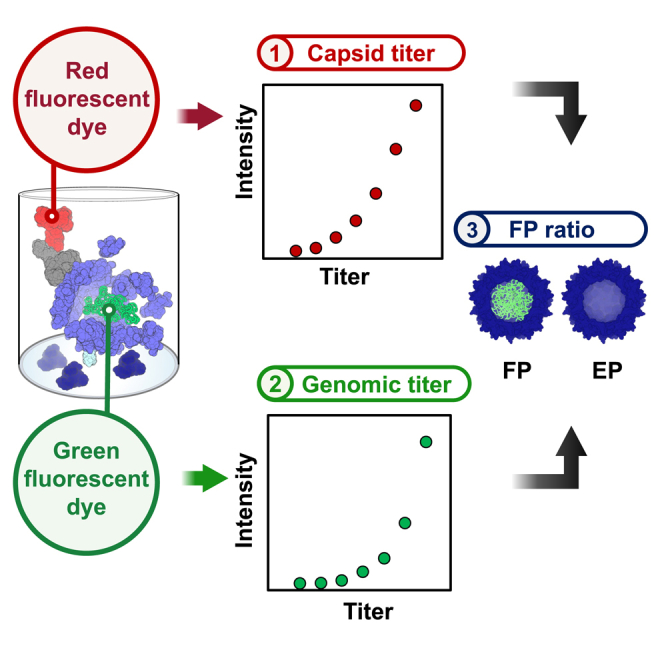

## Teaser

Uchiyama and colleagues developed dual fluorescence-linked immunosorbent assay (dFLISA), which can quantify capsid and genome titers of AAV vector in single assay. dFLISA can be applied for various serotypes of AAVs, AAVs containing different lengths of DNA, regardless of purification level.

## Introduction

Recombinant adeno-associated virus (rAAV) vectors have become highly effective tools in human gene therapy, primarily because of the exceptional properties of AAV^1^^,^^2^; its non-pathogenicity, ability to infect multiple cell types, and maintenance of the viral genome within host cells have enabled clinical successes in the treatment of genetic and acquired diseases.^3^^,^^4^^,^^5^^,^^6^^,^^7^ Another advantage of AAV vectors is that there are several serotypes, each with different tissue tropisms.^8^^,^^9^^,^^10^ Despite the advantages of using AAVs for therapeutic purposes, there are several challenges to be overcome. Empty AAV vector particles (EPs), partial particles (PPs), which lack therapeutic deoxyribonucleic acid (DNA) or only contain fragments of the genome,^11^^,^^12^ and extra filled particles (ExPs), which contains higher numbers of DNA, are generated in upstream production processes, and complete removal of the particles in downstream processes is impractical because of their physicochemical similarity to full particles (FPs), which contain therapeutic DNA.^13^^,^^14^^,^^15^^,^^16^ EPs and PPs are considered impurities that potentially trigger adverse immunogenic reactions.^11^^,^^17^^,^^18^^,^^19^ Furthermore, these impurities may compete with FPs for binding to target cell receptors, potentially reducing their therapeutic efficacy.^20^^,^^21^^,^^22^ The clinical impact of EPs is not fully understood, but they are recognized to be significant obstacles affecting FPs biodistribution and potentially provoking immune responses. Therefore, analytical techniques to evaluate the purity of AAVs are crucial for AAV vector development.^22^ Some analytical methods are available to assess the contents of FPs and EPs. Combination of genome quantitation by quantitative polymerase chain reaction (qPCR) and capsid quantitation by enzyme-linked immunosorbent assay (ELISA) has been conventionally employed for the estimation of FP ratio. Now, band sedimentation analytical ultracentrifugation (BS-AUC) is recognized as the gold standard for the size distribution analysis of AAV vectors and can quantify FPs, EPs, PPs, and ExPs with high precision.^23^^,^^24^^,^^25^ Charge detection mass spectrometry and transmission electron microscopy could be orthogonal methods for the size distribution analysis. They are able to quantify FP ratio^26^^,^^27^^,^^28^^,^^29^^,^^30^; and furthermore could provide aggregation, fragmentation, and mass distribution of packaged DNA.^31^^,^^32^^,^^33^ Mass photometry (MP) is a method that measures the mass of individual particles and provides the percentage of each kind of particle against total counts (% counts).^34^^,^^35^ However, these analytical methods have limitations, especially in the case of crude samples. For example, it is burdensome that prior purification is required before using these analyses. A combination of ELISA and qPCR,^36^^,^^37^^,^^38^ which do not require purification before analysis, has been used to quantify capsid and genome titers, respectively, and to calculate FP ratios. Besides qPCR, digital PCR (dPCR), or digital droplet PCR are used for detecting the genomic titer of the viral vector.^39^^,^^40^^,^^41^^,^^42^^,^^43^

However, the combination of ELISA and PCR is subject to inherent drawbacks of error and variability^44^^,^^45^^,^^46^^,^^47^^,^^48^ because it relies on data from two independent quantitative analyses, which are based on different principles, and capsid and genomic titers must be quantified separately using non-identical samples.^49^^,^^50^^,^^51^^,^^52^^,^^53^^,^^54^

**Figure 1:**
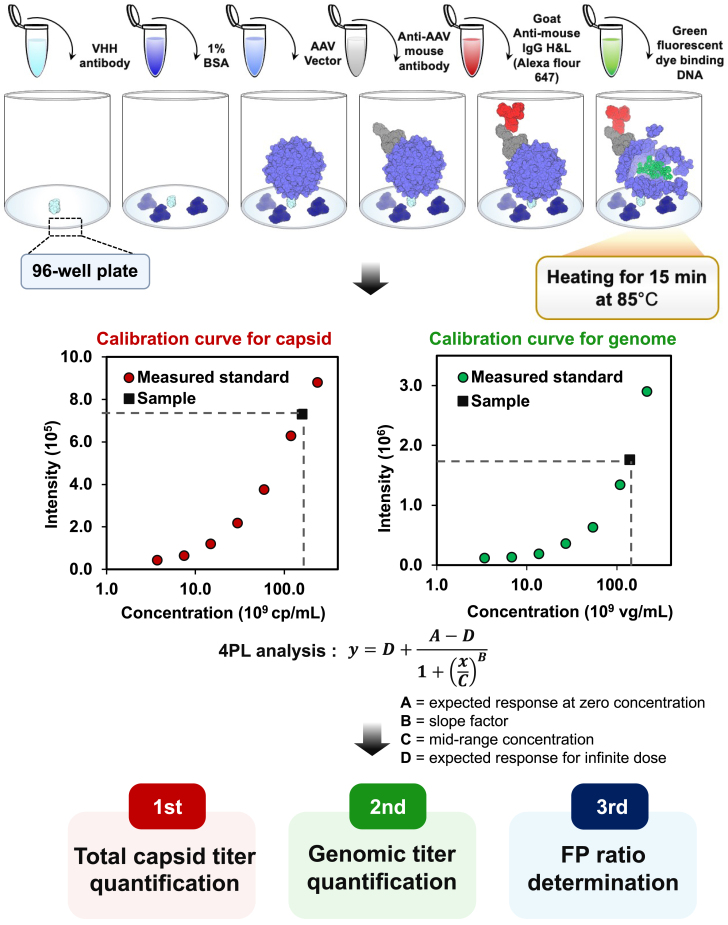
Schematic illustration of dFLISA analysis The soluble biotinylated anti-AAVX conjugate VHH affinity ligand, which exhibits high affinity for AAVX, was immobilized directly onto a black 96-well plate and used as a capture protein. Subsequently, 1% BSA was added, and the individual wells were loaded with vector stocks comprising a variety of AAV samples. A mouse monoclonal antibody targeting intact AAV particles was used as the primary antibody against AAV. To enable detection, we used a goat anti-mouse IgG H&L-labeled secondary antibody (Alexa Fluor 647). The viral capsid was disrupted, and ssDNA was released by the addition of 1× PBS to each well, followed by incubation at 85°C for 15 min. Next, we added diluted SYBR gold solution to each well and incubated the plate at room temperature for 5 min. This technique allowed us to generate the calibration curve and thus measure both red and green fluorescence, providing an assessment of the capsid and genomic titers, as well as the FP ratio, through simultaneous dual-wavelength measurements.

In our study, we aimed to establish a dual fluorescence-linked immunosorbent assay (dFLISA) as an analytical method capable of simultaneously quantifying viral capsid and genomic titers in a single analysis (Figure 1). This method is primarily based on ELISA,^53^^,^^55^ followed by genome staining where two different fluorescent dyes are employed to quantify full and empty AAV vector particles and the FP ratio. After the addition of a secondary antibody conjugated to one fluorescent dye, the plate is subjected to heat treatment to release the genome from the capsid before the introduction of the second fluorescent dye. dFLISA allows the determination of the FP ratio in a simple way with high precision, high accuracy, and high sensitivity. The capsid and genomic titers that give FP ratio in crude samples, which contain impurities, including host cell DNA and proteins, were also successfully obtained by dFLISA.

## Results

### dFLISA

A 96-well plate was first coated with anti-AAV VHH antibody, followed by the addition of bovine serum albumin (BSA) for blocking. Standards and samples were then added to the wells. The binding efficiencies of the anti-AAV VHH antibody for AAV2 and AAV8 were estimated as >98% (Figure S7). Mouse anti-AAV antibody was added after removal of unbound AAVs. Then goat anti-mouse antibody conjugated to Alexa Fluor 647 was used to detect and quantify the AAV capsid proteins. The plate was washed to remove excess goat antibody and heated at 85°C for 15 min to disrupt the capsid and release the genome. SYBR gold solution was then added to each well to detect DNA.^56^ Because SYBR gold is fluorescent only when it is bound to DNA,^42^ genomes can be quantified even when they are no longer immobilized on the plate and without washing out the unbound SYBR gold dye. Standard curves were generated using four-parameter logistic regression to calculate the capsid and genome titers. The FP ratio was calculated from these values—capsid and genome titers are considered to represent total and FP concentrations, respectively (Figure 1).

### Quantification of capsid and genomic titers by dFLISA

**Figure 2:**
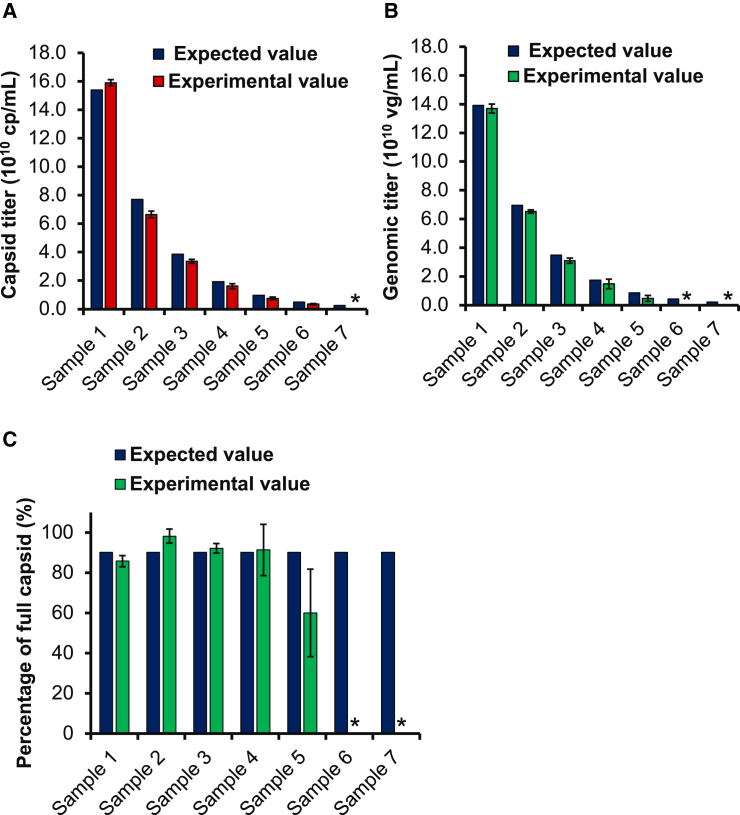
Quantification of capsid and genomic titers by dFLISA (A) Capsid titer quantification. (B) Genomic titer quantification. (C) Percentage of full capsids. In this quantitative analysis, two different samples of Serotype 8, AAV8-Lot1 and AAV8-Lot2, were used. Each sample was initially formulated in PBS, 200 mM NaCl, and 0.001% poloxamer-188. AAV8-Lot1 was used to establish the reliability of the calibration curve and had a concentration of 1.43 × 10^13^ cp/mL and 1.31 × 10^13^ vg/mL, and then diluted 60-fold with 0.05% Tween 20 in 1× PBS. AAV8-Lot2 was used as an unknown sample and had a concentration of 6.16 × 10^13^ cp/mL and 5.55 × 10^13^ vg/mL, and then diluted 400-fold with 0.05% Tween 20 in 1× PBS. Serial 2-fold dilutions were performed daily to obtain seven samples while avoiding freeze-thaw cycle and maintaining consistent operating conditions for a brief time and both samples were prepared without undergoing freeze-thaw cycles. The obtained responses were plotted using dFLISA data (experimental value) and BS-AUC data (expected value). The mean values from experiments conducted over 3 days are presented in the results. Each sample was analyzed in duplicate wells, and error bars indicate the SD within each sample. Asterisks (∗) are used to indicate cp/mL and vg/mL values that were below the limit of quantification.

The precision and accuracy of capsid and genomic titer quantification by dFLISA were evaluated by analyzing purified AAV8 samples on three separate occasions over three consecutive days (Figures 2A–2C). The capsid concentration of the original sample solution was determined in advance as 1.54 × 10^11^ capsid protein (cp)/mL by BS-AUC. These samples were serially diluted at a 1:2 ratio, resulting in the series of dilutions shown in Tables S3 and S4. For precision, the coefficient of variation (%CV) of the capsid titer was less than 15% for all samples and less than 11% for samples 1–4 (Table S3). The %CV of genomic titer quantification was less than 7% for samples 1–3, and the %CV of the genomic titer of sample 4 was 22.6%. Accuracy was evaluated based on the ratio of experimental/expected values. The ratios of samples 1–4 were consistently within the range 80%–100% for both capsid and genomic titers. The ratios of experimental/expected values of samples 5–7 were lower than 80% (Table S4).

### Determination of FP ratio via dFLISA

**Figure 3:**
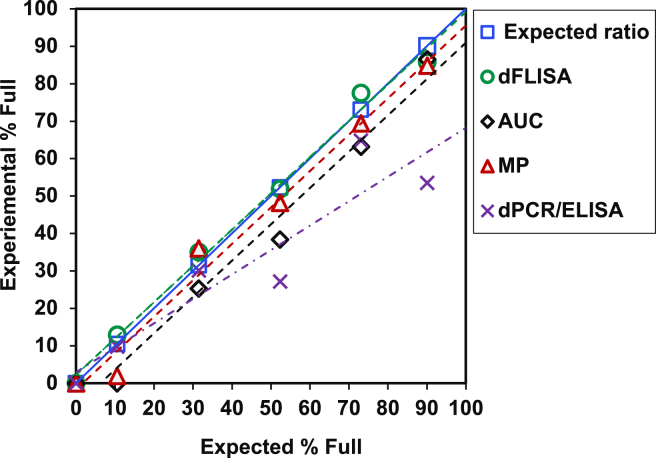
Determination of FP ratio by dFLISA and orthogonal method For the determination of the full-to-empty capsid ratio by dFLISA, capsid and genomic titer quantification was repeated on three consecutive days (days 1–3) by mixing two AAV8 samples, full and empty titers, 6.16 × 10^13^ cp/mL and 7.37 × 10^13^ cp/mL, respectively, to obtain different FP ratios ranging from 0% to 90.1% FPs. A good linear correlation was obtained between the dFLISA data (experimental % full) shown on the vertical axis and the BS-AUC data (expected % full) shown on the horizontal axis. Results are the means of 3-day experiments in which each sample was analyzed in duplicate wells, with error bars corresponding to the standard deviation (SD) of each population. As a comparison of genomic titer by orthogonal methods using mixed samples, the graph shows the relationship between the expected percentage of full capsids as determined by BS-AUC (blue square) on the vertical axis and the corresponding experimental percentage of full capsids on the horizontal axis. The graph includes data points representing experimental results obtained by dFLISA (green circle) and different orthogonal methods, specifically BS-AUC (black rhombus), MP (red triangle), and dPCR/ELISA (purple multiplication sign). The average of duplicate wells are shown, and the error bars indicate the SD within each sample.

We investigated the linearity of the FP ratio calculated by dFLISA. Samples with different FP ratios (0%, 10.5%, 31.5%, 52.3%, 73.1%, and 90.1% of FPs) were prepared by mixing two samples: AAV8-Lot2 (FP ratio 90.1%) and AAV8-Lot3 (FP ratio 0%). Excellent correlation and linearity in the FP ratio were observed, with an R^2^ value of >0.99, and the slope of the plot against the expected values was 0.97. In addition, the %CV of the FP ratio was less than 25%. These results indicate that dFLISA has sufficient precision, accuracy, and linearity for FP ratio determination (Figure 3).

According to the criteria for accuracy and precision described in the materials and methods, the concentration of samples 1–3 should be within the quantification range of dFLISA, and sample 4 (1.61 × 10^10^ cp/mL, 1.47 ×10^10^ vector genome (vg)/mL) met the criteria for the limit of quantification (LOQ). In addition, the concentration was calculated from fluorescence intensities of blank +10 SD, which is also used to determine LOQ. The capsid titer for sample 4 was higher than that of blank intensity +10 SD (Tables S3 and S5), while the genome titer for sample 4 was lower than that of blank intensity +10 SD (Tables S4 and S6). The higher values were determined as the LOQ of dFLISA for capsid and genome titer quantification: 1.61 × 10^10^ cp/mL for capsid titer and 1.70 × 10^10^ vg/mL for genomic titer.

### Comparison of genomic titer by orthogonal methods using mixed samples

We evaluated the suitability of dFLISA for vector analysis by comparing it with various particle-measuring techniques, including BS-AUC, MP, and the combined dPCR/ELISA method (Tables S7 and S8).^47^^,^^54^^,^^57^ As shown in Figure 3, dFLISA showed good correlation with the orthogonal determination of the FP ratio. The results of the MP were in good agreement with that of dFLISA, except for a 10% FP sample. BS-AUC showed lower FP values than expected, and the dPCR/ELISA results were in close agreement with the expected values of 73.1%, 31.5%, and 10.5% FP. However, the dPCR/ELISA results for the 90.1% and 52.3% FP samples were very different because of variations in the PCR results.

### Applicability of dFLISA for crude samples

**Figure 4:**
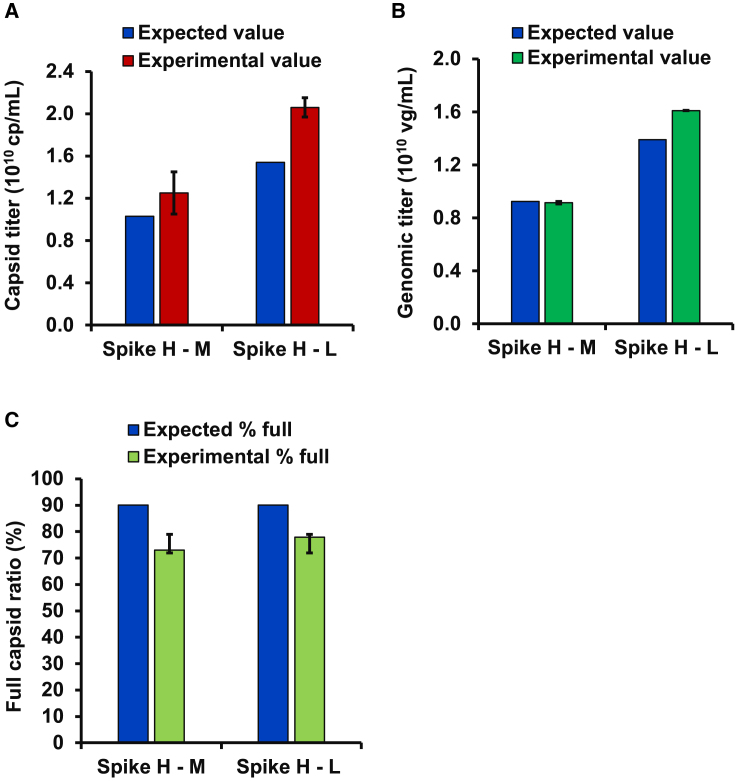
Applicability of dFLISA for analyzing crude samples (A) Capsid titer quantification. (B) Genomic titer quantification. (C) Percentage of full capsids. The various dilution factors for spike recovery were assessed by comparing the experimental values, as determined by the dFLISA method, with the expected values obtained by BS-AUC analysis. The expected values derived from BS-AUC (blue) were compared with actual values for three parameters: capsid titer (red), genomic titer (green), and FP ratio (light green). Reported results represent the average measurements from duplicate wells. H, high concentration spike; M, middle concentration spike; L, low concentration spike. All data are presented as the mean and SD (*n* = 2).

**Figure 5:**
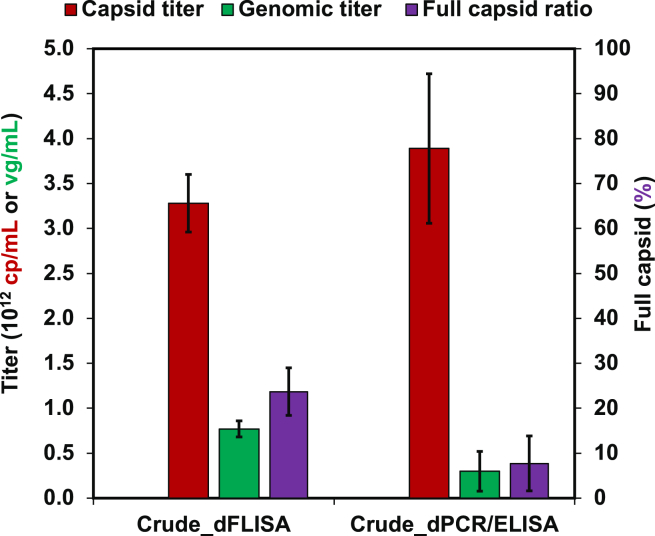
Determination of dFLISA’s ability to quantify crude samples AAV8 content in crude samples was analyzed by three methods: dFLISA (red, green, and purple), ELISA (red), and dPCR (green). dFLISA was used to quantify both capsid and genomic titers and the FP ratio of AAV8 content in crude samples. ELISA was used to quantify capsid AAV8 content and used the SD calculated from independent duplicate measurements (*n* = 2). The dPCR was used to determine the AAV8 content in crude samples and used the SD derived from independent triplicate measurements (*n* = 3). Therefore, dPCR/ELISA (purple) was used to compare FP ratios. All data presented in these experiments are the averages used to compare dFLISA and dPCR/ELISA methods’ ability to quantify crude samples. The SD of each parameter was obtained from the triplicated experiments.

The purified sample, AAV8-Lot2, was first concentrated to final concentrations of 6.16 × 10^13^ cp/mL and 5.55 × 10^13^ vg/mL, and then diluted to three different concentrations: high (spike H) at 1.23 × 10^11^ cp/mL and 1.11 × 10^11^ vg/mL, medium (spike M) at 0.82 × 10^11^ cp/mL and 0.74 × 10^11^ vg/mL, and low (spike L) at 0.61 × 10^11^ cp/mL and 0.55 × 10^11^ vg/mL. The purified samples and crude lysate were mixed at a 1:1 ratio. The recovery of the spiked purified sample was evaluated as the difference between the high minus the middle (spike H − M) and the high minus the low (spike H − L) concentrations because the crude lysate contained an unknown amount of AAV particles. Recovered capsid titers of the spike H − M and spike H − L were 1.25 × 10^10^ cp/mL and 2.06 × 10^10^ cp/mL, respectively (Figure 4A), and the recovered genomic titers were spike H − M and spike H − L of 0.91 × 10^10^ vg/mL and 1.61 × 10^10^ vg/mL, respectively (Figure 4B). The FP ratios of spike H − M and spike H − L were 73.0% and 77.9%, respectively (Figure 4C). Spike recovery was consistently achieved across all mixed samples. Specifically, the capsid titer recovery rate was 122.1% ± 133.8%, and the genome recovery rate was 98.8% ± 115.7%. The obtained results met the criteria written in the materials and methods. The results suggest that the impurities in crude lysate do not interfere with capsid/genome quantification by dFLISA. dFLISA was then applied to the quantification of capsid and genomic titers of a crude sample. A dFLISA analysis of crude AAV yielded capsid and genome titers of 3.28 × 10^12^ cp/mL and 7.77 × 10^11^ vg/mL, respectively (Figure 5). There was no significant difference in capsid titer between dFLISA and ELISA (*p* = 0.303), which is a well-established method.^7^^,^^55^ In the genomic titer measurements, dFLISA showed significantly higher titers than dPCR (*p* = 0.029) (Figure 5).

### Application of dFLISA for quantifying different AAV serotypes

**Figure 6:**
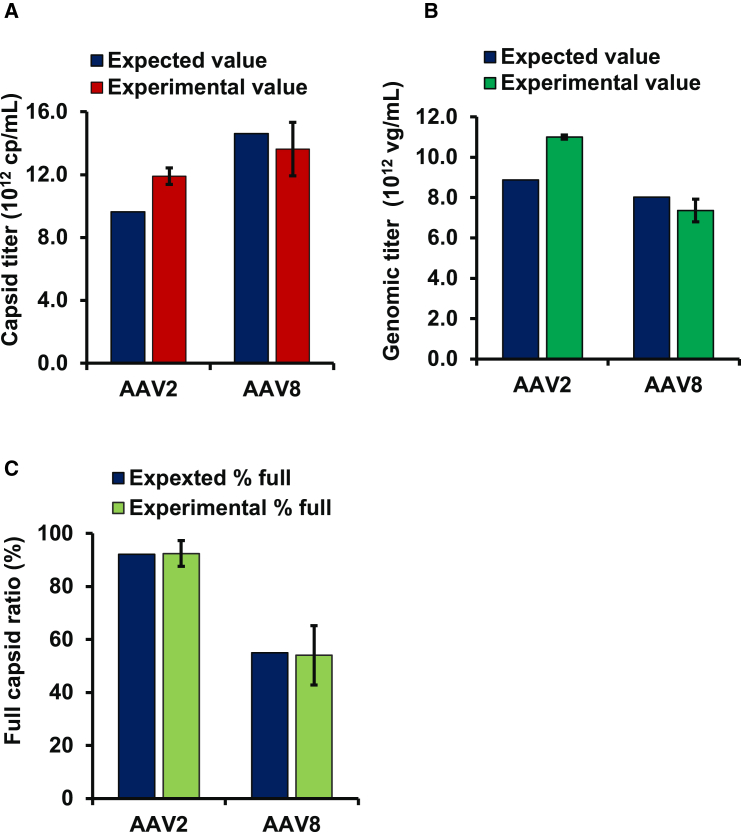
Application of dFLISA for quantification of different AAV serotypes (A) Capsid titer quantification. (B) Quantification of genome titer. (C) Detection of FP ratio. dFLISA experiments were performed in duplicate for AAV2 and AAV8 vectors. Expected values derived from BS-AUC (dark navy blue) were compared with actual values for three parameters, specifically capsid titer (red), genomic titer (green), and FP ratio (light green). Results are the average of duplicated wells.

Because VHH has been shown to bind to a broader range of serotypes (specifically, AAV1 to AAV8 and AAVrh10),^58^^,^^59^^,^^60^^,^^61^ which is sufficient for the needs of current clinical research,^62^ we further extended the applicability of the dFLISA method to facilitate the quantification of AAV of different serotypes. In this approach, the primary antibody was replaced by an antibody that specifically targets the serotype of interest. The capsid and genomic titers of AAV2-Lot4 were quantified by dFLISA using AAV2-Lot3 as a reference standard of AAV2-containing ssDNA. Figure 6 shows that the experimental values were consistent and comparable with the expected values (±25% of expected value). This suggests that the modified dFLISA method is effective in quantifying AAVs of different serotypes.

### Fluorescence intensity of SYBR gold with different genome lengths

**Figure 7:**
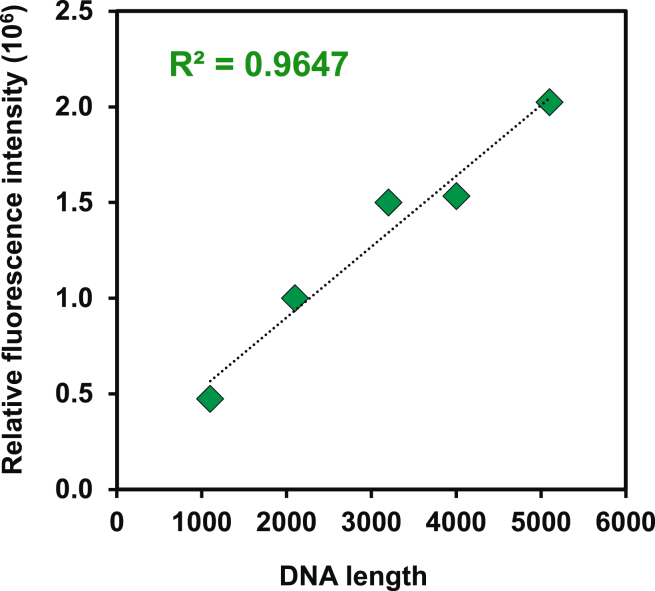
Comparison of fluorescence intensity of AAVs with different genome lengths The fluorescence intensities of different ssDNA lengths derived from AGE divided by the MP percentage area derived from the contrast histogram were plotted against the length of the ssDNA ladder standard. Correlation between SYBR gold fluorescence intensity and lengths of ssDNA ladder fragments. AGE, agarose gel electrophoresis; MP, mass photometry; scDNA, self-complementary DNA; ssDNA, single-stranded DNA.

We investigated the correlation between different AAV vector genome lengths and SYBR gold fluorescence intensity. First, a DNA mixture was analyzed by gel electrophoresis and dyed with SYBR gold. The intensity of each band was normalized to a relative concentration determined by MP and plotted against DNA length. As shown in Figure 7, the fluorescence intensity correlated well with genome length. AAV2 capsids containing different genome types, specifically self-complementary DNA (scDNA) (3,681 bases) and single-stranded DNA (ssDNA) (2,521 bases), were analyzed by dFLISA, and their fluorescence intensities were compared (Table S9). The fluorescence intensity of the ssDNA AAV2 vector was approximately 1.86 times lower than that of the scDNA AAV2 vector (Table S9), while the fluorescence intensity of ssDNA estimated from the curve (Figure 7) was approximately 1.4 times lower than that of scDNA.

## Discussion

This study aimed to establish a simple and reliable method of measuring AAV vector titers and the ratio of FP. We developed dFLISA, which uses two fluorescent dyes to quantify capsid and genome titers.^63^^,^^64^^,^^65^ The precision, accuracy, and quantification limits of dFLISA were assessed. In addition, the applicability of dFLISA for different detecting AAV vector serotypes and AAV vectors with different genome lengths were evaluated.

### Precision and accuracy of dFLISA analysis

The dFLISA method consistently yielded precision values below 15% across all tested samples while maintaining an accuracy of 80%–100% of the expected values for both capsid and genomic titers in samples 1–4, except in cases where the values approached or fell below the LOQ, as with samples 5–7 (Figure 2; Tables S3 and S4). This indicated the good precision and accuracy of our approach for obtaining capsid and genome titers, not only outperforming the combined dPCR and ELISA but also showing a significant improvement in error minimization. The relative concentrations of ExPs were relatively similar between the standard (AAV8-Lot1, 15.67%) and the sample (AAV8-Lot2, 12.95%) (Table S1). Because the genomic titer determined by dFLISA was the sum of FPs and ExPs, a difference in the relative concentrations of FPs and ExPs between standards and samples could result in inaccuracy and imprecision. Although the PP concentrations of samples used were lower than the LOQ of BS-AUC^63^ and were ignored in this study, influence of PPs on the capsid and genomic titers should be considered carefully. dFLISA can simply quantify capsid and genomic titers; however, the inability to distinguish FPs from ExPs is the limitation of dFLISA. For detailed characterization, analyses using orthogonal methods that can distinguish between FPs, EPs, PPs, and ExPs are desired.

We then calculated the LOQ, whose values for dFLISA were determined based on assay precision, accuracy, and background noise. It is remarkable that the LOQ values were close to those expected from the precision, accuracy, and the standard curve. Even the LOQ of dFLISA was only slightly higher than the LOQ of the ELISA. Nonetheless, it is sufficient to identify the capsid titers. Intermediate species cannot be quantified. It also indicates that further studies are needed to improve the sensitivity of the method for more accurate and precise detection and quantification of the analyte.

### Linearity of AAV FP ratio in dFLISA analysis

dFLISA showed robust correlation and linearity in the FP ratio. The experimental ratio of full AAV particles was 0%–85.8%, with a precision of %CV 3.26% ± 25% (Figure 3), demonstrating good agreement with the expected FP ratio of 0%–90.1%. In addition, linearity experiments were performed using dFLISA over a range of ratios. Therefore, the reliable performance of this technique highlights its ability to discriminate between the different ratios of FPs. Furthermore, during the dFLISA demonstration, we improved the reliability and robustness of the method over multiple runs by introducing AAV vector samples at different concentrations and adjusting the FP ratio. This optimization not only minimized the duration of each assay, but also ensured a high level of consistency.

### Comparison of dFLISA and orthogonal methods using mixed samples

The main approach for determining both capsid and genomic titers is to choose the most suitable analytical method for absolute quantification. BS-AUC is a standard technique used to analyze capsid content and distinguish between empty and full capsids as well as other AAV vector subspecies.^47^^,^^57^ BS-AUC is based on the differential sedimentation velocities of AAV vector subpopulations under strong centrifugal force due to differences in size, density, weight, and shape. A combination of dPCR and ELISA is another standard method for the determination of the FP ratio. MP has recently gained popularity for AAV vector characterization because of its mass resolution, which allows operators to discriminate between empty and genome-filled capsids.^54^^,^^64^^,^^65^

In our study, we conducted a comparative analysis of the FP percentages in identical recombinant AAV samples via dFLISA, BS-AUC, MP, and dPCR/ELISA. The FP ratio determined by dFLISA was closer to the expected values than that determined by BS-AUC, MP, and dPCR/ELISA. The genomic titer determined by BS-AUC was lower than expected values (Figure S4), and the FP ratio determined by BS-AUC was lower than that of dFLISA (Figure 3). It should be noted that FP ratio was calculated by dividing the sum of FPs and ExPs by the sum of EPs, FPs, and ExPs and different from an FP ratio calculated only from EPs and FPs.^63^^,^^66^

Two peaks with mass corresponding to EPs and FPs were observed in MP analysis, and an ExP-related peak was not observed (Figure S5). A Gaussian distribution fit was applied to the two histogram peaks and FP ratio was calculated based on the peak area. The results of MP were consistent with that of dFLISA and both were as expected, with the exception of one sample containing 10% FPs. MP did not detect any EPs in the sample with 10% FPs, suggesting that dFLISA has a higher sensitivity than MP. The advantages and limitations of analytical methods used in this study are summarized in Table 1.

**Table 1:** Most crucial performance criteria of analytical methods in this study

Methods	Target information	Purified sample	Turnaround (h)^a^	Sample volume (μL)	Sample concentration	Advantages	Limitations
BS-AUC	particle content and aggregate	yes	4–5	15–30	1 × 10^12^–2 × 10^13^ cp/mL	capable of quantifying partially filled capsids and aggregates	requires specialized equipment purification is required prior to analysis of crude samples
dPCR	genomic titer	no	2–3	25	10^7^–10^10^ vg/mL	specific and fast	low precision and accuracy
dFLISA	particle content	no	4.5–5	100	2 × 10^10^–10^11^ vg/mL	high precision and accuracy purification is not necessary prior to analysis of crude samples	LOQ is slightly higher than the LOQ of ELISA; however, it is high enough to detect capsid titers PPs and ExPs cannot be distinguished from FPs
ELISA	capsid titer	no	4.5–5	100	10^8^–10^10^ cp/mL	high specificity	low accuracy and precision due to two independent analyses
MP	particle content	yes	0.33–0.5	110	1 × 10^11^–1 × 10^12^ vg/mL	capable of quantifying partially filled capsids, impurities, and aggregates low material requirements	requires specialized equipment purification is necessary prior to the analysis of crude samples

BS-AUC, band sedimentation analytical ultracentrifugation; dPCR, digital polymerase chain reaction; dFLISA, dual fluorescence-linked immunosorbent assay; ELISA, enzyme-linked immunosorbent assay; MP, mass photometry; ExP, extra filled particle; FP, full particle; LOQ, limit of quantification; PP, partial particle.

a Turnaround time includes sample preparation and data analysis.

Previous studies have reported evidence showing that high-performance liquid chromatography (HPLC) is a rapid and convenient method for analyzing the empty and full capsid content of purified AAV samples.^67^^,^^68^ The sensitivity of HPLC was sufficient to quantify the empty and full AAV vectors in samples with capsid concentrations as low as ∼5 × 10^10^ cp/mL,^68^ whereas dFLISA was able to quantify empty and full AAV vectors at concentrations as low as 0.60 × 10^10^ cp/mL (Table S5). Notably, the sensitivity of dFLISA was higher than that of HPLC, although HPLC is a promising method to determine FP ratio.

For capsid titer quantification, dFLISA demonstrated consistency with the ELISA (Table S7), but the LOQ of dFLISA (Table S5) was higher than that of traditional ELISA. This was probably due to differences in the detection method—the ELISA uses a horseradish-peroxidase-conjugated streptavidin enzyme^37^^,^^69^ while dFLISA uses a second antibody conjugated to red fluorescence for capsid titer quantification. For genomic titer measurement, dFLISA showed higher titers than dPCR for samples with 10.5%–90.1% FPs. This might be due to the variability of dPCR,^70^^,^^71^ and, considering that the FP ratio determined by dFLISA was close to the expected values, genomic titer quantification by dFLISA should be more precise and accurate than dPCR. Another possible reason why the dPCR result was lower than the dFLISA result is that ExPs could contain a genome without inverted terminal repeat (ITR), and thus dPCR could not quantify ExPs because dPCR specifically quantifies a genome with ITR. The specificity of dFLISA was only for a genome, not a genome with ITR. The non-specificity is considered to be another limitation of dFLISA.

Comparing the methods from the perspective of the operator, it is important to note that BS-AUC and MP require specialized equipment and high capsid titers.^57^ Conversely, the reliability of dFLISA as an alternative analytical method for the precise assessment of the FP ratio that uses the same standard has been demonstrated. Thus, it is a crucial test for quantifying FP, EP, and FP ratios. In FP ratio analysis by dPCR/ELISA, data from two independent analyses are required. Therefore, the combination method generally has inherently higher variability. Moreover, dPCR-based methods show higher variability than ELISA.^72^

The recovery efficiency of spiking levels was also assessed to see whether dFLISA can be used to analyze crude samples without purification. The recovery percentage was within ±25% of the expected values, which meets the criteria for acceptance. This suggests that the results of dFLISA are not affected by the matrix interferences contained in crude samples.^73^^,^^74^ This highlights the suitability of the dFLISA method as a way to evaluate AAV samples that have not been purified, which offers a noteworthy advantage. Based on the results obtained, it is reasonable to conclude that the dFLISA method is well suited to the quantification of unpurified AAV vector samples. Therefore, dFLISA serves as a valuable and novel method that can be used to accurately quantify the titers of crude samples, making it uniquely capable of directly quantifying the capsid and genomic titer and FP ratio of crude samples.

While AUC requires the purification of crude samples prior to analysis, the capsid and genomic titers of untreated crude samples can be measured by dFLISA. The capsid titer determined by dFLISA was comparable with that determined by ELISA. However, the genomic titer results with dFLISA were higher than those from dPCR. The recovery rate in the spike-recovery experiment of dFLISA was high, whereas the spike-recovery result of dPCR was lower than expected values (Figure S6). In addition, previous studies demonstrated that dPCR can be affected by the interference of impurities.^70^^,^^71^^,^^72^^,^^75^^,^^76^^,^^77^^,^^78^^,^^79^^,^^80^ This suggests that dFLISA results are relatively unaffected by matrix interference or impurities from the crude lysate, making it a reliable analytical technique for AAV vector particle analysis. The optimization of the dPCR method could provide better results, which is nevertheless beyond the scope of this study.

Considering the LOQ of dFLISA (1.61 × 10^10^ cp/mL, 1.70 × 10^10^ vg/mL) and the concentration of AAV at the end of upstream process (>10^10^ vg/mL), the sensitivity of dFLISA is high enough to analyze crude samples, although it would be difficult to analyze samples at the beginning of the upstream process.

The dPCR/ELISA combination approach is time-consuming and exhibits low accuracy, with reported coefficients of up to 36%.^74^^,^^81^ In contrast, the entire dFLISA run was completed in less than 5 h. The simple data evaluation procedure contributes to the short duration, allowing for the analysis of more than 35 samples per day. Furthermore, the dFLISA allows for the straightforward quantification of purified AAV vectors as well as unpurified in-process samples. This is especially critical because there are no direct orthogonal methods available for quantifying crude samples without any purification.

### Application of dFLISA analysis for diverse AAVs

We next aimed to expand the capabilities of dFLISA in this study. We thus demonstrated the suitability of the technique for quantifying the capsid and genomic titers for other AAV vector serotypes. The results show that dFLISA can be applied to other serotypes via a modification to the primary antibody.

To optimize our dFLISA, we used 85°C as the optimal temperature to disrupt the AAV capsid, and the appropriate temperature range for the disruption of AAV capsid particles AAV1 to AAV8 was between 66.5°C and 89.5°C ± 0.5°C, with the exception of AAV5. As indicated in previous studies,^82^ the specific temperature requirement for AAV5 disruption was 90°C ± 0.5°C. In addition, in our experiment, it was possible to use the AAV9 vector by simply modifying the ligand that coats the microtiter plate with the vector because the binding affinity of the VHH coating antibody is limited to the AAV9 vector.

The fluorescence intensity of the AAV vector varies with different genome lengths, and this factor is also relevant to dFLISA. All AAV vectors used in other experiments had genome lengths almost identical to that of the standard AAV vector. In addition, a comparison of fluorescence intensity between AAV vectors with different genome lengths validated the value and reliability of dFLISA as a method for evaluating AAV vector genomes. First, we used a mixture of ssDNA to evaluate the correlation between fluorescence intensity and DNA length. As shown in Figure 7, the relative fluorescence intensities were proportional to the genome lengths. Then, the differences in fluorescence intensities between two AAV vectors with different genome lengths (2,521 and 3,681 bases) were evaluated. According to the curve derived from the mixed DNA samples (Figure 7), the ratio of fluorescence intensity of the AAV with scDNA to that of the AAV with ssDNA was expected to be 1.40; however, the experimental value was 1.86 (Table S9). Therefore, although the fluorescence intensity of the ssDNA mixture was proportional to genome length, an interaction of DNA released from an AAV capsid with SYBR gold could be genome dependent probably due to the high temperature for capsid disruption and/or tertiary structure of DNA. If the length of an AAV genome is different from that of the standard AAV vector, we need to evaluate the difference in SYBR gold intensity between the sample and the standard prior to dFLISA analysis.

### Conclusion

Our study introduces a novel analytical technique that allows for the accurate and precise measurement of the abundance of both full and empty AAV vector capsids, and the full/empty capsid ratio. The correlation between dFLISA and BS-AUC proved robust, indicating the reliability of the dFLISA results for both full and empty capsids. The dFLISA results also corresponded with those of other orthogonal techniques, including MP and a combination of dPCR and ELISA. Remarkably, dFLISA showed significant potential for evaluating the capsid and genome titers of unpurified samples and diverse AAV vector serotypes, offering versatility and eliminating the need for high levels of analytical expertise. Thus, the newly developed dFLISA presented in our paper has proven to be an invaluable method for the straightforward, accurate, and precise detection of AAV vector particles. Its potential utility offers significant opportunities for the advancement of AAV-based gene therapies.

## Materials and methods

### Materials

#### Recombinant AAVs

All rAAV vectors, including AAV8-Lot1, AAV8-Lot2, and AAV8-Lot3, were generated using triple-plasmid co-transfection. In brief, pAAV-Rep&Cap (Serotype 8), pAd helper, and transgene plasmids (CMV-EGFP or AAT-FIX) were co-transfected into suspended HEK293T or VPC 2.0 (Thermo Fisher Scientific, Waltham, MA) cells. The transfected cells were cultured, and the medium and cell lysate were harvested (it was collected as a crude sample). Thereafter, the samples were purified via affinity chromatography using AAVX columns (Thermo Fisher Scientific). Bulk AAV samples were purified using affinity chromatographic purification followed by a CsCl ultracentrifugation (UC) or an anion exchange column to separate full and EPs. Purified samples (AAV8-Lot1 and AAV8-Lot2) were centrifuged at 25,000 rpm in an Optima XE-90 (Beckman Coulter, Brea, CA) using a Beckman SW41Ti rotor at 20°C for 42 h. For AAV8-Lot3, the purified sample was centrifuged at 34,000 rpm at 20°C for 72 h. The virus bands generated by UC were collected by using a piston fractionator (BioComp Instruments, Fredericton, Canada) equipped with a UV monitoring apparatus (Triax flow cell, BioComp Instruments). For the anion exchange chromatography, the samples were applied to a CIMmultus QA column (Sartorius, Göttingen, Germany) and eluted with a linear gradient of 0–250 mM NaCl in bis-tris-propane buffer (pH 9.0). Then the virus fractions were dialyzed in Slide-A-Lyzer 10K (Thermo Fisher Scientific). AAV samples were analyzed by BS-AUC prior to analysis (Figures S1–S3). Table S1 summarizes the information on the in-house AAV8 vectors used in this study. We also used other laboratory-grade AAV vectors, including AAV2-Lot1 to AAV2-Lot4 and AAV8-Lot5 to AAV8-Lot6 manufactured in HEK293T cells, which were procured from VectorBuilder (Chicago, IL). Table S2 summarizes the information on the commercial AAV2 vector and AAV8 vector used in the dFLISA experiments.

#### Reagents

CaptureSelect Biotin Anti-AAVX Conjugate (VHH) and BupH carbonate-bicarbonate buffer packs were purchased from Thermo Fisher Scientific. Lyophilized mouse anti-AAV2 monoclonal antibody (A20) and lyophilized mouse anti-AAV8 monoclonal antibody (ADK8) were purchased from Progen, (Heidelberg, Germany). Goat anti-mouse IgG H&L (Alexa Fluor 647) was purchased from Abcam (Cambridge, UK). SYBR Gold Nucleic Acid Gel staining solution was purchased from Invitrogen, Thermo Fisher Scientific (Eugene, OR). BSA and Tween 20 were purchased from Sigma Aldrich (St. Louis, MO). Sodium chloride (NaCl), purchased from BASF (Ludwigshafen, Germany), and PBS, purchased from Thermo Fisher Scientific, were used for buffer and solution preparation. Poloxamer-188 was kindly provided from BASF.

### Methods

#### dFLISA

dFLISA was performed as shown in Figure 1. Black, 96-well, flat-bottomed MaxiSorp surface-treated immunoplates (Thermo Fisher Scientific) were used. These plates were coated with CaptureSelect Biotin Anti-AAVX Conjugate, a 14-kDa recombinant single-domain antibody fragment (VHH affinity ligand), at a concentration of 10 μg/mL (100-fold dilution) with BupH carbonate-bicarbonate buffer and the plates were incubated for 16 h at 4°C. The plates were washed three times with 0.05% Tween 20 in 1× PBS (pH 7.4). Prior to sample addition, addition of 200 μL of 1% BSA in 1× PBS was performed for blocking. An AAV vector sample in a formulation consisting of 1× PBS, 200 mM NaCl, and 0.001% (w/v) poloxamer-188 was used as sample. AAV8-Lot1 with a 2,521-bp genome was used as a standard. AAV8-Lot1 was diluted to a concentration of 2.38 × 10^11^ cp/mL and 2.18 × 10^11^ vg/mL in 0.05% Tween 20 in 1× PBS and then serially diluted at a 1:2 ratio to generate a calibration curve. Subsequently, 100 μL of sample and standard solutions were added to each well of the plate. To remove unbound components, the plate was subjected to a wash with 0.05% Tween 20 in 1× PBS. The lyophilized monoclonal anti-AAV8 antibody, ADK8, was reconstituted with 1 mL of Milli-Q water. ADK8 was diluted to a concentration of 1 μg/mL (50-fold dilution) with 1× PBS buffer at pH 7.4 containing 0.09% sodium azide and 0.5% BSA, followed by incubation at 37°C for 1 h, and three washes. The secondary antibody, goat anti-mouse IgG H&L (Alexa Fluor 647), was diluted to a concentration of 4 μg/mL (500-fold dilution) and added for labeling. The plate was then sealed with adhesive foil and incubated for 1 h at 37°C with shaking at 300 rpm. Following incubation, the plate was washed with wash buffer. Next, 100 μL of 1× PBS was added to each well, and plates were incubated at 85°C for 15 min. This process disrupted the viral capsid structure and released the ssDNA. Afterward, samples were allowed to cool at room temperature for 5 min. SYBR Gold Nucleic Acid Gel Stain solution was diluted 1,000-fold with 1× PBS. Subsequently, 10 μL of the diluted SYBR gold solution was added to individual wells, followed by a 5-min incubation at room temperature. Finally, we measured the intensity of the red fluorescence emanating from the proteins labeled with goat anti-mouse IgG H&L (Alexa Fluor 647) to quantify the capsid titers using an excitation wavelength of 652 nm and an emission wavelength of 680 nm. In addition, we measured the intensity of the green fluorescence emanating from SYBR gold^56^^,^^83^^,^^84^ to quantify the released genome using an excitation wavelength of 500 nm and an emission wavelength of 530 nm. A standard curve was generated using a four-parameter curve-fitting algorithm with the SpectraMax i3x microplate reader from Molecular Devices (San Jose, CA). Capsid and genomic titers of standards were calculated based on the results of BS-AUC. Because the concentrations of PPs were lower than LOQ of BS-AUC,^63^ a sum of EPs, FPs, and ExPs was considered as capsid titer, and a sum of FPs and ExPs was considered as genomic titer. FP ratio was then calculated by dividing genomic titer by capsid titer. The amounts of cp/mL and vg/mL were then determined using the standard curve. Correction of SYBR gold intensity was not performed if the percent difference in genome length between the standard and the sample was within ±10%.

### Quantification of capsid and genomic titers by dFLISA

Similarly, AAV8-Lot2 with a 2,712-base gene of interest was diluted 400-fold to a concentration of 1.54 × 10^11^ cp/mL and 1.39 × 10^11^ vg/mL in 0.05% Tween 20 in 1× PBS. Subsequently, serial dilutions at a 1:2 ratio were carried out to ensure precision, accuracy, and LOQ calculations. Measurements were conducted for three consecutive days (days 1–3) under identical operating conditions at short intervals using the same sample conditions and without any freeze-thaw cycles. The repeatability percentage was calculated by dividing the SD by the mean of three independent dFLISA results obtained by the same operator over 3 days (Equation 1):

(Equation 1) $$\%\text{CV}=\frac{\left[\text{Mean}\;\text{of}\;\text{SD}\right]}{\left[\text{Mean}\;\text{of}\;\;\text{result}\right]}\times 100$$

Accuracy was calculated by finding the percentage difference between the value of capsid and genomic titers, as measured by dFLISA, and their expected values, as determined by BS-AUC.

Equation 2 was used with ±10% of expected values as the recovery percentage criterion for this calculation:

(Equation 2) $$\%\;\text{of}\;\text{accuracy}=\frac{\left[\text{Experimental}\;\text{value}\right]}{\left[\text{Expected}\;\text{value}\right]}\times 100$$

### Determination of FP ratio by dFLISA

Linearity of FP ratio is critical in dFLISA to achieve optimal assay performance. We investigated precision, accuracy, linearity, and LOQ of FP ratio determined by dFLISA. The representative dFLISA procedure described above was used. Specifically, AAV8-Lot1 was diluted 60-fold to obtain a concentration of 2.38 × 10^11^ cp/mL and 2.18 × 10^11^ vg/mL with 0.05% Tween 20 in 1× PBS. Further serial dilutions were performed at a 1:2 ratio to construct a calibration curve. AAV8-Lot2 and AAV8-Lot3 were concentrated by ultrafiltration. We then mixed these concentrated samples at various ratios containing the following expected percentages of full capsids: 0%, 10.5%, 31.5%, 52.3%, 73.1%, and 90.1% of FPs. The mixed sample was then diluted 320-fold with 0.05% Tween 20 in 1× PBS. All samples were tested in duplicate. Each well of the plate was filled with 100 μL of the prepared sample solutions. The back-calculated concentrations of the calibration standards were maintained within ±25% of the value at the LOQ and within ±20% at all other levels.^73^ The anchor calibrators (<LOQ) did not require acceptance criteria because they were beyond the quantifiable range of the curve.

### Determination of full-to-empty ratio by BS-AUC

Experiments and analyses of BS-AUC were performed according to our previous study.^63^ In brief, a buffer or AAV sample at a volume of 15 μL were loaded into a reference or sample reservoir well with a 12-mm band-forming centerpiece (Spin Analytical, South Berwick, ME) equipped with sapphire windows. A volume of 250 μL of PBS/D_2_O containing 0.001% of poloxamer-188 was loaded into the reference or sample sector, respectively. Mixed samples (FPs in six prepared spike ratios, specifically, 90.1%, 73.1%, 52.1%, 31.5%, 10.5%, and 0% FPs) of AAV8 vectors were used. Data were collected at 20°C using Optima AUC (Beckman Coulter) at 20,000 rpm using a UV detection system, with the detection wavelength set at 280 nm. Data points were collected with a radial increment of 10 μm at an interval of 150 s

Sedimentation data were analyzed using the analytical zone centrifugation c(s) model of the program SEDFIT (version 16.2b),^85^ in which parameters such as lamella width, frictional ratio, meniscus, time-invariant noise, and radial-invariant noise were adjusted and a regularization level of 0.68 was used. The s value range of 0–175 S was evaluated with a resolution of 350. The SEDNTERP program facilitated the calculation of buffer density and viscosity for the solvent loaded in the sectors.^86^ The apparent sedimentation coefficient for FPs was converted to the sedimentation coefficient in water at 20°C (*s*_20,w_). This conversion used the partial specific volume of the FPs, determined according to the procedure described in a previous study,^66^ in conjunction with the buffer density and buffer viscosity. Subsequently, figures showing the c(s) distribution were generated using the program GUSSI (version 1.3.2).^87^

Particle concentrations were calculated by dividing the FP, EP, and ExP peak areas by respective molar extinction coefficient at the detection wavelength. The FP ratio was calculated by dividing the sum of FPs and ExPs by the sum of FPs, EPs, and ExPs. The mean *s*_20w_, FP ratio, and SD of each parameter were calculated based on the results obtained from the triplicate experiments.

### Determination of capsid titers of AAV8 vector by ELISA

An AAV8 titration kit (Progen) was used to determine capsid titers. The assay was performed according to the manufacturer’s instructions. A series of 2-fold dilutions of the kit’s standard viruses were made to generate a capsid standard curve ranging from 7.97 × 10^6^ to 5.01 × 10^8^ cp/mL. Mixed samples (full capsids in six prepared spike ratios, specifically, 90.1%, 73.1%, 52.3%, 31.5%, 10.5%, and 0% FPs) of AAV8 vector were diluted with 0.05% Tween 20 in 1× PBS. All measurements, including unknown samples and blanks, were performed in duplicate at three different dilutions. The mean value was used to calculate AAV8 titers. A prepared 100 μL sample was added to a microwell plate and incubated for 1 h at 37°C. The microwell plate was then washed three times with wash buffer. The biotinylated anti-AAV8 antibody (ADK8) was then added to the microwell plate, and the plate was incubated for 1 h at 37°C. The washing step was repeated. Streptavidin-horse radish peroxidase conjugate was added and incubated for 1 h at 37°C, followed by the washing and the addition of ready-to-use tetramethylbenzidine solution to the wells, which were then incubated for 15 min at room temperature. The color reaction was stopped by adding ready-to-use sulfuric acid solution. Absorbance was then measured photometrically at 450 nm with a SpectraMax 3× microplate reader. The readings of each sample were then averaged to determine the final titers using a four-parameter logistic (4PL) curve-fitting model. The 4PL standard curve was generated in Microsoft Excel by plotting the subtracted optical density measurements of the serially diluted kit controls against the corresponding AAV vector concentrations.

### Determination of genomic titers of mixed samples of AAV8 vector using dPCR

AAV vector samples with various FP ratio (full capsids in six prepared ratios, specifically, 90.1%, 73.1%, 52.3%, 31.5%, 10.5%, and 0% FPs) were prepared as described above and then treated with DNase I (Takara, Japan). The samples were then incubated at 37°C for 30 min to digest any unpackaged DNA. Subsequently, a solution of 0.25 mM ethylenediaminetetraacetic acid (EDTA) (Nippon Gene, Japan) was added. The mixture was incubated at room temperature for 5 min. Afterward, the mixture was heated to 95°C for 15 min to inactivate the DNase I enzyme and denature the viral capsid. Dilution buffer was prepared by adding poloxamer-188 to Tris-EDTA buffer to achieve a final concentration of 0.001%. This dilution buffer was used to dilute the test samples to the appropriate range for analysis. Each dPCR reaction was set up to a final volume of 10 μL consisting of 1 μL of the prepared sample solution, 2 μL of 5× dPCR QuantStudio Absolute Q Master Mix (Thermo Fisher Scientific), 1.8 μL ITR primers (forward and reverse), and 0.25 μL ITP probe mix (purchased from Hokkaido System Science); 9 μL of dPCR reaction mix was added to each well of a QuantStudio Absolute Q MAP16 Plate Kit (Thermo Fisher Scientific). Afterward, 15 μL of QuantStudio Absolute Q Isolation Buffer (Thermo Fisher Scientific) was carefully added to each well on top of the reaction mix. The wells were sealed with QuantStudio Absolute Q Strip Caps (Thermo Fisher Scientific) and centrifuged at 1,200 rpm for 1 min on a swing-out rotor. The assay was performed on a QuantStudio Absolute Q Digital PCR System (Thermo Fisher Scientific). Thermal cycling was performed as follows: (1) preheat at 96°C for 10 min, then (2) 40 cycles consisting of denaturation at 94°C for 5 s followed by annealing/extension at 54°C for 30 s. Data and global threshold were analyzed using QuantStudio Absolute Q digital PCR software (Thermo Fisher Scientific). Sample dilutions were used to calculate AAV genomic titers.

### Determination of FP ratios of AAV8 vector by MP

MP measurements were conducted using TwoMP (Refeyn, Oxford, UK). For each experiment, mixed samples (full capsids at six prepared sample ratios, specifically, 90.1%, 73.1%, 52.3%, 31.5%, 10.5%, and 0% FPs) of AAV8 vector were pre-diluted in PBS (Gibco). Precision cover glasses (ThorLabs, Tokyo, Japan) were meticulously cleaned by serial rinsing with Milli-Q water and ethanol. To create the measurement chambers, we attached a pre-cut 2 × 3 well CultureWell silicone seal (3 mm diameter × 1 mm depth, Grace Bio-Labs, Bend, OR) to the clean coverslips. The coverslips were then transferred to the MP instrument, and 18 μL of PBS buffer was added to each well. After focusing, 2 μL of each AAV vector solution was added and mixed into the wells to achieve a total filling volume of 20 μL. Each measurement was recorded for 60 s, and each sample was analyzed at least three times (*n* ≥ 3). Data analysis was performed using DiscoverMP version 2.5.1 (Refeyn). A Gaussian distribution fit was applied to the histogram peaks. From these Gaussian fits, we extracted the percentage of filled and empty AAV capsids.

### Quantification of crude sample by dFLISA and other methods

A representative method for dFLISA is described above. In these experiments, AAV8-Lot1 was used as standard as described above. For the spike sample, AAV8-Lot2 was concentrated by ultrafiltration to reach a final concentration of 6.16 × 10^13^ cp/mL and 5.55 × 10^13^ vg/mL. It was then diluted to three different spiking levels: high (spike H) at 1.23 × 10^11^ cp/mL and 1.11 × 10^11^ vg/mL, medium (spike M) at 0.82 × 10^11^ cp/mL and 0.74 × 10^10^ vg/mL, and low (spike L) at 0.61 × 10^11^ cp/mL and 0.55 × 10^11^ vg/mL, all in 0.05% Tween 20 in 1× PBS. A crude lysate sample of unknown concentration was mixed with each spike sample solution at a 1:1 volume ratio and analyzed in duplicate. The following equations were used to calculate the expected values for mixed spike samples:

(Equation 3) $$\text{Mixed}\;\text{spike}\left(H–M\right)=\frac{1}{2}\left(\text{crude}+\text{spike}\;H\right)–\frac{1}{2}\left(\text{crude}+\text{spike}\;M\right)$$

(Equation 4) $$\text{Mixed}\;\text{spike}\;\left(H–M\right)=\frac{1}{2}\left(\text{crude}+\text{spike}\;H\right)–\frac{1}{2}\left(\text{crude}+\text{spike}\;L\right)$$

The prepared spike sample solutions were then added to each well of the plate. The target recovery percentage (%) was within ±25%.

### Quantification of capsid and genomic titers of crude samples

For this experiment, the representative dFLISA method described above was used. An AAV8 crude lysate sample of an unknown concentration was analyzed by dFLISA, as detailed in the preceding section. The analysis was performed in triplicate. The capsid and genomic titers obtained by dFLISA were compared with ELISA and dPCR results by independent samples t test.

### Application of dFLISA for quantifying different AAV serotypes

The representative dFLISA method described above was used for this experiment. AAV2-Lot3, which contained linear ssDNA, was diluted 100-fold to reach concentrations of 1.77 × 10^11^ cp/mL and 1.65 × 10^11^ vg/mL with 0.05% Tween 20 in 1× PBS, then serially diluted 1:2 to generate a calibration curve. AAV2-Lot4 was also diluted 100-fold, resulting in final concentrations of 9.63 × 10^10^ cp/mL and 8.88 × 10^10^ vg/mL, with 0.05% Tween 20 in 1× PBS. The AAV8-Lot5 sample containing linear ssDNA was diluted 50-fold to reach concentrations of 2.06 × 10^11^ cp/mL and 1.59 × 10^11^ vg/mL with 0.05% Tween 20 in 1× PBS, and then serially diluted 1:2 to generate a calibration curve. AAV8-Lot6 was diluted 50-fold to achieve final concentrations of 2.92 × 10^11^ cp/mL and 1.60 × 10^11^ vg/mL with 0.05% Tween 20 in 1× PBS. Test samples prepared without any freeze-thaw cycles.

### Fluorescence intensity with different genome lengths

A ssDNA 7K ladder (PerkinElmer, Waltham, MA) containing ssDNA fragments ranging from 1,100 bases to 5,100 bases was used. First, 2 μL of EzApplyDNA (6× loading buffer) was applied to Parafilm for each sample. Next, 10 μL of sample was added and thoroughly mixed by pipetting, and 10 μL of the mixture was loaded onto a 1% agarose gel (Funakoshi, Japan). Electrophoresis was performed at 70 V for 45 min, after which the gel was stained according to the manufacturer’s instructions. SYBR Gold Nucleic Acid Gel staining solution (Invitrogen, Thermo Fisher Scientific, Eugene, OR) was used for gel staining. Quantitative analysis of brightness density within the stained gel was performed using an iBright 1500 instrument (Thermo Fisher Scientific) in conjunction with iBright Analysis version 4.0 software (Thermo Fisher Scientific). Image brightness adjustments were made prior to analysis.

Subsequently, the ssDNA ladder was analyzed by MP to determine the relative concentration of each ssDNA in the ladder. Before conducting MP measurements, ssDNA ladder solutions were diluted in buffer consisting of 5 mM Tris and 10 mM MgCl_2_ (pH 8). Each measurement was recorded for 60 s, and every sample was examined a minimum of three times (*n* ≥ 3). Data analysis was conducted using DiscoverMP and an in-house Python program. Histogram peaks were fitted with Gaussian distributions to extract the percentage of ssDNA ratio.

Then, the fluorescence intensities obtained by agarose gel electrophoresis with five different ssDNA ladder strands were divided by MP area (%) to obtain relative fluorescence intensity per molecule. The relative fluorescence intensity was plotted against the ssDNA ladder standard length. The expected fluorescence intensity ratio of the AAV vectors with both scDNA (3,681 bp) and ssDNA (2,521 bp) was also estimated from the curve.

The fluorescence intensity of AAV-containing scDNA was examined using dFLISA and compared with that of AAV-containing ssDNA. AAV2-Lot1-containing ssDNA was diluted 100-fold to concentrations of 3.09 × 10^10^ cp/mL and 2.82 × 10^10^ vg/mL in 0.05% Tween 20 in 1× PBS, and then serially diluted at a 1:2 ratio to generate a calibration curve. Similarly, AAV2-Lot2-containing scDNA was diluted 50-fold to concentrations of 7.47 × 10^10^ cp/mL and 6.66 × 10^10^ vg/mL in 0.05% Tween 20 in 1× PBS and then serially diluted at a 1:2 ratio to generate a calibration curve. The dFLISA analysis method as described earlier in the methods was used. The ratio of fluorescence intensities of ssDNA AAV8 and scDNA AAV2 were calculated.

## Data and code availability

Data will be made available on request.

## Supplemental information

Document S1. Figures S1–S7 and Tables S1–S9

Document S2. Article plus supplemental information
