# Supplementary material for: Quantification of full and empty particles of adeno-associated virus vectors via a novel dual fluorescence-linked immunosorbent assay
Source: Mol Ther Methods Clin Dev. 2024 Jun 24;32(3):101291. doi: 10.1016/j.omtm.2024.101291 (PMC11283060; doi:10.1016/j.omtm.2024.101291)
Supplement: Document S1. Figures S1–S7 and Tables S1–S9 [file mmc1.pdf]

OMTM, Volume *32*

Supplemental information

Quantification of full and empty particles of adeno-associated virus vectors via a novel dual fluorescence-linked immunosorbent assay

Sereirath Soth, Mikako Takakura, Masahiro Suekawa, Takayuki Onishi, Kiichi Hirohata, Tamami Hashimoto, Takahiro Maruno, Mitsuko Fukuhara, Yasuo Tsunaka, Tetsuo Torisu, and Susumu Uchiyama

Supplemental Figures

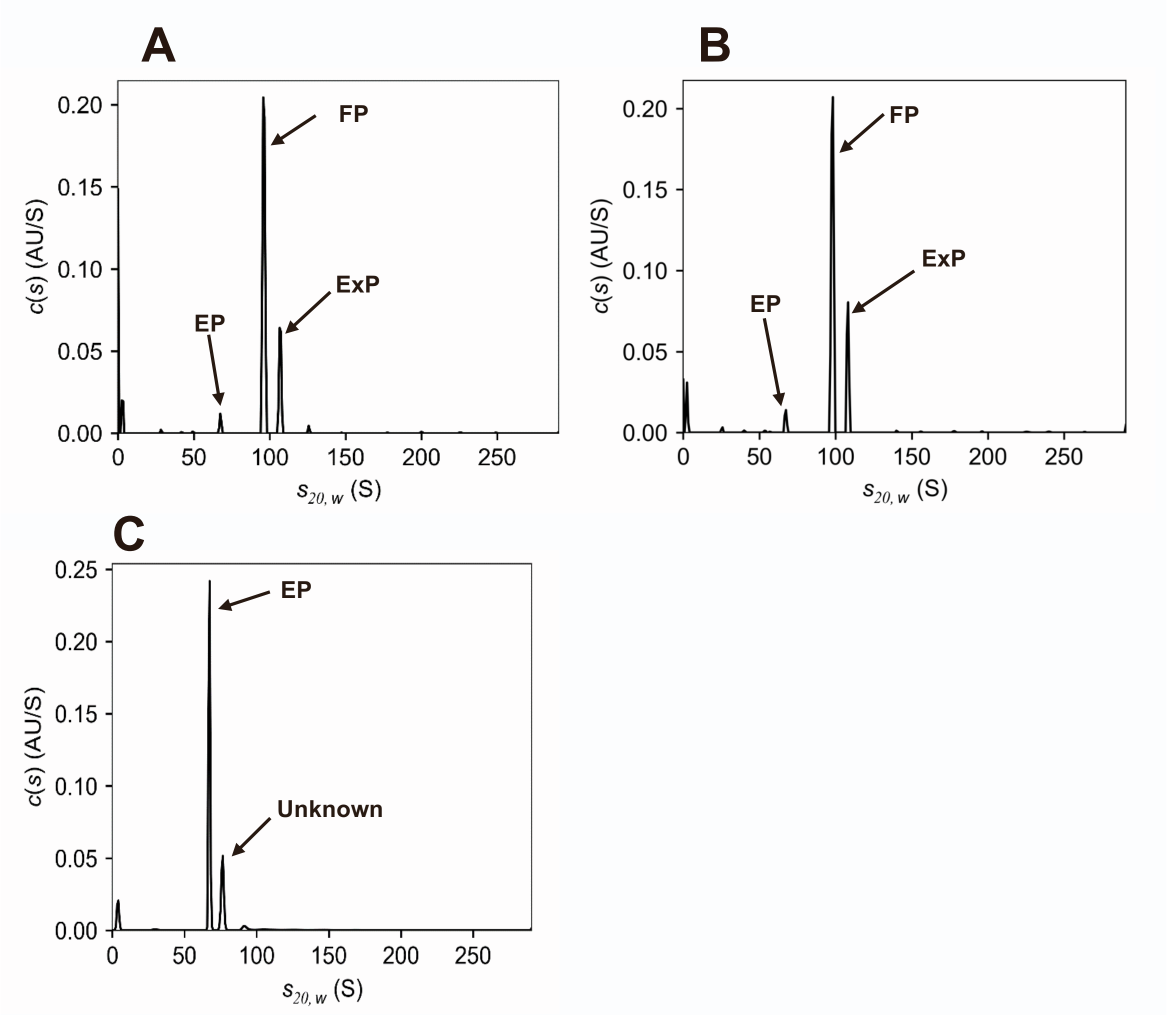

Figure S1. **Representative sedimentation coefficient distributions in PBS/D_2O + 0.001% poloxamer-188 for AAV8-Lot1 to AAV8-Lot3 vector samples (see in Table S1).**

(A) Sedimentation coefficient distribution of AAV8-Lot1 vector sample, which is used as the standard for dFLISA analysis.

(B) Sedimentation coefficient distribution of AAV8-Lot2 vector sample, which is used as sample for dFLISA analysis.

(C) Sedimentation coefficient distribution of AAV8-Lot3 vector sample, which is used as sample for dFLISA analysis.

The observed peaks are assigned as empty particle (EP), full particle (FP), or extra filled (ExP). [1,2]

It is important to note that the unknown peak was not counted as particle.

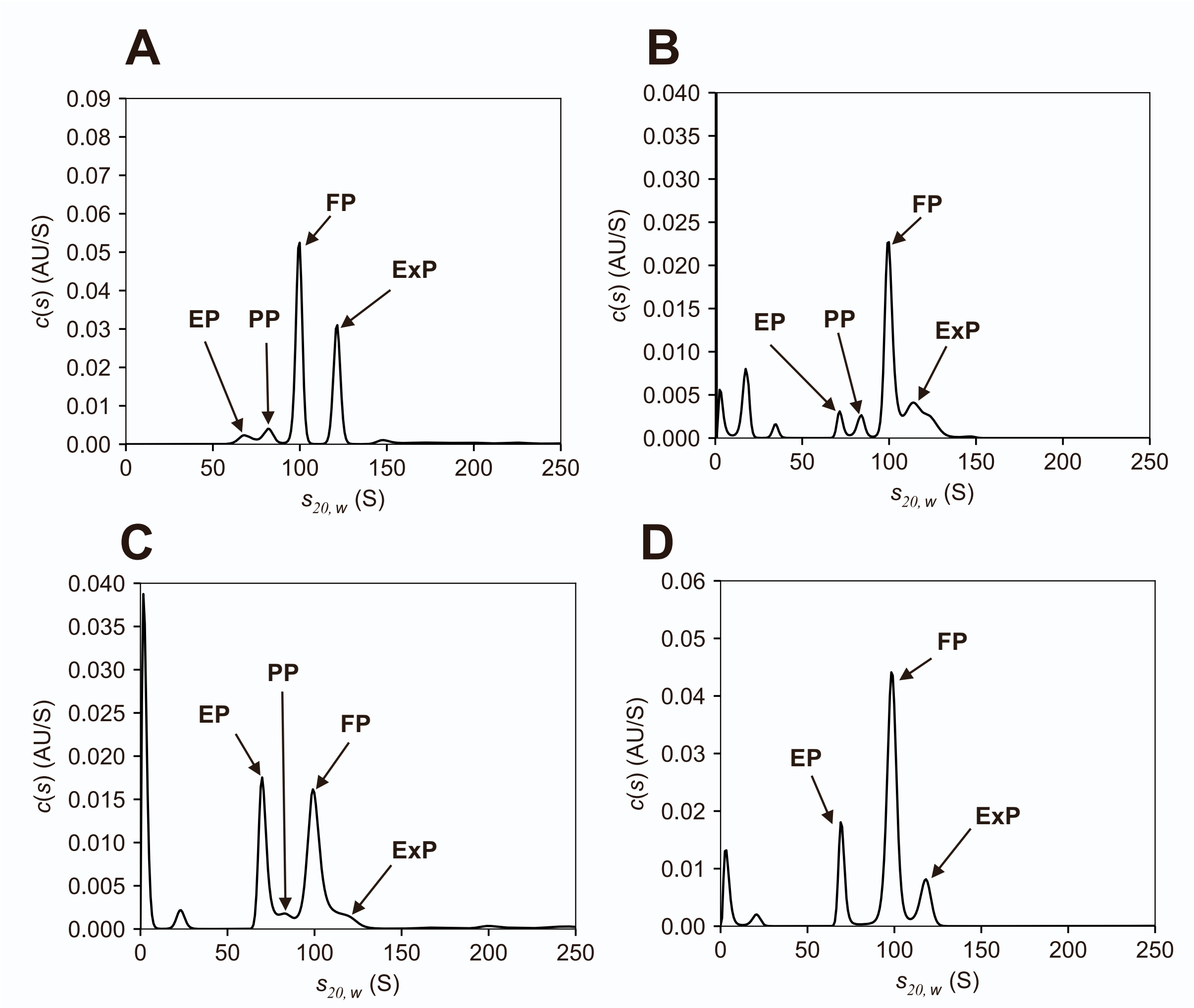

Figure S2. Representative sedimentation coefficient distributions in PBS/D_2O + 0.001% poloxamer-188 for AAV2-Lot3 to AAV2-Lot4 and AAV8-Lot5 to AAV8-Lot6 and vector samples (see in Table S2).

(A) Sedimentation coefficient distribution of AAV2-Lot3 vector sample, which is used as the Standard for dFLISA analysis.

(B) Sedimentation coefficient distribution of AAV2-Lot4 vector sample, which is used as the sample for dFLISA analysis.

(C) Sedimentation coefficient distribution of AAV8-Lot5 vector sample, which is used as the standard for dFLISA analysis.

(D) Sedimentation coefficient distribution of AAV8-Lot6 vector sample, which is used as the sample for dFLISA analysis.

The observed peaks are assigned as empty particle (EP), full particle (FP), extra filled particle (ExP) or partial particle (PP).

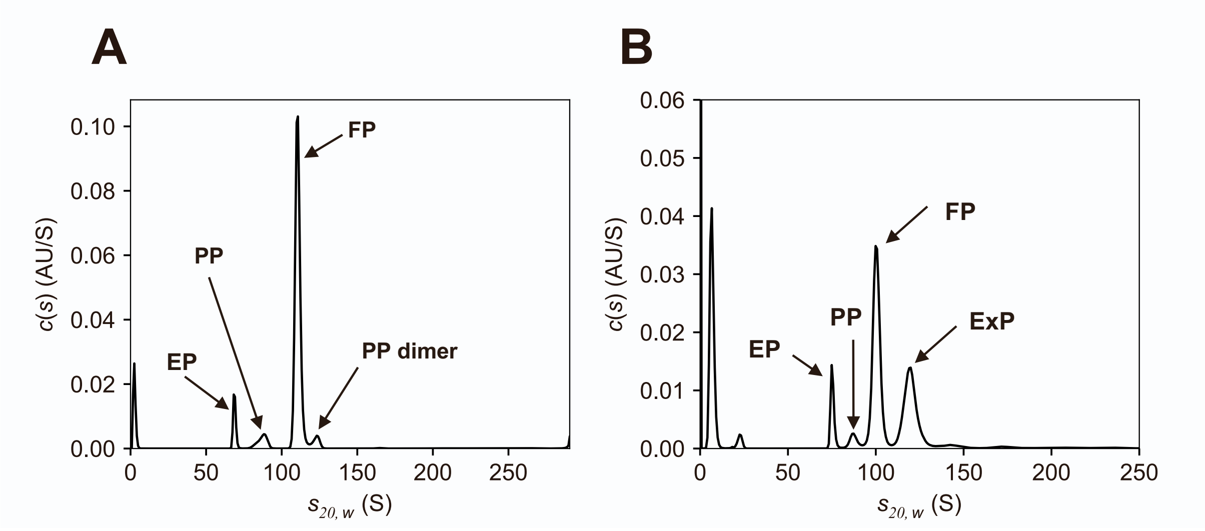

Figure S3. Representative sedimentation coefficient distributions in PBS/D_2O + 0.001% poloxamer-188 for AAV2-Lot1 to AAV2- Lot2 vector samples (see in Table S2).

(A) Sedimentation coefficient distribution of AAV2-Lot1 vector sample, which is used as the standard for dFLISA analysis. The observed peaks were identified as EP, PP, FP and PP dinner. PP is higher than LOQ, and the PP dimer is lower than LOQ of BS-AUC.

(B) Sedimentation coefficient distribution of AAV2-Lot2 vector sample, which is used as standard for dFLISA analysis. The observed peaks are assigned as empty particle (EP), full particle (FP); partial particle (PP) as shown in the figures.

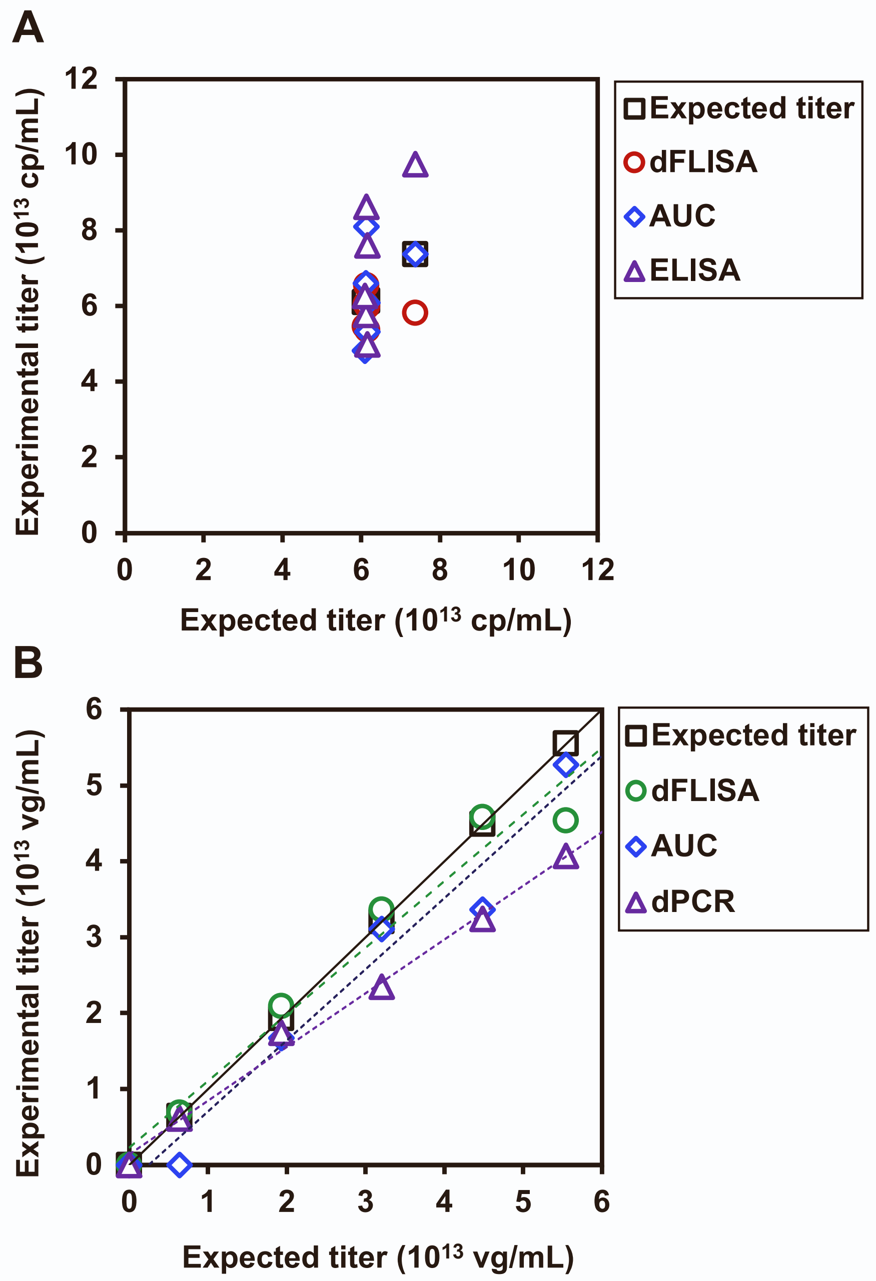

Figure S4. Linear correlation of total capsid titers (cp/mL) and genomic titer (vg/mL) of six mixed spike samples.

(A) Comparison of capsid titers (cp/mL) of six mixed spike samples analyzed by three techniques: dFLISA, BS-AUC, and ELISA. The capsid titer was adjusted to 6.16×10^{13} cp/mL for the mixed samples and to 6.09×10^{13} cp/mL for 0% FPs sample. The expected capsid titers (black square) were plotted on the horizontal axis, and the corresponding experimental capsid titer obtained by dFLISA

(green circle), BS-AUC (light blue rhombus) and ELISA (purple triangle) were plotted on the vertical axis.

(B) Comparison of genomic titers (vg/mL) of six mixed spike samples analyzed by three techniques: dFLISA, BS-AUC, and dPCR. The linear correlation of the expected genomic titers was plotted on the horizontal axis and the corresponding experimental genomic titer obtained by dFLISA (green circle), BS-AUC (light blue rhombus) and dPCR (purple triangle) were plotted on the vertical axis.

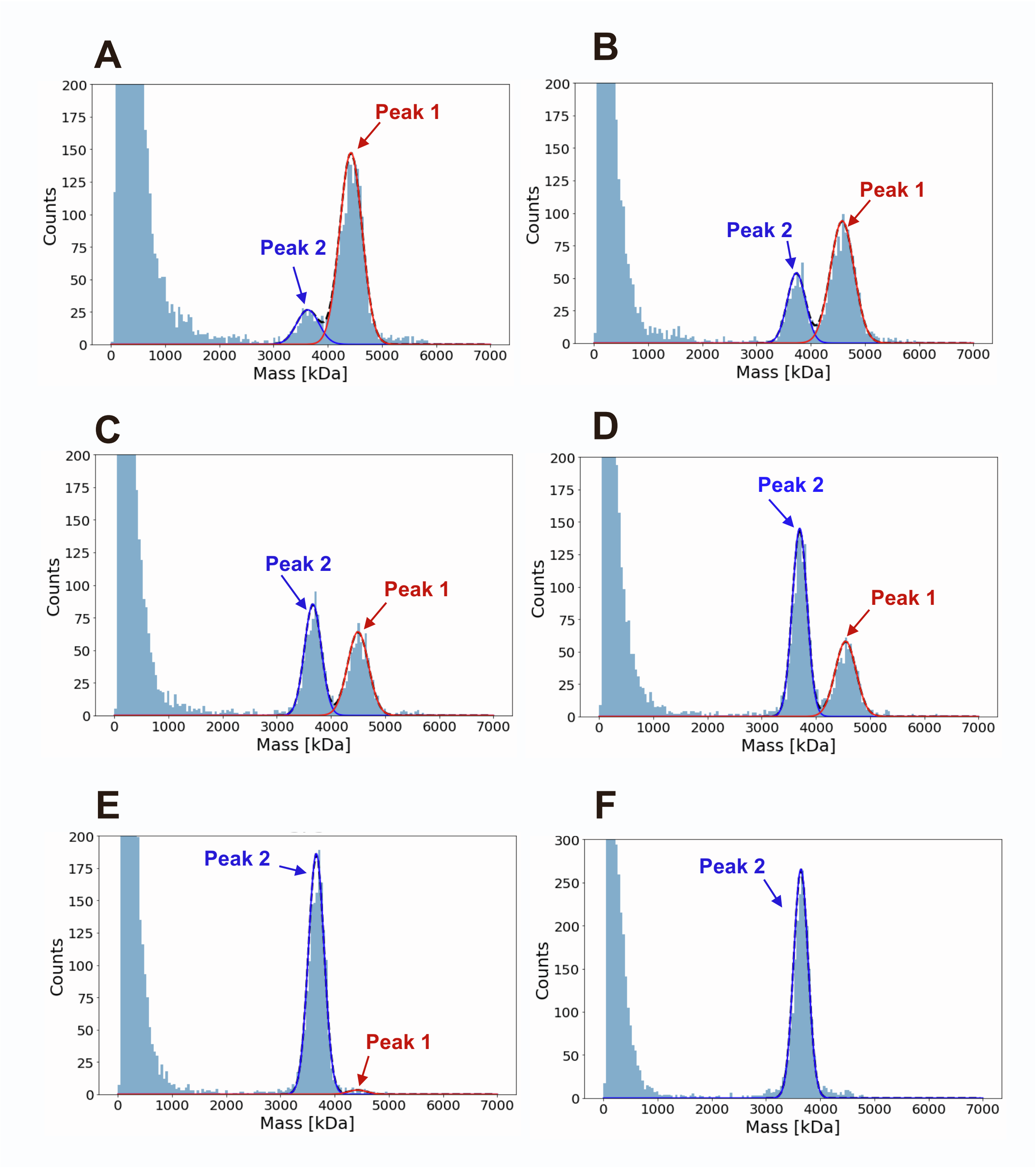

Figure S5. Histogram from MP analysis[3,5] of ssDNA packaged AAV8 capsids obtained from six mixed spike samples (90.1%, 73.1%, 52.3%, 31.5%, 10.5%, and 0% FPs).

(A) Constructed mass histograms of 90.1 % full capsid.

(B) Constructed mass histograms of 73.1 % full capsid.

(C) Constructed mass histograms of 52.3 % full capsid.

(D) Constructed mass histograms of 31.5 % full capsid.

(E) Constructed mass histograms of 10.5 % full capsid.

(F) Constructed mass histogram of 0 % full capsid.

Observed Peak1 (red) with mass corresponding to FP, while the observed Peak 2 (blue) with mass corresponding to EP. For each AAV8 sample, a single representative mass histogram is displayed. Gaussian distribution fit was applied to the histogram peaks. From these Gaussian fits, the percentage of full and empty AAV8 capsids were extracted.

EP, empty particle; FP; full particle; MP, mass photometry.

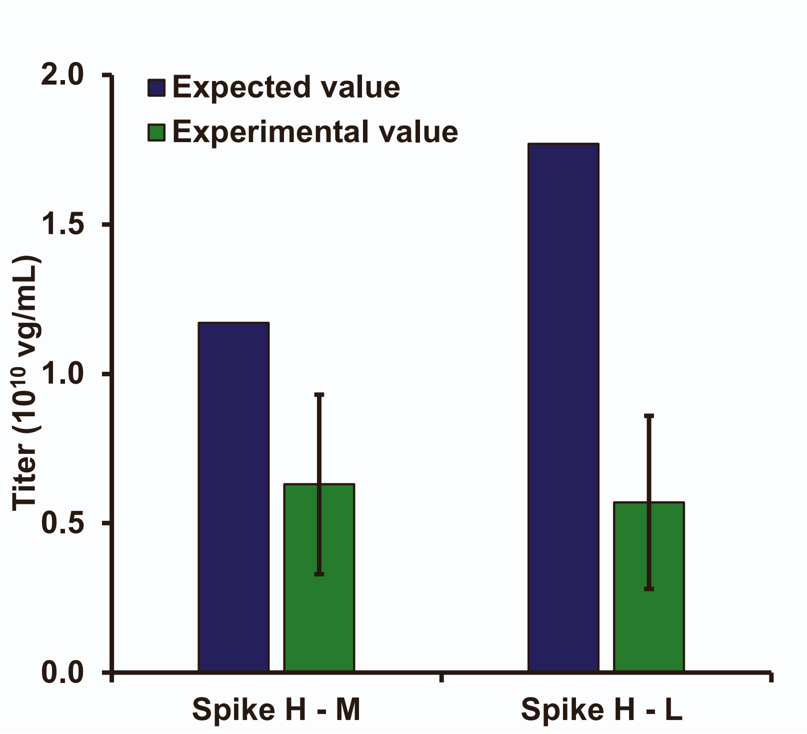

Figure S6. Spike-recovery test of dPCR.

Genomic titer quantification of crude samples by dPCR [7–9] were evaluated by spike-recovery test. The spike-recovery test was conducted as described in the method section: Quantification of crude sample by dFLISA and other method.

The different dilution factors for spike recovery were assessed by comparing the experimental values, as determined by the dPCR (dark green), with the expected values obtained by dFLISA (dark blue). The standard deviation (SD) of each parameter was obtained from the triplicated experiments.

H, high concentration spike; M, middle concentration spike; L, low concentration spike

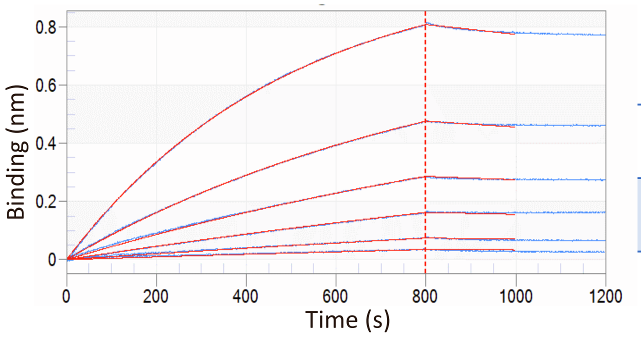

K_D (M)	k_{on} (1/Ms)	k_{off} (1/s)
2.73×10^{-11}	4.69×10^{6}	1.28×10^{-4}

Figure S7. Binding kinetics and affinity of AAV2 with anti-AAV VHH antibody measured by BLI

Biolayer Interferometry (BLI) measurements[4,6] were performed on Octet HTX system (Sartorius, Goettingen, Germany). Octet SAX biosensors (Sartorius) were hydrated by immersion in PBS for at least 10 min prior to use. Biotinylated anti-AAV VHH antibody (CaptureSelect™ Biotin Anti-AAVX Conjugate, Thermo Fisher Scientific) was diluted to 0.01 μg/mL and immobilized on the SAX biosensors. AAV2 samples were diluted in a 2-fold dilution series from 2.50×10^{11} vg/mL to 1.56×10^{10} vg/mL with Octet sample diluent (Sartorius). After the baseline measurement using Octet sample diluent, association and dissociation of AAV2 with the anti-AAV VHH antibody were measured at 30°C in an Octet 384-well tilted-bottom microplate (Sartorius) while shaking at 1000 rpm. Octet Analysis Studio software (ver. 12.2) was used for data analysis. The interaction between anti-AAV VHH antibody and AAV2 was analyzed by 1:1 fitting.

According to the manufacture's website of the 96-well plate, the maximum coating amount is 650 ng/cm^2. The bottom area of 96-well plates is approximately 0.33 cm^2, and the volume of AAV solution was 100 microliters. Thus, the coated concentration of VHH, whose molecular weight is 14 kDa, would be 150 nM. Using these values and the result of BLI, we calculated the binding efficiency of AAV based on the following formula:[10]

$$[\text{Complex}] = \frac{(K_D+[AAV]+[VHH])-\sqrt{(K_D+[AAV]+[VHH])^2-4[AAV][VHH]}}{2}$$

$$\text{Binding efficiency} = \frac{[Complex]}{[AAV]} \times 100$$

The binding efficiency of VHH antibody and AAV2 was >99% over the entire range of the standard curve. Although there is no information about the affinity of VHH for AAV8, the binding efficiency would be >98% even if K_D value of VHH for AAV8 is 100 times larger than that for AAV2.

Supplemental Tables

Table S1. Summary of in-house AAV8 vectors used in this study, as determined by BS-AUC

Sample name	Serotype	Promoter	GOI	Number of nucleic acids	FP (10^{13} vg/mL)	EP (10^{13} cp/mL)	ExP (10^{13} vg/mL)	PP (10^{13} vg/mL)	FP (%)	EP (%)	ExP (%)	PP (%)	FP+ExP (%)	FP+ExP (10^{13} vg/mL)	Total FP+ExP+EP (10^{13} cp/mL)	Sample preparation
AAV8-Lot1	8	CMV	EGFP	2521	1.09	0.11	0.24	<LOQ	76.10	8.23	15.67	na	91.8	1.31	1.43	Standard
AAV8-Lot2	8	HCRhAAT	FIX	2712	4.75	0.61	0.79	<LOQ	77.15	9.90	12.95	na	90.1	5.55	6.16	Sample
AAV8-Lot3	8	na	na	na	na	7.37	na	na	na	100	na	na	na	na	7.37	Sample

BS-AUC, band sedimentation analytical ultracentrifugation.
GOI, gene of interest; CMV, cytomegalovirus promoter; HCRhAAT, hepatic control region and human a1 antitrypsin promoter; EGFP, enhanced green fluorescent protein; FIX, factor IX.
cp, capsid particle; vg, viral genome; EP, empty particle; ExP, extra filled particle; FP, full particle; PP, partial particle; LOQ, Limit of quantification; na, not applicable.

Table S2. Summary of AAVs vectors purchased from VectorBuilder in this study, as determined by BS-AUC

Sample name	Serotype	Promoter	GOI	Number of nucleic acids	FP (10^{12} vg/mL)	EP (10^{12} cp/mL)	ExP (10^{12} vg/mL)	PP (10^{12} vg/mL)	FP (%)	EP (%)	ExP (%)	PP (%)	FP+ExP (%)	FP+ExP (10^{12} vg/mL)	Total FP+ExP+EP (10^{12} cp/mL)	Sample preparation
AAV2-Lot1	2	CMV	EGFP	2521	1.87	0.27	0.94	<LOQ	60.5	8.9	30.9	na	91.1	2.82	3.09	Sample
AAV2-Lot2	2	CMV	EGFP	3681	3.33	0.40	<LOQ	<LOQ	89.1	10.9	na	na	89.1	3.33	3.73	Sample
AAV2-Lot3	2	CMV	EGFP	2521	10.3	1.21	6.12	<LOQ	58.5	6.9	34.6	na	93.1	16.5	17.7	Standard
AAV2-Lot4	2	CMV	EGFP	2521	6.12	0.75	2.76	<LOQ	63.6	7.8	28.6	na	92.2	8.88	9.63	Sample
AAV8-Lot5	8	CMV	EGFP	2521	7.43	2.34	<LOQ	<LOQ	72.2	22.7	na	na	77.3	7.95	10.3	Standard
AAV8-Lot6	8	CMV	EGFP	2521	7.83	6.60	<LOQ	<LOQ	53.6	45.1	na	na	54.9	8.02	14.6	Sample

BS-AUC, band sedimentation analytical ultracentrifugation.
GOI, gene of interest.
CMV, cytomegalovirus promoter.
EGFP, enhanced green fluorescent protein.
cp, capsid particle; vg, viral genome EP, empty particle; ExP, extra filled particle; FP, full particle; LOQ, Limit of quantification; PP, partial particle; na, not applicable.

Table S3. Precision and accuracy of the dFLISA of capsid titer (cp/mL)

Sample	Expected value	Experimental value				SD (10^{10} cp/mL)	CV (%)	Accuracy (%)
	Concentration (10^{10} cp/mL)	Concentration (10^{10} cp/mL)						
	Concentration	Day 1	Day 2	Day 3	Average titer			
1	15.4	15.7	15.9	16.1	15.9	0.21	1.3	102.8
2	7.70	6.98	6.48	6.45	6.64	0.29	4.4	87.6
3	3.85	3.50	3.31	3.23	3.35	0.14	4.2	87.6
4	1.93	1.81	1.53	1.50	1.61	0.17	10.6	84.1
5	0.96	0.84	0.67	0.76	0.76	0.08	10.8	76.0
6	0.48	0.29	0.39	0.35	0.34	0.04	14.4	74.3
7	0.24	0	0	0.16	0	0	0	0

Results represent the mean values from 3-day experiments, in which each sample was analyzed in duplicate wells.
The samples were initially diluted 400-fold, followed by a 2-fold serial dilution.
CV, coefficient of variation; cp, capsid particle; SD, standard deviation.

Table S4. Precision and accuracy of the dFLISA of genomic titer (vg/mL)

Sample	Expected value	Experimental value				Full capsid ratio (%)	SD (10^{10} vg/mL)	CV (%)	Accuracy (%)
	Concentration (10^{10} vg/mL)	Concentration (10^{10} vg/mL)							
	Concentration	Day 1	Day 2	Day 3	Average titer				
1	13.9	13.7	14.0	13.3	13.7	85.8	0.31	2.3	98.4
2	6.94	6.60	6.58	6.37	6.52	98.2	0.12	1.9	93.9
3	3.47	3.27	3.10	2.89	3.09	92.2	0.18	6.1	89.0
4	1.73	1.80	1.47	1.14	1.47	91.3	0.33	22.6	84.7
5	0.86	0.69	0.35	0.31	0.45	60.1	0.21	46.1	52.7
6	0.43	0	0	0	0	0	0	0	0
7	0.21	0	0.54	0	0	0	0	0	0

Results represent the mean values from 3-day experiments, in which each sample was analyzed in duplicate wells.
The samples were initially diluted 400-fold, followed by a 2-fold serial dilution.
CV, coefficient of variation; SD, standard deviation; vg, viral genome.

Table S5. Determination LOQ of dFLISA for capsid titer detection

Blank Intensity (10^4)					Concentration calculated from blank intensity + 10 SD (10^{10} cp/mL)
Day 1	Day 2	Day 3	Average intensity	SD	
1.65	2.91	2.05	2.20	0.29	0.60

The limit of quantification (LOQ) of capsid quantification are estimated from fluorescence intensities of blank.
Results represent the mean results of 3-day experiments in which each sample was analyzed in duplicate wells.
cp, capsid particle; SD, standard deviation.

Table S6. Determination LOQ of dFLISA for genomic titer detection

Blank Intensity (10^4)					Concentration calculated from blank intensity + 10 SD (10^{10} vg/mL)
Day 1	Day 2	Day 3	Average intensity	SD	
10.0	9.10	9.20	9.56	1.27	1.70

The limit of quantification (LOQ) of genome quantification are estimated from fluorescence intensities of blank.
Results represent the mean results of 3-day experiments in which each sample was analyzed in duplicate wells.
SD, standard deviation.
vg, viral genome.

Table S7. Comparison of capsid titers (cp/mL) of six mixed spike samples using orthogonal techniques: dFLISA, BS-AUC, and ELISA

Sample	Expected value	Experimental value		
	AUC (10^{13} cp/mL)	dFLISA (10^{13} cp/mL)	AUC (10^{13} cp/mL)	ELISA (10^{13} cp/mL)
90.1% full	6.16	6.36	6.09	7.60
73.1% full	6.15	7.10	5.32	4.99
52.3% full	6.13	7.77	8.11	8.63
31.5% full	6.12	7.17	6.60	5.77
10.5% full	6.10	6.46	4.82	6.26
0% full	7.37	5.71	7.37	9.75

AUC, analytical ultracentrifugation

dFLISA, dual fluorescence-linked immunosorbent assay.

ELISA, enzyme-linked immunosorbent assay.

cp, capsid particle.

Table S8. Comparison of genomic titers (vg/mL) of six mixed spike samples using orthogonal techniques: dFLISA, BS-AUC, and dPCR

Sample	Expected value	Experimental value		
	AUC (10^{13} vg/mL)	dFLISA (10^{13} vg/mL)	AUC (10^{13} vg/mL)	dPCR (10^{13} vg/mL)
90.1% full	5.55	5.46	5.27	4.07
73.1% full	4.49	5.51	3.36	3.24
52.3% full	3.21	4.04	3.11	2.35
31.5% full	1.93	2.51	1.67	1.74
10.5% full	0.64	8.39	nd[a]	0.61
0% full	0	0	0	0

AUC, analytical ultracentrifugation

dFLISA, dual fluorescence-linked immunosorbent assay.

dPCR, digital chain polymerase reaction.

[a]nd, not detected; vg, viral genome.

Table S9. Comparison of fluorescence intensity of AAV2 with different genome lengths by dFLISA

Genomic titer		Fluorescence intensity[c]			
(10^{10} vg/mL)	scDNA[a] (10^5)	ssDNA[b] (10^5)	Ratio (scDNA/ssDNA)	Average value	SD
1.41	11.11	5.93	1.87		
0.70	5.95	3.22	1.85	1.86	0.015
0.35	3.49	1.89	1.85		

[a]scDNA (3681 base), self-complementary DNA.

[b]ssDNA (2521 base), single-stranded DNA.

[c]Calculated from standard curve of AAV vectors with scDNA and ssDNA.

SD, standard deviation.

References

1. Maruno, T., Ishii, K., Torisu, T., and Uchiyama, S. (2023). Size Distribution Analysis of the Adeno-Associated Virus Vector by the c(s) Analysis of Band Sedimentation Analytical Ultracentrifugation with Multiwavelength Detection. J Pharm Sci *112*, 937–946. https://doi.org/10.1016/j.xphs.2022.10.023.
2. Hirohata, K., Yamaguchi, Y., Maruno, T., Shibuya, R., Torisu, T., Onishi, T., Chono, H., Mineno, J., Yuzhe, Y., Higashiyama, K., et al. (2024). Applications and Limitations of Equilibrium Density Gradient Analytical Ultracentrifugation for the Quantitative Characterization of Adeno-Associated Virus Vectors. Anal Chem *96*, 642–651. https://doi.org/10.1021/acs.analchem.3c01955.
3. Hiemenz, C., Baumeister, N., Helbig, C., Hawe, A., Babutzka, S., Michalakis, S., Friess, W., and Menzen, T. (2023). Genome length determination in adeno-associated virus vectors with mass photometry. Mol Ther Methods Clin Dev *31*. https://doi.org/10.1016/j.omtm.2023.101162.
4. Meierrieks, F., Kour, A., Pätz, M., Pflanz, K., Wolff, M.W., and Pickl, A. (2023). Unveiling the secrets of adeno-associated virus: novel high-throughput approaches for the quantification of multiple serotypes. Mol Ther Methods Clin Dev *31*. https://doi.org/10.1016/j.omtm.2023.101118.
5. Wagner, C., Fuchsberger, F.F., Innthaler, B., Lemmerer, M., and Birner-Gruenberger, R. (2023). Quantification of Empty, Partially Filled and Full Adeno-Associated Virus Vectors Using Mass Photometry. Int J Mol Sci *24*. https://doi.org/10.3390/ijms241311033.
6. Fu, Y., Choudhary, D., Liu, N., Moon, Y., Abdubek, P., Sweezy, L., Rosconi, M., Palackal, N., and Pyles, E. (2023). Comprehensive biophysical characterization of AAV-AAVR interaction uncovers serotype- and pH-dependent interaction. J Pharm Biomed Anal *234*. https://doi.org/10.1016/j.jpba.2023.115562.
7. Shmidt, A.A., and Egorova, T. V. (2022). PCR-based analytical methods for quantification and quality control of recombinant adeno-associated viral vector preparations. Preprint at MDPI, https://doi.org/10.3390/ph15010023 https://doi.org/10.3390/ph15010023.
8. Dobnik, D., Kogovšek, P., Jakomin, T., Košir, N., Žnidarič, M.T., Leskovec, M., Kaminsky, S.M., Mostrom, J., Lee, H., and Ravnikar, M. (2019). Accurate quantification and characterization of adeno-associated viral vectors. Front Microbiol *10*. https://doi.org/10.3389/fmicb.2019.01570.
9. Kojabad, A.A., Farzanehpour, M., Galeh, H.E.G., Dorostkar, R., Jafarpour, A., Bolandian, M., and Nodooshan, M.M. (2021). Droplet digital PCR of viral DNA/RNA, current progress, challenges, and future perspectives. Preprint at John Wiley and Sons Inc, https://doi.org/10.1002/jmv.26846 https://doi.org/10.1002/jmv.26846.
10. Marsh Editor, J.A. Protein Complex Assembly Methods and Protocols Methods in Molecular Biology 1764.
