# Supplementary material for: Quantification of full and empty particles of adeno-associated virus vectors via a novel dual fluorescence-linked immunosorbent assay
Source: Mol Ther Methods Clin Dev. 2024 Jun 24;32(3):101291. doi: 10.1016/j.omtm.2024.101291 (PMC11283060; doi:10.1016/j.omtm.2024.101291)
Supplement: Document S2. Article plus supplemental information [file mmc2.pdf]

Molecular Therapy
Methods & Clinical Development
Original Article

Quantification of full and empty particles of adeno-associated virus vectors via a novel dual fluorescence-linked immunosorbent assay

Sereirath Soth,[1] Mikako Takakura,[1] Masahiro Suekawa,[1] Takayuki Onishi,[1] Kiichi Hirohata,[1] Tamami Hashimoto,[1] Takahiro Maruno,[1] Mitsuko Fukuhara,[1] Yasuo Tsunaka,[1] Tetsuo Torisu,[1] and Susumu Uchiyama[1,2]

[1]Department of Biotechnology, Graduate School of Engineering, Osaka University, 2-1 Yamadaoka, Suita, Osaka 565-0871, Japan; [2]Exploratory Research Center on Life and Living Systems, National Institutes of Natural Sciences, 5-1 Higashiyama, Myodaiji, Okazaki, Aichi 444-8787, Japan

The adeno-associated virus (AAV) vector is one of the most advanced platforms for gene therapy because of its low immunogenicity and non-pathogenicity. The concentrations of both AAV vector empty particles, which do not contain DNA and do not show any efficacy, and AAV vector full particles (FPs), which contain DNA, are important quality attributes. In this study, a dual fluorescence-linked immunosorbent assay (dFLISA), which uses two fluorescent dyes to quantify capsid and genome titers in a single analysis, was established. In dFLISA, capture of AAV particles, detection of capsid proteins, and release and detection of the viral genome are performed in the same well. We demonstrated that the capsid and genomic titers determined by dFLISA were comparable with those of analytical ultracentrifugation. The FP ratios determined by dFLISA were in good agreement with the expected values. In addition, we showed that dFLISA can quantify the genomic and capsid titers of crude samples. dFLISA can be easily modified for measuring other AAV vector serotypes and AAV vectors with different genome lengths. These features make dFLISA a valuable tool for the future development of AAV-based gene therapies.

INTRODUCTION

Recombinant adeno-associated virus (rAAV) vectors have become highly effective tools in human gene therapy, primarily because of the exceptional properties of AAV[1,2]; its non-pathogenicity, ability to infect multiple cell types, and maintenance of the viral genome within host cells have enabled clinical successes in the treatment of genetic and acquired diseases.[3–7] Another advantage of AAV vectors is that there are several serotypes, each with different tissue tropisms.[8–10] Despite the advantages of using AAVs for therapeutic purposes, there are several challenges to be overcome. Empty AAV vector particles (EPs), partial particles (PPs), which lack therapeutic deoxyribonucleic acid (DNA) or only contain fragments of the genome,[11,12] and extra filled particles (ExPs), which contains higher numbers of DNA, are generated in upstream production processes, and complete removal of the particles in downstream processes is impractical because of their physicochemical similarity to full particles (FPs), which contain therapeutic DNA.[13–16] EPs and PPs are considered impurities that potentially trigger adverse immunogenic reactions.[11,17–19] Furthermore, these impurities may compete with FPs for binding to target cell receptors, potentially reducing their therapeutic efficacy.[20–22] The clinical impact of EPs is not fully understood, but they are recognized to be significant obstacles affecting FPs biodistribution and potentially provoking immune responses. Therefore, analytical techniques to evaluate the purity of AAVs are crucial for AAV vector development.[22] Some analytical methods are available to assess the contents of FPs and EPs. Combination of genome quantitation by quantitative polymerase chain reaction (qPCR) and capsid quantitation by enzyme-linked immunosorbent assay (ELISA) has been conventionally employed for the estimation of FP ratio. Now, band sedimentation analytical ultracentrifugation (BS-AUC) is recognized as the gold standard for the size distribution analysis of AAV vectors and can quantify FPs, EPs, PPs, and ExPs with high precision.[23–25] Charge detection mass spectrometry and transmission electron microscopy could be orthogonal methods for the size distribution analysis. They are able to quantify FP ratio[26–30]; and furthermore could provide aggregation, fragmentation, and mass distribution of packaged DNA.[31–33] Mass photometry (MP) is a method that measures the mass of individual particles and provides the percentage of each kind of particle against total counts (% counts).[34,35] However, these analytical methods have limitations, especially in the case of crude samples. For example, it is burdensome that prior purification is required before using these analyses. A combination of ELISA and qPCR,[36–38] which do not require purification before analysis, has been used to quantify capsid and genome titers, respectively, and to calculate FP ratios. Besides qPCR, digital PCR (dPCR), or digital droplet PCR are used for detecting the genomic titer of the viral vector.[39–43]

Received 4 January 2024; accepted 21 June 2024;
https://doi.org/10.1016/j.omtm.2024.101291.

Correspondence: Tetsuo Torisu, Department of Biotechnology, Graduate School of Engineering, Osaka University, 2-1 Yamadaoka, Suita, Osaka 565-0871, Japan.
E-mail: tetsuo.torisu@bio.eng.osaka-u.ac.jp
Correspondence: Susumu Uchiyama, Department of Biotechnology, Graduate School of Engineering, Osaka University, 2-1 Yamadaoka, Suita, Osaka 565-0871, Japan.
E-mail: suchi@bio.eng.osaka-u.ac.jp

Published by Elsevier Inc. on behalf of The American Society of Gene and Cell Therapy.
This is an open access article under the CC BY license (http://creativecommons.org/licenses/by/4.0/).

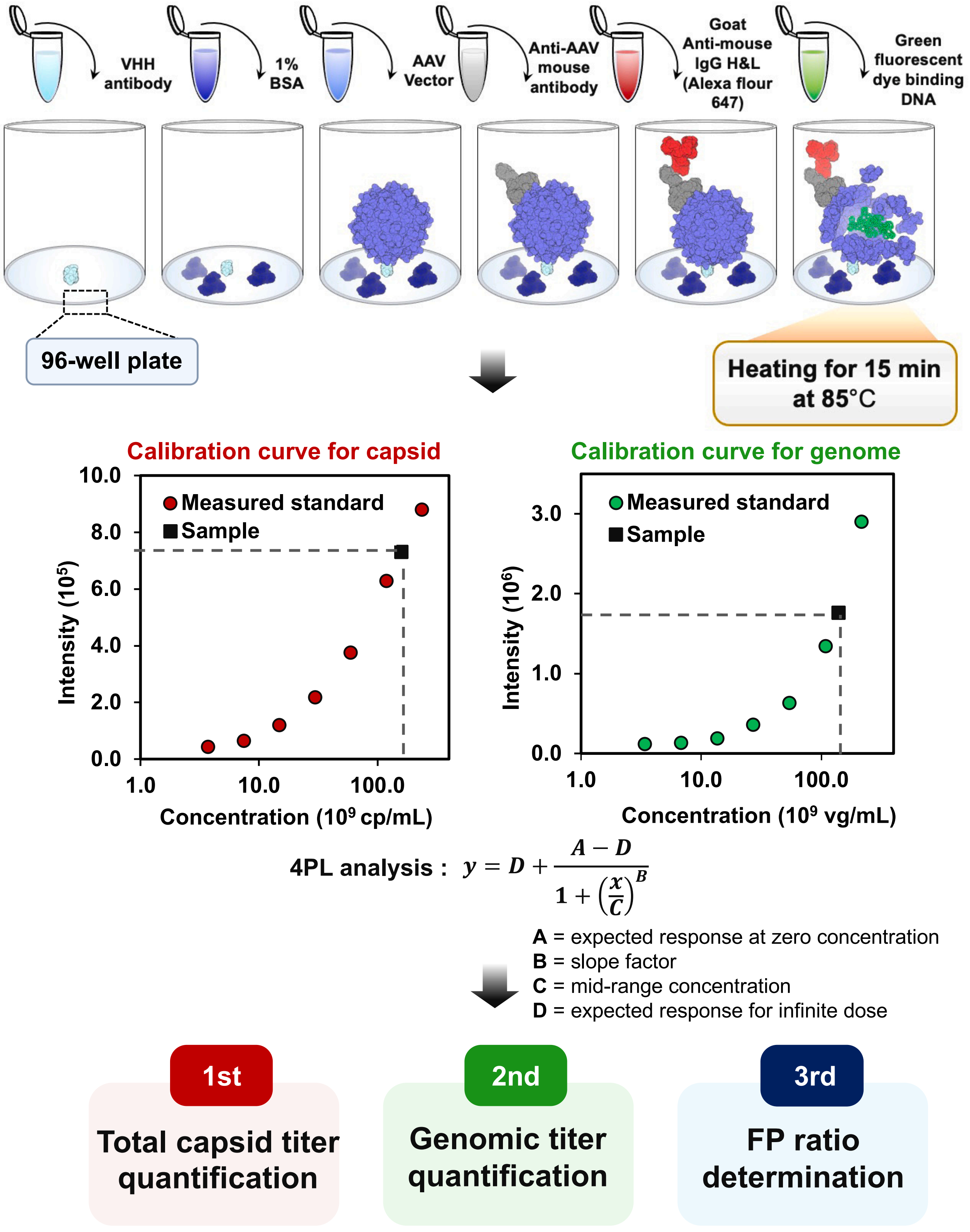

(legend on next page)

However, the combination of ELISA and PCR is subject to inherent drawbacks of error and variability[44–48] because it relies on data from two independent quantitative analyses, which are based on different principles, and capsid and genomic titers must be quantified separately using non-identical samples.[49–54]

In our study, we aimed to establish a dual fluorescence-linked immunosorbent assay (dFLISA) as an analytical method capable of simultaneously quantifying viral capsid and genomic titers in a single analysis (Figure 1). This method is primarily based on ELISA,[53,55] followed by genome staining where two different fluorescent dyes are employed to quantify full and empty AAV vector particles and the FP ratio. After the addition of a secondary antibody conjugated to one fluorescent dye, the plate is subjected to heat treatment to release the genome from the capsid before the introduction of the second fluorescent dye. dFLISA allows the determination of the FP ratio in a simple way with high precision, high accuracy, and high sensitivity. The capsid and genomic titers that give FP ratio in crude samples, which contain impurities, including host cell DNA and proteins, were also successfully obtained by dFLISA.

RESULTS

dFLISA

A 96-well plate was first coated with anti-AAV VHH antibody, followed by the addition of bovine serum albumin (BSA) for blocking. Standards and samples were then added to the wells. The binding efficiencies of the anti-AAV VHH antibody for AAV2 and AAV8 were estimated as >98% (Figure S7). Mouse anti-AAV antibody was added after removal of unbound AAVs. Then goat anti-mouse antibody conjugated to Alexa Fluor 647 was used to detect and quantify the AAV capsid proteins. The plate was washed to remove excess goat antibody and heated at 85°C for 15 min to disrupt the capsid and release the genome. SYBR gold solution was then added to each well to detect DNA.[56] Because SYBR gold is fluorescent only when it is bound to DNA,[42] genomes can be quantified even when they are no longer immobilized on the plate and without washing out the unbound SYBR gold dye. Standard curves were generated using four-parameter logistic regression to calculate the capsid and genome titers. The FP ratio was calculated from these values—capsid and genome titers are considered to represent total and FP concentrations, respectively (Figure 1).

Quantification of capsid and genomic titers by dFLISA

The precision and accuracy of capsid and genomic titer quantification by dFLISA were evaluated by analyzing purified AAV8 samples on three separate occasions over three consecutive days (Figures 2A–2C). The capsid concentration of the original sample solution was determined in advance as 1.54×10^{11} capsid protein (cp)/mL by BS-AUC. These samples were serially diluted at a 1:2 ratio, resulting in the series of dilutions shown in Tables S3 and S4. For precision, the coefficient of variation (%CV) of the capsid titer was less than 15% for all samples and less than 11% for samples 1–4 (Table S3). The %CV of genomic titer quantification was less than 7% for samples 1–3, and the %CV of the genomic titer of sample 4 was 22.6%. Accuracy was evaluated based on the ratio of experimental/expected values. The ratios of samples 1–4 were consistently within the range 80%–100% for both capsid and genomic titers. The ratios of experimental/expected values of samples 5–7 were lower than 80% (Table S4).

Determination of FP ratio via dFLISA

We investigated the linearity of the FP ratio calculated by dFLISA. Samples with different FP ratios (0%, 10.5%, 31.5%, 52.3%, 73.1%, and 90.1% of FPs) were prepared by mixing two samples: AAV8-Lot2 (FP ratio 90.1%) and AAV8-Lot3 (FP ratio 0%). Excellent correlation and linearity in the FP ratio were observed, with an R^2 value of >0.99, and the slope of the plot against the expected values was 0.97. In addition, the %CV of the FP ratio was less than 25%. These results indicate that dFLISA has sufficient precision, accuracy, and linearity for FP ratio determination (Figure 3).

According to the criteria for accuracy and precision described in the materials and methods, the concentration of samples 1–3 should be within the quantification range of dFLISA, and sample 4 (1.61×10^{10} cp/mL, 1.47×10^{10} vector genome (vg)/mL) met the criteria for the limit of quantification (LOQ). In addition, the concentration was calculated from fluorescence intensities of blank +10 SD, which is also used to determine LOQ. The capsid titer for sample 4 was higher than that of blank intensity +10 SD (Tables S3 and S5), while the genome titer for sample 4 was lower than that of blank intensity +10 SD (Tables S4 and S6). The higher values were determined as the LOQ of dFLISA for capsid and genome titer quantification: 1.61×10^{10} cp/mL for capsid titer and 1.70×10^{10} vg/mL for genomic titer.

Comparison of genomic titer by orthogonal methods using mixed samples

We evaluated the suitability of dFLISA for vector analysis by comparing it with various particle-measuring techniques, including BS-AUC, MP, and the combined dPCR/ELISA method (Tables S7 and S8).[47,54,57] As shown in Figure 3, dFLISA showed good correlation with the orthogonal determination of the FP ratio. The results of the MP were in good agreement with that of dFLISA, except for

Figure 1. Schematic illustration of dFLISA analysis

The soluble biotinylated anti-AAVX conjugate VHH affinity ligand, which exhibits high affinity for AAVX, was immobilized directly onto a black 96-well plate and used as a capture protein. Subsequently, 1% BSA was added, and the individual wells were loaded with vector stocks comprising a variety of AAV samples. A mouse monoclonal antibody targeting intact AAV particles was used as the primary antibody against AAV. To enable detection, we used a goat anti-mouse IgG H&L-labeled secondary antibody (Alexa Fluor 647). The viral capsid was disrupted, and ssDNA was released by the addition of 1× PBS to each well, followed by incubation at 85°C for 15 min. Next, we added diluted SYBR gold solution to each well and incubated the plate at room temperature for 5 min. This technique allowed us to generate the calibration curve and thus measure both red and green fluorescence, providing an assessment of the capsid and genomic titers, as well as the FP ratio, through simultaneous dual-wavelength measurements.

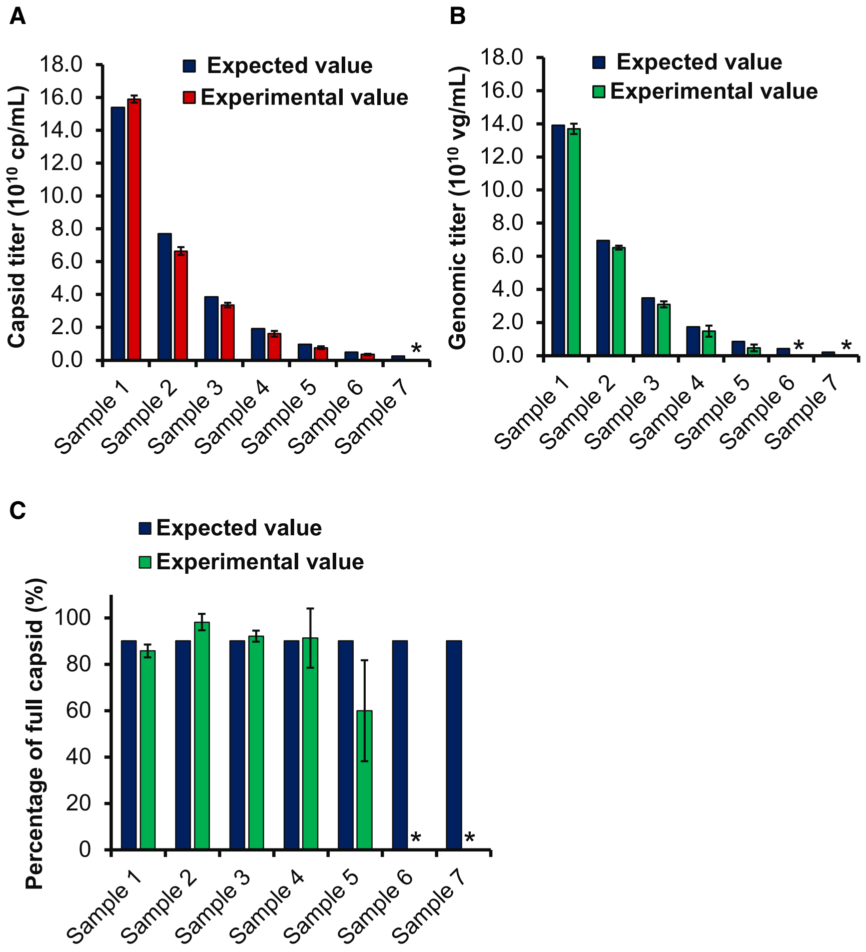

Figure 2. Quantification of capsid and genomic titers by dFLISA

(A) Capsid titer quantification. (B) Genomic titer quantification. (C) Percentage of full capsids. In this quantitative analysis, two different samples of Serotype 8, AAV8-Lot1 and AAV8-Lot2, were used. Each sample was initially formulated in PBS, 200 mM NaCl, and 0.001% poloxamer-188. AAV8-Lot1 was used to establish the reliability of the calibration curve and had a concentration of 1.43×10^{13} cp/mL and 1.31×10^{13} vg/mL, and then diluted 60-fold with 0.05% Tween 20 in 1× PBS. AAV8-Lot2 was used as an unknown sample and had a concentration of 6.16×10^{13} cp/mL and 5.55×10^{13} vg/mL, and then diluted 400-fold with 0.05% Tween 20 in 1× PBS. Serial 2-fold dilutions were performed daily to obtain seven samples while avoiding freeze-thaw cycle and maintaining consistent operating conditions for a brief time and both samples were prepared without undergoing freeze-thaw cycles. The obtained responses were plotted using dFLISA data (experimental value) and BS-AUC data (expected value). The mean values from experiments conducted over 3 days are presented in the results. Each sample was analyzed in duplicate wells, and error bars indicate the SD within each sample. Asterisks (*) are used to indicate cp/mL and vg/mL values that were below the limit of quantification.

a 10% FP sample. BS-AUC showed lower FP values than expected, and the dPCR/ELISA results were in close agreement with the expected values of 73.1%, 31.5%, and 10.5% FP. However, the dPCR/ELISA results for the 90.1% and 52.3% FP samples were very different because of variations in the PCR results.

Applicability of dFLISA for crude samples

The purified sample, AAV8-Lot2, was first concentrated to final concentrations of 6.16×10^{13} cp/mL and 5.55×10^{13} vg/mL, and then diluted to three different concentrations: high (spike H) at 1.23×10^{11} cp/mL and 1.11×10^{11} vg/mL, medium (spike M) at 0.82×10^{11} cp/mL and 0.74×10^{11} vg/mL, and low (spike L) at 0.61×10^{11} cp/mL and 0.55×10^{11} vg/mL. The purified samples and crude lysate were mixed at a 1:1 ratio. The recovery of the spiked purified sample was evaluated as the difference between the high minus the middle (spike H − M) and the high minus the low (spike H − L) concentrations because the crude lysate contained an unknown amount of AAV particles. Recovered capsid titers of the spike H − M and spike H − L were 1.25×10^{10} cp/mL and 2.06×10^{10} cp/mL, respectively (Figure 4A), and the recovered genomic titers were spike H − M and spike H − L of 0.91×10^{10} vg/mL and 1.61×10^{10} vg/mL, respectively (Figure 4B). The FP ratios of spike H − M and spike H − L were 73.0% and 77.9%, respectively (Figure 4C). Spike recovery was consistently achieved across all mixed samples. Specifically, the capsid titer recovery rate was 122.1% ± 133.8%, and the genome recovery rate was 98.8% ± 115.7%. The obtained results met the criteria written in the materials and methods. The results suggest that the impurities in crude lysate do not interfere with capsid/genome quantification by dFLISA. dFLISA was then applied to the quantification of capsid and genomic titers of a crude sample. A dFLISA analysis of crude AAV yielded capsid and genome titers of 3.28×10^{12} cp/mL and 7.77×10^{11} vg/mL, respectively (Figure 5). There was no significant difference in capsid titer between dFLISA and ELISA (p = 0.303), which is a well-established method.[7,55] In the genomic titer measurements, dFLISA showed significantly higher titers than dPCR (p = 0.029) (Figure 5).

Application of dFLISA for quantifying different AAV serotypes

Because VHH has been shown to bind to a broader range of serotypes (specifically, AAV1 to AAV8 and AAVrh10),[58–61] which is sufficient for the needs of current clinical research,[62] we further extended the applicability of the dFLISA method to facilitate the quantification of AAV of different serotypes. In this approach, the primary antibody was replaced by an antibody that specifically targets the serotype of interest. The capsid and genomic titers of AAV2-Lot4 were quantified by dFLISA using AAV2-Lot3 as a reference standard of AAV2-containing ssDNA. Figure 6 shows that the experimental values were

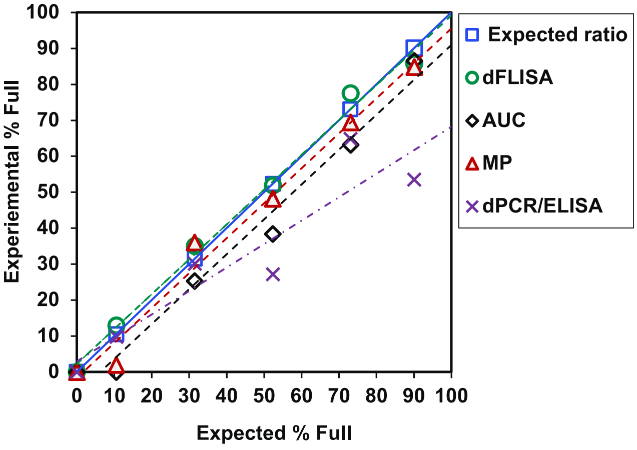

Figure 3. Determination of FP ratio by dFLISA and orthogonal method
For the determination of the full-to-empty capsid ratio by dFLISA, capsid and genomic titer quantification was repeated on three consecutive days (days 1–3) by mixing two AAV8 samples, full and empty titers, 6.16 × 10^{13} cp/mL and 7.37 × 10^{13} cp/mL, respectively, to obtain different FP ratios ranging from 0% to 90.1% FPs. A good linear correlation was obtained between the dFLISA data (experimental % full) shown on the vertical axis and the BS-AUC data (expected % full) shown on the horizontal axis. Results are the means of 3-day experiments in which each sample was analyzed in duplicate wells, with error bars corresponding to the standard deviation (SD) of each population. As a comparison of genomic titer by orthogonal methods using mixed samples, the graph shows the relationship between the expected percentage of full capsids as determined by BS-AUC (blue square) on the vertical axis and the corresponding experimental percentage of full capsids on the horizontal axis. The graph includes data points representing experimental results obtained by dFLISA (green circle) and different orthogonal methods, specifically BS-AUC (black rhombus), MP (red triangle), and dPCR/ELISA (purple multiplication sign). The average of duplicate wells are shown, and the error bars indicate the SD within each sample.

consistent and comparable with the expected values (±25% of expected value). This suggests that the modified dFLISA method is effective in quantifying AAVs of different serotypes.

Fluorescence intensity of SYBR gold with different genome lengths

We investigated the correlation between different AAV vector genome lengths and SYBR gold fluorescence intensity. First, a DNA mixture was analyzed by gel electrophoresis and dyed with SYBR gold. The intensity of each band was normalized to a relative concentration determined by MP and plotted against DNA length. As shown in Figure 7, the fluorescence intensity correlated well with genome length. AAV2 capsids containing different genome types, specifically self-complementary DNA (scDNA) (3,681 bases) and single-stranded DNA (ssDNA) (2,521 bases), were analyzed by dFLISA, and their fluorescence intensities were compared (Table S9). The fluorescence intensity of the ssDNA AAV2 vector was approximately 1.86 times lower than that of the scDNA AAV2 vector (Table S9), while the fluorescence intensity of ssDNA estimated from the curve (Figure 7) was approximately 1.4 times lower than that of scDNA.

DISCUSSION

This study aimed to establish a simple and reliable method of measuring AAV vector titers and the ratio of FP. We developed dFLISA, which uses two fluorescent dyes to quantify capsid and genome titers.[63–65] The precision, accuracy, and quantification limits of dFLISA were assessed. In addition, the applicability of dFLISA for different detecting AAV vector serotypes and AAV vectors with different genome lengths were evaluated.

Precision and accuracy of dFLISA analysis

The dFLISA method consistently yielded precision values below 15% across all tested samples while maintaining an accuracy of 80%–100% of the expected values for both capsid and genomic titers in samples 1–4, except in cases where the values approached or fell below the LOQ, as with samples 5–7 (Figure 2; Tables S3 and S4). This indicated the good precision and accuracy of our approach for obtaining capsid and genome titers, not only outperforming the combined dPCR and ELISA but also showing a significant improvement in error minimization. The relative concentrations of ExPs were relatively similar between the standard (AAV8-Lot1, 15.67%) and the sample (AAV8-Lot2, 12.95%) (Table S1). Because the genomic titer determined by dFLISA was the sum of FPs and ExPs, a difference in the relative concentrations of FPs and ExPs between standards and samples could result in inaccuracy and imprecision. Although the PP concentrations of samples used were lower than the LOQ of BS-AUC[63] and were ignored in this study, influence of PPs on the capsid and genomic titers should be considered carefully. dFLISA can simply quantify capsid and genomic titers; however, the inability to distinguish FPs from ExPs is the limitation of dFLISA. For detailed characterization, analyses using orthogonal methods that can distinguish between FPs, EPs, PPs, and ExPs are desired.

We then calculated the LOQ, whose values for dFLISA were determined based on assay precision, accuracy, and background noise. It is remarkable that the LOQ values were close to those expected from the precision, accuracy, and the standard curve. Even the LOQ of dFLISA was only slightly higher than the LOQ of the ELISA. Nonetheless, it is sufficient to identify the capsid titers. Intermediate species cannot be quantified. It also indicates that further studies are needed to improve the sensitivity of the method for more accurate and precise detection and quantification of the analyte.

Linearity of AAV FP ratio in dFLISA analysis

dFLISA showed robust correlation and linearity in the FP ratio. The experimental ratio of full AAV particles was 0%–85.8%, with a precision of %CV 3.26% ± 25% (Figure 3), demonstrating good agreement with the expected FP ratio of 0%–90.1%. In addition, linearity experiments were performed using dFLISA over a range of ratios. Therefore, the reliable performance of this technique highlights its ability to discriminate between the different ratios of FPs. Furthermore, during the dFLISA demonstration, we improved the reliability and robustness of the method over multiple runs by introducing AAV vector samples at different concentrations and adjusting the FP ratio.

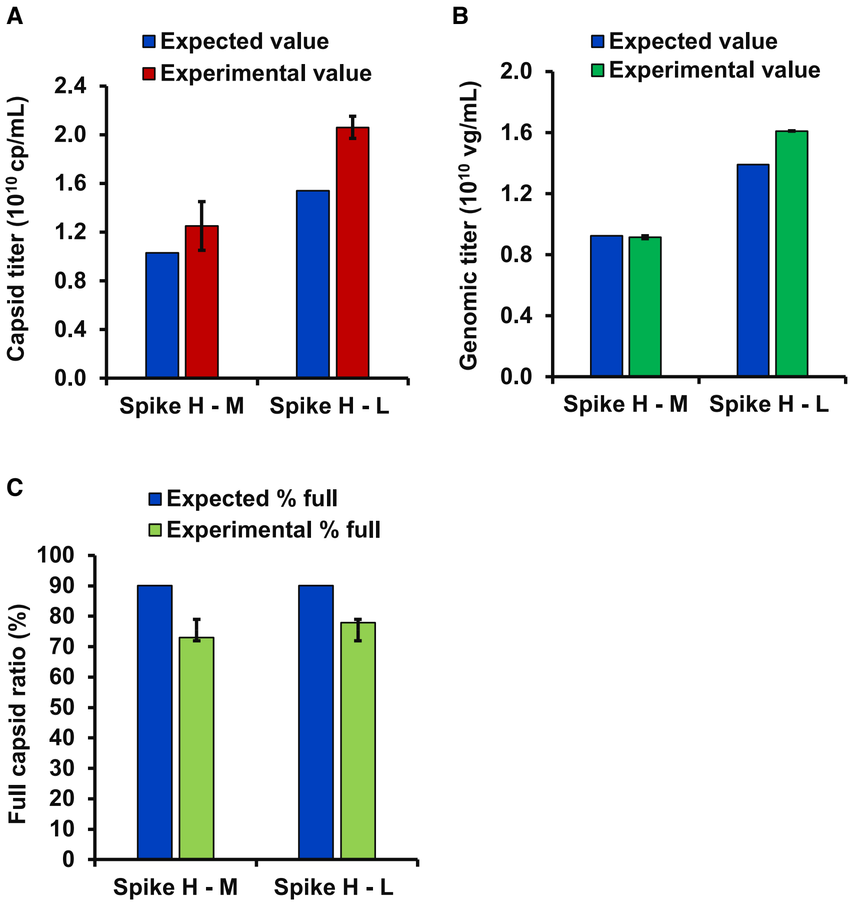

Figure 4. Applicability of dFLISA for analyzing crude samples

(A) Capsid titer quantification. (B) Genomic titer quantification. (C) Percentage of full capsids. The various dilution factors for spike recovery were assessed by comparing the experimental values, as determined by the dFLISA method, with the expected values obtained by BS-AUC analysis. The expected values derived from BS-AUC (blue) were compared with actual values for three parameters: capsid titer (red), genomic titer (green), and FP ratio (light green). Reported results represent the average measurements from duplicate wells. H, high concentration spike; M, middle concentration spike; L, low concentration spike. All data are presented as the mean and SD (n = 2).

This optimization not only minimized the duration of each assay, but also ensured a high level of consistency.

Comparison of dFLISA and orthogonal methods using mixed samples

The main approach for determining both capsid and genomic titers is to choose the most suitable analytical method for absolute quantification. BS-AUC is a standard technique used to analyze capsid content and distinguish between empty and full capsids as well as other AAV vector subspecies.[47,57] BS-AUC is based on the differential sedimentation velocities of AAV vector subpopulations under strong centrifugal force due to differences in size, density, weight, and shape. A combination of dPCR and ELISA is another standard method for the determination of the FP ratio. MP has recently gained popularity for AAV vector characterization because of its mass resolution, which allows operators to discriminate between empty and genome-filled capsids.[54,64,65]

In our study, we conducted a comparative analysis of the FP percentages in identical recombinant AAV samples via dFLISA, BS-AUC, MP, and dPCR/ELISA. The FP ratio determined by dFLISA was closer to the expected values than that determined by BS-AUC, MP, and dPCR/ELISA. The genomic titer determined by BS-AUC was lower than expected values (Figure S4), and the FP ratio determined by BS-AUC was lower than that of dFLISA (Figure 3). It should be noted that FP ratio was calculated by dividing the sum of FPs and ExPs by the sum of EPs, FPs, and ExPs and different from an FP ratio calculated only from EPs and FPs.[63,66]

Two peaks with mass corresponding to EPs and FPs were observed in MP analysis, and an ExP-related peak was not observed (Figure S5). A Gaussian distribution fit was applied to the two histogram peaks and FP ratio was calculated based on the peak area. The results of MP were consistent with that of dFLISA and both were as expected, with the exception of one sample containing 10% FPs. MP did not detect any EPs in the sample with 10% FPs, suggesting that dFLISA has a higher sensitivity than MP. The advantages and limitations of analytical methods used in this study are summarized in Table 1.

Previous studies have reported evidence showing that high-performance liquid chromatography (HPLC) is a rapid and convenient method for analyzing the empty and full capsid content of purified AAV samples.[67,68] The sensitivity of HPLC was sufficient to quantify the empty and full AAV vectors in samples with capsid concentrations as low as $\sim 5 \times 10^{10}$ cp/mL,[68] whereas dFLISA was able to quantify empty and full AAV vectors at concentrations as low as 0.60×10^{10} cp/mL (Table S5). Notably, the sensitivity of dFLISA was higher than that of HPLC, although HPLC is a promising method to determine FP ratio.

For capsid titer quantification, dFLISA demonstrated consistency with the ELISA (Table S7), but the LOQ of dFLISA (Table S5) was higher than that of traditional ELISA. This was probably due to differences in the detection method—the ELISA uses a horseradish-peroxidase-conjugated streptavidin enzyme[37,69] while dFLISA uses a second antibody conjugated to red fluorescence for capsid titer quantification. For

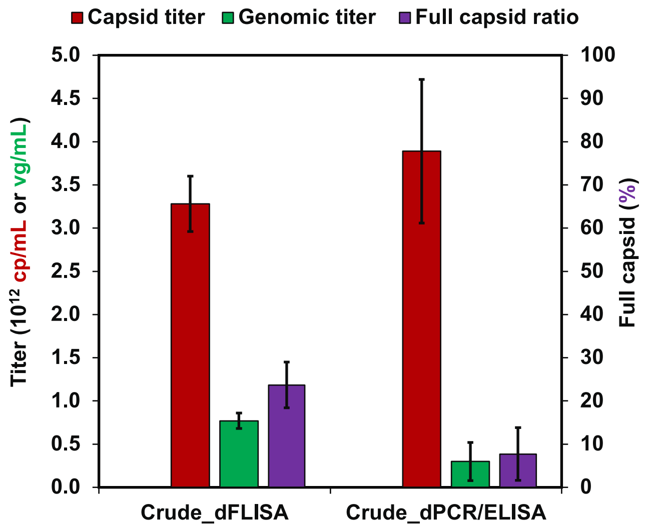

Figure 5. Determination of dFLISA's ability to quantify crude samples
AAV8 content in crude samples was analyzed by three methods: dFLISA (red, green, and purple), ELISA (red), and dPCR (green). dFLISA was used to quantify both capsid and genomic titers and the FP ratio of AAV8 content in crude samples. ELISA was used to quantify capsid AAV8 content and used the SD calculated from independent duplicate measurements (n = 2). The dPCR was used to determine the AAV8 content in crude samples and used the SD derived from independent triplicate measurements (n = 3). Therefore, dPCR/ELISA (purple) was used to compare FP ratios. All data presented in these experiments are the averages used to compare dFLISA and dPCR/ELISA methods' ability to quantify crude samples. The SD of each parameter was obtained from the triplicated experiments.

genomic titer measurement, dFLISA showed higher titers than dPCR for samples with 10.5%–90.1% FPs. This might be due to the variability of dPCR,[70,71] and, considering that the FP ratio determined by dFLISA was close to the expected values, genomic titer quantification by dFLISA should be more precise and accurate than dPCR. Another possible reason why the dPCR result was lower than the dFLISA result is that ExPs could contain a genome without inverted terminal repeat (ITR), and thus dPCR could not quantify ExPs because dPCR specifically quantifies a genome with ITR. The specificity of dFLISA was only for a genome, not a genome with ITR. The non-specificity is considered to be another limitation of dFLISA.

Comparing the methods from the perspective of the operator, it is important to note that BS-AUC and MP require specialized equipment and high capsid titers.[57] Conversely, the reliability of dFLISA as an alternative analytical method for the precise assessment of the FP ratio that uses the same standard has been demonstrated. Thus, it is a crucial test for quantifying FP, EP, and FP ratios. In FP ratio analysis by dPCR/ELISA, data from two independent analyses are required. Therefore, the combination method generally has inherently higher variability. Moreover, dPCR-based methods show higher variability than ELISA.[72]

The recovery efficiency of spiking levels was also assessed to see whether dFLISA can be used to analyze crude samples without purification. The recovery percentage was within ±25% of the expected values, which meets the criteria for acceptance. This suggests that the results of dFLISA are not affected by the matrix interferences contained in crude samples.[73,74] This highlights the suitability of the dFLISA method as a way to evaluate AAV samples that have not been purified, which offers a noteworthy advantage. Based on the results obtained, it is reasonable to conclude that the dFLISA method is well suited to the quantification of unpurified AAV vector samples. Therefore, dFLISA serves as a valuable and novel method that can be used to accurately quantify the titers of crude samples, making it uniquely capable of directly quantifying the capsid and genomic titer and FP ratio of crude samples.

While AUC requires the purification of crude samples prior to analysis, the capsid and genomic titers of untreated crude samples can be measured by dFLISA. The capsid titer determined by dFLISA was comparable with that determined by ELISA. However, the genomic titer results with dFLISA were higher than those from dPCR. The recovery rate in the spike-recovery experiment of dFLISA was high, whereas the spike-recovery result of dPCR was lower than expected values (Figure S6). In addition, previous studies demonstrated that dPCR can be affected by the interference of impurities.[70–72,75–80] This suggests that dFLISA results are relatively unaffected by matrix interference or impurities from the crude lysate, making it a reliable analytical technique for AAV vector particle analysis. The optimization of the dPCR method could provide better results, which is nevertheless beyond the scope of this study.

Considering the LOQ of dFLISA (1.61×10^{10} cp/mL, 1.70×10^{10} vg/mL) and the concentration of AAV at the end of upstream process (>10^{10} vg/mL), the sensitivity of dFLISA is high enough to analyze crude samples, although it would be difficult to analyze samples at the beginning of the upstream process.

The dPCR/ELISA combination approach is time-consuming and exhibits low accuracy, with reported coefficients of up to 36%.[74,81] In contrast, the entire dFLISA run was completed in less than 5 h. The simple data evaluation procedure contributes to the short duration, allowing for the analysis of more than 35 samples per day. Furthermore, the dFLISA allows for the straightforward quantification of purified AAV vectors as well as unpurified in-process samples. This is especially critical because there are no direct orthogonal methods available for quantifying crude samples without any purification.

Application of dFLISA analysis for diverse AAVs

We next aimed to expand the capabilities of dFLISA in this study. We thus demonstrated the suitability of the technique for quantifying the capsid and genomic titers for other AAV vector serotypes. The results show that dFLISA can be applied to other serotypes via a modification to the primary antibody.

To optimize our dFLISA, we used 85°C as the optimal temperature to disrupt the AAV capsid, and the appropriate temperature range for the disruption of AAV capsid particles AAV1 to AAV8 was between

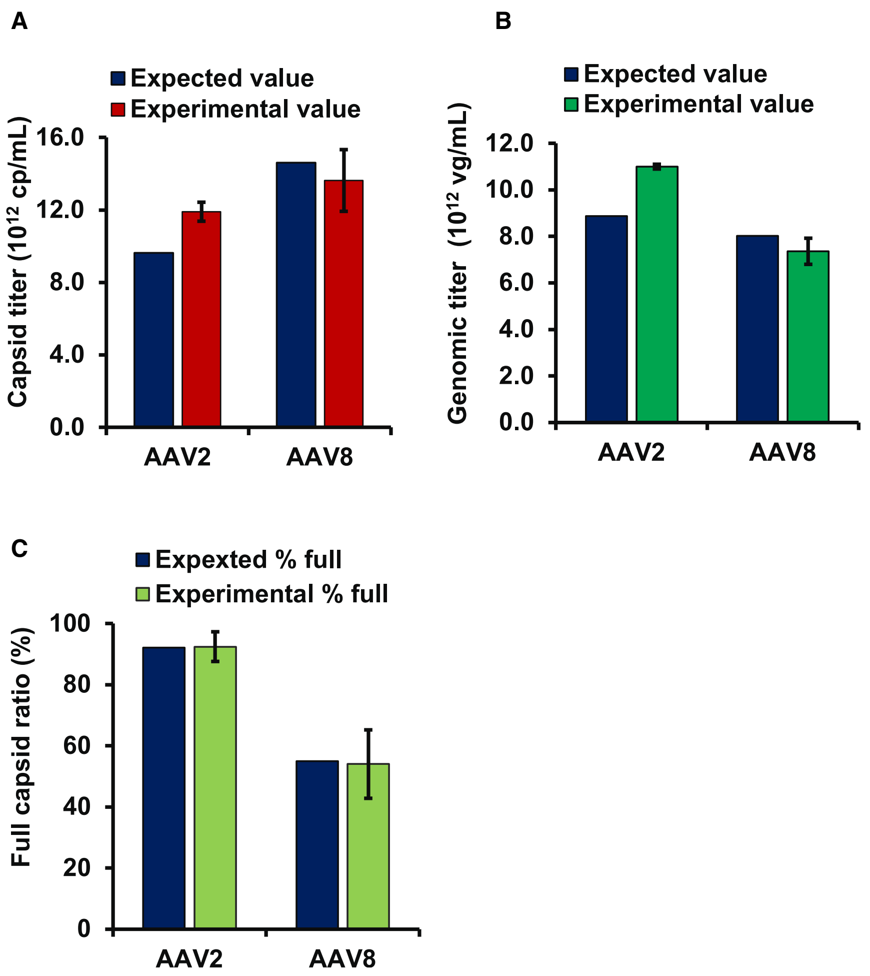

Figure 6. Application of dFLISA for quantification of different AAV serotypes

(A) Capsid titer quantification. (B) Quantification of genome titer. (C) Detection of FP ratio. dFLISA experiments were performed in duplicate for AAV2 and AAV8 vectors. Expected values derived from BS-AUC (dark navy blue) were compared with actual values for three parameters, specifically capsid titer (red), genomic titer (green), and FP ratio (light green). Results are the average of duplicated wells.

66.5°C and 89.5°C ± 0.5°C, with the exception of AAV5. As indicated in previous studies,[82] the specific temperature requirement for AAV5 disruption was 90°C ± 0.5°C. In addition, in our experiment, it was possible to use the AAV9 vector by simply modifying the ligand that coats the microtiter plate with the vector because the binding affinity of the VHH coating antibody is limited to the AAV9 vector.

The fluorescence intensity of the AAV vector varies with different genome lengths, and this factor is also relevant to dFLISA. All AAV vectors used in other experiments had genome lengths almost identical to that of the standard AAV vector. In addition, a comparison of fluorescence intensity between AAV vectors with different genome lengths validated the value and reliability of dFLISA as a method for evaluating AAV vector genomes. First, we used a mixture of ssDNA to evaluate the correlation between fluorescence intensity and DNA length. As shown in Figure 7, the relative fluorescence intensities were proportional to the genome lengths. Then, the differences in fluorescence intensities between two AAV vectors with different genome lengths (2,521 and 3,681 bases) were evaluated. According to the curve derived from the mixed DNA samples (Figure 7), the ratio of fluorescence intensity of the AAV with scDNA to that of the AAV with ssDNA was expected to be 1.40; however, the experimental value was 1.86 (Table S9). Therefore, although the fluorescence intensity of the ssDNA mixture was proportional to genome length, an interaction of DNA released from an AAV capsid with SYBR gold could be genome dependent probably due to the high temperature for capsid disruption and/or tertiary structure of DNA. If the length of an AAV genome is different from that of the standard AAV vector, we need to evaluate the difference in SYBR gold intensity between the sample and the standard prior to dFLISA analysis.

Conclusion

Our study introduces a novel analytical technique that allows for the accurate and precise measurement of the abundance of both full and empty AAV vector capsids, and the full/empty capsid ratio. The correlation between dFLISA and BS-AUC proved robust, indicating the reliability of the dFLISA results for both full and empty capsids. The dFLISA results also corresponded with those of other orthogonal techniques, including MP and a combination of dPCR and ELISA. Remarkably, dFLISA showed significant potential for evaluating the capsid and genome titers of unpurified samples and diverse AAV vector serotypes, offering versatility and eliminating the need for high levels of analytical expertise. Thus, the newly developed dFLISA presented in our paper has proven to be an invaluable method for the straightforward, accurate, and precise detection of AAV vector particles. Its potential utility offers significant opportunities for the advancement of AAV-based gene therapies.

MATERIALS AND METHODS

Materials

Recombinant AAVs

All rAAV vectors, including AAV8-Lot1, AAV8-Lot2, and AAV8-Lot3, were generated using triple-plasmid co-transfection. In brief, pAAV-Rep&Cap (Serotype 8), pAd helper, and transgene plasmids (CMV-EGFP or AAT-FIX) were co-transfected into suspended HEK293T or VPC 2.0 (Thermo Fisher Scientific, Waltham, MA) cells. The transfected cells were cultured, and the medium and

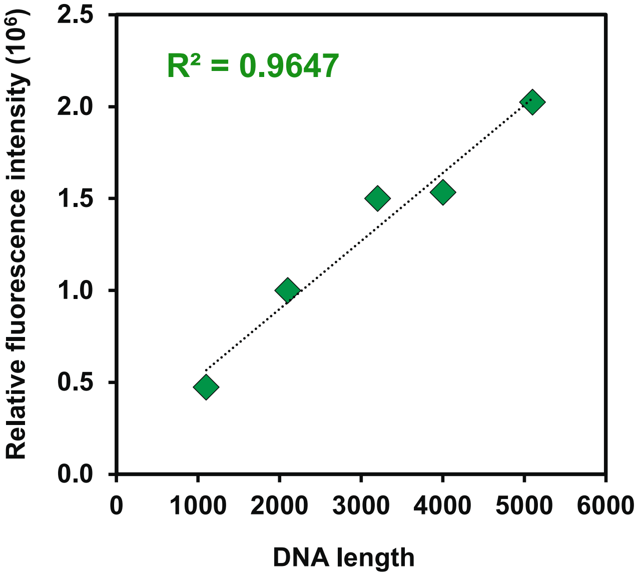

Figure 7. Comparison of fluorescence intensity of AAVs with different genome lengths

The fluorescence intensities of different ssDNA lengths derived from AGE divided by the MP percentage area derived from the contrast histogram were plotted against the length of the ssDNA ladder standard. Correlation between SYBR gold fluorescence intensity and lengths of ssDNA ladder fragments. AGE, agarose gel electrophoresis; MP, mass photometry; scDNA, self-complementary DNA; ssDNA, single-stranded DNA.

cell lysate were harvested (it was collected as a crude sample). Thereafter, the samples were purified via affinity chromatography using AAVX columns (Thermo Fisher Scientific). Bulk AAV samples were purified using affinity chromatographic purification followed by a CsCl ultracentrifugation (UC) or an anion exchange column to separate full and EPs. Purified samples (AAV8-Lot1 and AAV8-Lot2) were centrifuged at 25,000 rpm in an Optima XE-90 (Beckman Coulter, Brea, CA) using a Beckman SW41Ti rotor at 20°C for 42 h. For AAV8-Lot3, the purified sample was centrifuged at 34,000 rpm at 20°C for 72 h. The virus bands generated by UC were collected by using a piston fractionator (BioComp Instruments, Fredericton, Canada) equipped with a UV monitoring apparatus (Triax flow cell, BioComp Instruments). For the anion exchange chromatography, the samples were applied to a CIMmultus QA column (Sartorius, Göttingen, Germany) and eluted with a linear gradient of 0–250 mM NaCl in bis-tris-propane buffer (pH 9.0). Then the virus fractions were dialyzed in Slide-A-Lyzer 10K (Thermo Fisher Scientific). AAV samples were analyzed by BS-AUC prior to analysis (Figures S1–S3). Table S1 summarizes the information on the in-house AAV8 vectors used in this study. We also used other laboratory-grade AAV vectors, including AAV2-Lot1 to AAV2-Lot4 and AAV8-Lot5 to AAV8-Lot6 manufactured in HEK293T cells, which were procured from VectorBuilder (Chicago, IL). Table S2 summarizes the information on the commercial AAV2 vector and AAV8 vector used in the dFLISA experiments.

Reagents

CaptureSelect Biotin Anti-AAVX Conjugate (VHH) and BupH carbonate-bicarbonate buffer packs were purchased from Thermo Fisher Scientific. Lyophilized mouse anti-AAV2 monoclonal antibody (A20) and lyophilized mouse anti-AAV8 monoclonal antibody (ADK8) were purchased from Progen, (Heidelberg, Germany). Goat anti-mouse IgG H&L (Alexa Fluor 647) was purchased from Abcam (Cambridge, UK). SYBR Gold Nucleic Acid Gel staining solution was purchased from Invitrogen, Thermo Fisher Scientific (Eugene, OR). BSA and Tween 20 were purchased from Sigma Aldrich (St. Louis, MO). Sodium chloride (NaCl), purchased from BASF (Ludwigshafen, Germany), and PBS, purchased from Thermo Fisher Scientific, were used for buffer and solution preparation. Poloxamer-188 was kindly provided from BASF.

Methods

dFLISA

dFLISA was performed as shown in Figure 1. Black, 96-well, flat-bottomed MaxiSorp surface-treated immunoplates (Thermo Fisher Scientific) were used. These plates were coated with CaptureSelect Biotin Anti-AAVX Conjugate, a 14-kDa recombinant single-domain antibody fragment (VHH affinity ligand), at a concentration of 10 μg/mL (100-fold dilution) with BupH carbonate-bicarbonate buffer and the plates were incubated for 16 h at 4°C. The plates were washed three times with 0.05% Tween 20 in 1× PBS (pH 7.4). Prior to sample addition, addition of 200 μL of 1% BSA in 1× PBS was performed for blocking. An AAV vector sample in a formulation consisting of 1× PBS, 200 mM NaCl, and 0.001% (w/v) poloxamer-188 was used as sample. AAV8-Lot1 with a 2,521-bp genome was used as a standard. AAV8-Lot1 was diluted to a concentration of 2.38×10^{11} cp/mL and 2.18×10^{11} vg/mL in 0.05% Tween 20 in 1× PBS and then serially diluted at a 1:2 ratio to generate a calibration curve. Subsequently, 100 μL of sample and standard solutions were added to each well of the plate. To remove unbound components, the plate was subjected to a wash with 0.05% Tween 20 in 1× PBS. The lyophilized monoclonal anti-AAV8 antibody, ADK8, was reconstituted with 1 mL of Milli-Q water. ADK8 was diluted to a concentration of 1 μg/mL (50-fold dilution) with 1× PBS buffer at pH 7.4 containing 0.09% sodium azide and 0.5% BSA, followed by incubation at 37°C for 1 h, and three washes. The secondary antibody, goat anti-mouse IgG H&L (Alexa Fluor 647), was diluted to a concentration of 4 μg/mL (500-fold dilution) and added for labeling. The plate was then sealed with adhesive foil and incubated for 1 h at 37°C with shaking at 300 rpm. Following incubation, the plate was washed with wash buffer. Next, 100 μL of 1× PBS was added to each well, and plates were incubated at 85°C for 15 min. This process disrupted the viral capsid structure and released the ssDNA. Afterward, samples were allowed to cool at room temperature for 5 min. SYBR Gold Nucleic Acid Gel Stain solution was diluted 1,000-fold with 1× PBS. Subsequently, 10 μL of the diluted SYBR gold solution was added to individual wells, followed by a 5-min incubation at room temperature. Finally, we measured the intensity of the red fluorescence emanating from the proteins labeled with goat anti-mouse IgG H&L (Alexa Fluor 647) to quantify the capsid titers using an

Table 1. Most crucial performance criteria of analytical methods in this study

Methods	Target information	Purified sample	Turnaround (h)[a]	Sample volume (µL)	Sample concentration	Advantages	Limitations
BS-AUC	particle content and aggregate	yes	4–5	15–30	1×10^{12}–2×10^{13} cp/mL	capable of quantifying partially filled capsids and aggregates	requires specialized equipment purification is required prior to analysis of crude samples
dPCR	genomic titer	no	2–3	25	10^{7}–10^{10} vg/mL	specific and fast	low precision and accuracy
dFLISA	particle content	no	4.5–5	100	2×10^{10}–10^{11} vg/mL	high precision and accuracy purification is not necessary prior to analysis of crude samples	LOQ is slightly higher than the LOQ of ELISA; however, it is high enough to detect capsid titers PPs and ExPs cannot be distinguished from FPs
ELISA	capsid titer	no	4.5–5	100	10^{8}–10^{10} cp/mL	high specificity	low accuracy and precision due to two independent analyses
MP	particle content	yes	0.33–0.5	110	1×10^{11}–1×10^{12} vg/mL	capable of quantifying partially filled capsids, impurities, and aggregates low material requirements	requires specialized equipment purification is necessary prior to the analysis of crude samples

BS-AUC, band sedimentation analytical ultracentrifugation; dPCR, digital polymerase chain reaction; dFLISA, dual fluorescence-linked immunosorbent assay; ELISA, enzyme-linked immunosorbent assay; MP, mass photometry; ExP, extra filled particle; FP, full particle; LOQ, limit of quantification; PP, partial particle.
[a]Turnaround time includes sample preparation and data analysis.

excitation wavelength of 652 nm and an emission wavelength of 680 nm. In addition, we measured the intensity of the green fluorescence emanating from SYBR gold[56,83,84] to quantify the released genome using an excitation wavelength of 500 nm and an emission wavelength of 530 nm. A standard curve was generated using a four-parameter curve-fitting algorithm with the SpectraMax i3x microplate reader from Molecular Devices (San Jose, CA). Capsid and genomic titers of standards were calculated based on the results of BS-AUC. Because the concentrations of PPs were lower than LOQ of BS-AUC,[63] a sum of EPs, FPs, and ExPs was considered as capsid titer, and a sum of FPs and ExPs was considered as genomic titer. FP ratio was then calculated by dividing genomic titer by capsid titer. The amounts of cp/mL and vg/mL were then determined using the standard curve. Correction of SYBR gold intensity was not performed if the percent difference in genome length between the standard and the sample was within ±10%.

Quantification of capsid and genomic titers by dFLISA

Similarly, AAV8-Lot2 with a 2,712-base gene of interest was diluted 400-fold to a concentration of 1.54×10^{11} cp/mL and 1.39×10^{11} vg/mL in 0.05% Tween 20 in 1× PBS. Subsequently, serial dilutions at a 1:2 ratio were carried out to ensure precision, accuracy, and LOQ calculations. Measurements were conducted for three consecutive days (days 1–3) under identical operating conditions at short intervals using the same sample conditions and without any freeze-thaw cycles. The repeatability percentage was calculated by dividing the SD by the mean of three independent dFLISA results obtained by the same operator over 3 days (Equation 1):

$$\%CV = \frac{[\text{Mean of SD}]}{[\text{Mean of result}]} \times 100 \qquad \text{(Equation 1)}$$

Accuracy was calculated by finding the percentage difference between the value of capsid and genomic titers, as measured by dFLISA, and their expected values, as determined by BS-AUC.

Equation 2 was used with ±10% of expected values as the recovery percentage criterion for this calculation:

$$\%\text{ of accuracy} = \frac{[\text{Experimental value}]}{[\text{Expected value}]} \times 100 \qquad \text{(Equation 2)}$$

Determination of FP ratio by dFLISA

Linearity of FP ratio is critical in dFLISA to achieve optimal assay performance. We investigated precision, accuracy, linearity, and LOQ of FP ratio determined by dFLISA. The representative dFLISA procedure described above was used. Specifically, AAV8-Lot1 was diluted 60-fold to obtain a concentration of 2.38×10^{11} cp/mL and 2.18×10^{11} vg/mL with 0.05% Tween 20 in 1× PBS. Further serial dilutions were performed at a 1:2 ratio to construct a calibration curve. AAV8-Lot2 and AAV8-Lot3 were concentrated by ultrafiltration. We then mixed these concentrated samples at various ratios containing the following expected percentages of full capsids: 0%,

10.5%, 31.5%, 52.3%, 73.1%, and 90.1% of FPs. The mixed sample was then diluted 320-fold with 0.05% Tween 20 in 1× PBS. All samples were tested in duplicate. Each well of the plate was filled with 100 μL of the prepared sample solutions. The back-calculated concentrations of the calibration standards were maintained within ±25% of the value at the LOQ and within ±20% at all other levels.[73] The anchor calibrators (<LOQ) did not require acceptance criteria because they were beyond the quantifiable range of the curve.

Determination of full-to-empty ratio by BS-AUC

Experiments and analyses of BS-AUC were performed according to our previous study.[63] In brief, a buffer or AAV sample at a volume of 15 μL were loaded into a reference or sample reservoir well with a 12-mm band-forming centerpiece (Spin Analytical, South Berwick, ME) equipped with sapphire windows. A volume of 250 μL of PBS/D_2O containing 0.001% of poloxamer-188 was loaded into the reference or sample sector, respectively. Mixed samples (FPs in six prepared spike ratios, specifically, 90.1%, 73.1%, 52.1%, 31.5%, 10.5%, and 0% FPs) of AAV8 vectors were used. Data were collected at 20°C using Optima AUC (Beckman Coulter) at 20,000 rpm using a UV detection system, with the detection wavelength set at 280 nm. Data points were collected with a radial increment of 10 μm at an interval of 150 s

Sedimentation data were analyzed using the analytical zone centrifugation c(s) model of the program SEDFIT (version 16.2b),[85] in which parameters such as lamella width, frictional ratio, meniscus, time-invariant noise, and radial-invariant noise were adjusted and a regularization level of 0.68 was used. The s value range of 0–175 S was evaluated with a resolution of 350. The SEDNTERP program facilitated the calculation of buffer density and viscosity for the solvent loaded in the sectors.[86] The apparent sedimentation coefficient for FPs was converted to the sedimentation coefficient in water at 20°C ($s_{20,w}$). This conversion used the partial specific volume of the FPs, determined according to the procedure described in a previous study,[66] in conjunction with the buffer density and buffer viscosity. Subsequently, figures showing the c(s) distribution were generated using the program GUSSI (version 1.3.2).[87]

Particle concentrations were calculated by dividing the FP, EP, and ExP peak areas by respective molar extinction coefficient at the detection wavelength. The FP ratio was calculated by dividing the sum of FPs and ExPs by the sum of FPs, EPs, and ExPs. The mean s_{20w}, FP ratio, and SD of each parameter were calculated based on the results obtained from the triplicate experiments.

Determination of capsid titers of AAV8 vector by ELISA

An AAV8 titration kit (Progen) was used to determine capsid titers. The assay was performed according to the manufacturer's instructions. A series of 2-fold dilutions of the kit's standard viruses were made to generate a capsid standard curve ranging from 7.97 × 10^6 to 5.01 × 10^8 cp/mL. Mixed samples (full capsids in six prepared spike ratios, specifically, 90.1%, 73.1%, 52.3%, 31.5%, 10.5%, and 0% FPs) of AAV8 vector were diluted with 0.05% Tween 20 in 1× PBS. All measurements, including unknown samples and blanks, were performed in duplicate at three different dilutions. The mean value was used to calculate AAV8 titers. A prepared 100 μL sample was added to a microwell plate and incubated for 1 h at 37°C. The microwell plate was then washed three times with wash buffer. The biotinylated anti-AAV8 antibody (ADK8) was then added to the microwell plate, and the plate was incubated for 1 h at 37°C. The washing step was repeated. Streptavidin-horse radish peroxidase conjugate was added and incubated for 1 h at 37°C, followed by the washing and the addition of ready-to-use tetramethylbenzidine solution to the wells, which were then incubated for 15 min at room temperature. The color reaction was stopped by adding ready-to-use sulfuric acid solution. Absorbance was then measured photometrically at 450 nm with a SpectraMax 3× microplate reader. The readings of each sample were then averaged to determine the final titers using a four-parameter logistic (4PL) curve-fitting model. The 4PL standard curve was generated in Microsoft Excel by plotting the subtracted optical density measurements of the serially diluted kit controls against the corresponding AAV vector concentrations.

Determination of genomic titers of mixed samples of AAV8 vector using dPCR

AAV vector samples with various FP ratio (full capsids in six prepared ratios, specifically, 90.1%, 73.1%, 52.3%, 31.5%, 10.5%, and 0% FPs) were prepared as described above and then treated with DNase I (Takara, Japan). The samples were then incubated at 37°C for 30 min to digest any unpackaged DNA. Subsequently, a solution of 0.25 mM ethylenediaminetetraacetic acid (EDTA) (Nippon Gene, Japan) was added. The mixture was incubated at room temperature for 5 min. Afterward, the mixture was heated to 95°C for 15 min to inactivate the DNase I enzyme and denature the viral capsid. Dilution buffer was prepared by adding poloxamer-188 to Tris-EDTA buffer to achieve a final concentration of 0.001%. This dilution buffer was used to dilute the test samples to the appropriate range for analysis. Each dPCR reaction was set up to a final volume of 10 μL consisting of 1 μL of the prepared sample solution, 2 μL of 5× dPCR QuantStudio Absolute Q Master Mix (Thermo Fisher Scientific), 1.8 μL ITR primers (forward and reverse), and 0.25 μL ITP probe mix (purchased from Hokkaido System Science); 9 μL of dPCR reaction mix was added to each well of a QuantStudio Absolute Q MAP16 Plate Kit (Thermo Fisher Scientific). Afterward, 15 μL of QuantStudio Absolute Q Isolation Buffer (Thermo Fisher Scientific) was carefully added to each well on top of the reaction mix. The wells were sealed with QuantStudio Absolute Q Strip Caps (Thermo Fisher Scientific) and centrifuged at 1,200 rpm for 1 min on a swing-out rotor. The assay was performed on a QuantStudio Absolute Q Digital PCR System (Thermo Fisher Scientific). Thermal cycling was performed as follows: (1) preheat at 96°C for 10 min, then (2) 40 cycles consisting of denaturation at 94°C for 5 s followed by annealing/extension at 54°C for 30 s. Data and global threshold were analyzed using QuantStudio Absolute Q digital PCR software (Thermo Fisher Scientific). Sample dilutions were used to calculate AAV genomic titers.

Determination of FP ratios of AAV8 vector by MP

MP measurements were conducted using TwoMP (Refeyn, Oxford, UK). For each experiment, mixed samples (full capsids at six prepared sample ratios, specifically, 90.1%, 73.1%, 52.3%, 31.5%, 10.5%, and 0% FPs) of AAV8 vector were pre-diluted in PBS (Gibco). Precision cover glasses (ThorLabs, Tokyo, Japan) were meticulously cleaned by serial rinsing with Milli-Q water and ethanol. To create the measurement chambers, we attached a pre-cut 2 × 3 well CultureWell silicone seal (3 mm diameter × 1 mm depth, Grace Bio-Labs, Bend, OR) to the clean coverslips. The coverslips were then transferred to the MP instrument, and 18 μL of PBS buffer was added to each well. After focusing, 2 μL of each AAV vector solution was added and mixed into the wells to achieve a total filling volume of 20 μL. Each measurement was recorded for 60 s, and each sample was analyzed at least three times ($n \geq 3$). Data analysis was performed using DiscoverMP version 2.5.1 (Refeyn). A Gaussian distribution fit was applied to the histogram peaks. From these Gaussian fits, we extracted the percentage of filled and empty AAV capsids.

Quantification of crude sample by dFLISA and other methods

A representative method for dFLISA is described above. In these experiments, AAV8-Lot1 was used as standard as described above. For the spike sample, AAV8-Lot2 was concentrated by ultrafiltration to reach a final concentration of 6.16×10^{13} cp/mL and 5.55×10^{13} vg/mL. It was then diluted to three different spiking levels: high (spike H) at 1.23×10^{11} cp/mL and 1.11×10^{11} vg/mL, medium (spike M) at 0.82×10^{11} cp/mL and 0.74×10^{10} vg/mL, and low (spike L) at 0.61×10^{11} cp/mL and 0.55×10^{11} vg/mL, all in 0.05% Tween 20 in 1× PBS. A crude lysate sample of unknown concentration was mixed with each spike sample solution at a 1:1 volume ratio and analyzed in duplicate. The following equations were used to calculate the expected values for mixed spike samples:

$$\text{Mixed spike(H–M)} = \frac{1}{2}(\text{crude} + \text{spike H}) - \frac{1}{2}(\text{crude} + \text{spike M}) \quad \text{(Equation 3)}$$

$$\text{Mixed spike (H–M)} = \frac{1}{2}(\text{crude} + \text{spike H}) - \frac{1}{2}(\text{crude} + \text{spike L}) \quad \text{(Equation 4)}$$

The prepared spike sample solutions were then added to each well of the plate. The target recovery percentage (%) was within ±25%.

Quantification of capsid and genomic titers of crude samples

For this experiment, the representative dFLISA method described above was used. An AAV8 crude lysate sample of an unknown concentration was analyzed by dFLISA, as detailed in the preceding section. The analysis was performed in triplicate. The capsid and genomic titers obtained by dFLISA were compared with ELISA and dPCR results by independent samples t test.

Application of dFLISA for quantifying different AAV serotypes

The representative dFLISA method described above was used for this experiment. AAV2-Lot3, which contained linear ssDNA, was diluted 100-fold to reach concentrations of 1.77×10^{11} cp/mL and 1.65×10^{11} vg/mL with 0.05% Tween 20 in 1× PBS, then serially diluted 1:2 to generate a calibration curve. AAV2-Lot4 was also diluted 100-fold, resulting in final concentrations of 9.63×10^{10} cp/mL and 8.88×10^{10} vg/mL, with 0.05% Tween 20 in 1× PBS. The AAV8-Lot5 sample containing linear ssDNA was diluted 50-fold to reach concentrations of 2.06×10^{11} cp/mL and 1.59×10^{11} vg/mL with 0.05% Tween 20 in 1× PBS, and then serially diluted 1:2 to generate a calibration curve. AAV8-Lot6 was diluted 50-fold to achieve final concentrations of 2.92×10^{11} cp/mL and 1.60×10^{11} vg/mL with 0.05% Tween 20 in 1× PBS. Test samples prepared without any freeze-thaw cycles.

Fluorescence intensity with different genome lengths

A ssDNA 7K ladder (PerkinElmer, Waltham, MA) containing ssDNA fragments ranging from 1,100 bases to 5,100 bases was used. First, 2 μL of EzApplyDNA (6× loading buffer) was applied to Parafilm for each sample. Next, 10 μL of sample was added and thoroughly mixed by pipetting, and 10 μL of the mixture was loaded onto a 1% agarose gel (Funakoshi, Japan). Electrophoresis was performed at 70 V for 45 min, after which the gel was stained according to the manufacturer's instructions. SYBR Gold Nucleic Acid Gel staining solution (Invitrogen, Thermo Fisher Scientific, Eugene, OR) was used for gel staining. Quantitative analysis of brightness density within the stained gel was performed using an iBright 1500 instrument (Thermo Fisher Scientific) in conjunction with iBright Analysis version 4.0 software (Thermo Fisher Scientific). Image brightness adjustments were made prior to analysis.

Subsequently, the ssDNA ladder was analyzed by MP to determine the relative concentration of each ssDNA in the ladder. Before conducting MP measurements, ssDNA ladder solutions were diluted in buffer consisting of 5 mM Tris and 10 mM $MgCl_2$ (pH 8). Each measurement was recorded for 60 s, and every sample was examined a minimum of three times ($n \geq 3$). Data analysis was conducted using DiscoverMP and an in-house Python program. Histogram peaks were fitted with Gaussian distributions to extract the percentage of ssDNA ratio.

Then, the fluorescence intensities obtained by agarose gel electrophoresis with five different ssDNA ladder strands were divided by MP area (%) to obtain relative fluorescence intensity per molecule. The relative fluorescence intensity was plotted against the ssDNA ladder standard length. The expected fluorescence intensity ratio of the AAV vectors with both scDNA (3,681 bp) and ssDNA (2,521 bp) was also estimated from the curve.

The fluorescence intensity of AAV-containing scDNA was examined using dFLISA and compared with that of AAV-containing ssDNA. AAV2-Lot1-containing ssDNA was diluted 100-fold to concentrations of 3.09×10^{10} cp/mL and 2.82×10^{10} vg/mL in 0.05% Tween 20 in 1× PBS, and then serially diluted at a 1:2 ratio to generate a calibration curve. Similarly, AAV2-Lot2-containing scDNA was diluted 50-fold to concentrations of 7.47×10^{10} cp/mL and

6.66×10^{10} vg/mL in 0.05% Tween 20 in 1× PBS and then serially diluted at a 1:2 ratio to generate a calibration curve. The dFLISA analysis method as described earlier in the methods was used. The ratio of fluorescence intensities of ssDNA AAV8 and scDNA AAV2 were calculated.

DATA AND CODE AVAILABILITY

Data will be made available on request.

SUPPLEMENTAL INFORMATION

Supplemental information can be found online at https://doi.org/10.1016/j.omtm.2024.101291.

ACKNOWLEDGMENTS

This research was supported by grant-in-aid from “Research and development of core technologies for gene and cell therapy” supported by Japan Agency for Medical Research and Development (AMED) (grant no. JP20ae0201002), Japan and the Ministry of Education, Culture, Sports, Science and Technology, Japan. We thank Suzanne Leech, PhD, from Edanz (https://jp.edanz.com/ac) for editing a draft of this manuscript.

AUTHOR CONTRIBUTIONS

Conceptualization, S.S., T.T., and S.U.; investigation, S.S., M.T., M.S., T.H., T.M., and M.F.; data curation, S.S. and M.T.; resources, K.H., T.O., M.F., T.M., and Y.T.; writing – original draft, S.S., T.T., and S.U.; writing – review & editing, T.T. and S.U.

DECLARATION OF INTERESTS

T.M. and M.F. are employees of U-medico Inc. S.U. is an employee and shareholder of U-medico Inc., and is a member of the scientific advisory board of Coriolis Pharma. S.S., M.T., T.T., and S.U. are applicants for a patent related to this work.

DECLARATION OF GENERATIVE AI AND AI-ASSISTED TECHNOLOGIES IN THE WRITING PROCESS

During the preparation of this work the authors used DeepL Translate to improve readability and language. After using the tools, the authors reviewed and edited the content as needed and take full responsibility for the content of the publication.

REFERENCES

1. Cole, L., Fernandes, D., Hussain, M.T., Kaszuba, M., Stenson, J., and Markova, N. (2021). Characterization of recombinant adeno-associated viruses (rAAVs) for gene therapy using orthogonal techniques. Pharmaceutics *13*, 586. https://doi.org/10.3390/pharmaceutics13040586.
2. Huttner, N.A., Girod, A., Perabo, L., Edbauer, D., Kleinschmidt, J.A., Büning, H., and Hallek, M. (2003). Genetic modifications of the adeno-associated virus type 2 capsid reduce the affinity and the neutralizing effects of human serum antibodies. Gene Ther. *10*, 2139–2147. https://doi.org/10.1038/sj.gt.3302123.
3. Sommer, J.M., Smith, P.H., Parthasarathy, S., Isaacs, J., Vijay, S., Kieran, J., Powell, S.K., McClelland, A., and Wright, J.F. (2003). Quantification of adeno-associated virus particles and empty capsids by optical density measurement. Mol. Ther. *7*, 122–128. https://doi.org/10.1016/S1525-0016(02)00019-9.
4. Yin, V., Devine, P.W.A., Saunders, J.C., Hines, A., Shepherd, S., Dembek, M., Dobson, C.L., Snijder, J., Bond, N.J., and Heck, A.J.R. (2022). Spectral Interferences Impede the High-Resolution Mass Analysis of Recombinant Adeno-Associated Viruses. Preprint at bioRxiv *7*, 122. https://doi.org/10.1101/2022.08.27.505551.
5. Shirley, J.L., de Jong, Y.P., Terhorst, C., and Herzog, R.W. (2020). Immune Responses to Viral Gene Therapy Vectors. Mol. Ther. *28*, 709–722. https://doi.org/10.1016/j.ymthe.2020.01.001.
6. Nyberg, W.A., Ark, J., To, A., Clouden, S., Reeder, G., Muldoon, J.J., Chung, J.Y., Xie, W.H., Allain, V., Steinhart, Z., et al. (2023). An evolved AAV variant enables efficient genetic engineering of murine T cells. Cell *186*, 446–460.e19. https://doi.org/10.1016/j.cell.2022.12.022.
7. Wobus, C.E., Hügle-Dörr, B., Girod, A., Petersen, G., Hallek, M., and Kleinschmidt, J.A. (2000). Monoclonal Antibodies against the Adeno-Associated Virus Type 2 (AAV-2) Capsid: Epitope Mapping and Identification of Capsid Domains Involved in AAV-2–Cell Interaction and Neutralization of AAV-2 Infection. J. Virol. *74*, 9281–9293. https://doi.org/10.1128/jvi.74.19.9281-9293.2000.
8. Ylä-Herttuala, S. (2012). Endgame: Glybera finally recommended for approval as the first gene therapy drug in the European union. Mol. Ther. *20*, 1831–1832. https://doi.org/10.1038/mt.2012.194.
9. Escandell, J.M., Pais, D.A., Carvalho, S.B., Vincent, K., Gomes-Alves, P., and Alves, P.M. (2022). Leveraging rAAV bioprocess understanding and next generation bioanalytics development. Curr. Opin. Biotechnol. *74*, 271–277. https://doi.org/10.1016/j.copbio.2021.12.009.
10. Pei, X., Shao, W., Xing, A., Askew, C., Chen, X., Cui, C., Abajas, Y.L., Gerber, D.A., Merricks, E.P., Nichols, T.C., et al. (2020). Development of AAV Variants with Human Hepatocyte Tropism and Neutralizing Antibody Escape Capacity. Mol. Ther. Methods Clin. Dev. *18*, 259–268. https://doi.org/10.1016/j.omtm.2020.06.003.
11. Barnes, L.F., Draper, B.E., Kurian, J., Chen, Y.T., Shapkina, T., Powers, T.W., and Jarrold, M.F. (2023). Analysis of AAV-Extracted DNA by Charge Detection Mass Spectrometry Reveals Genome Truncations. Anal. Chem. *95*, 4310–4316. https://doi.org/10.1021/acs.analchem.2c04234.
12. Bee, J.S., O'Berry, K., Zhang, Y.Z., Phillippi, M.K., Kaushal, A., DePaz, R.A., and Marshall, T. (2021). Quantitation of Trace Levels of DNA Released from Disrupted Adeno-Associated Virus Gene Therapy Vectors. J. Pharmaceut. Sci. *110*, 3183–3187. https://doi.org/10.1016/j.xphs.2021.06.010.
13. Ciatto, C., Yarawsky, A.E., Figuero, N., Burgner, J.W., Slade, P.G., and Paul, L.N. 2000 Quantitation of AAV Ratios in a Dual-Vector System Using SV-AUC.
14. Halder, S., Van Vliet, K., Smith, J.K., Duong, T.T.P., McKenna, R., Wilson, J.M., and Agbandje-McKenna, M. (2015). Structure of neurotropic adeno-associated virus AAVrh.8. J. Struct. Biol. *192*, 21–36. https://doi.org/10.1016/j.jsb.2015.08.017.
15. Deverman, B.E., Ravina, B.M., Bankiewicz, K.S., Paul, S.M., and Sah, D.W.Y. (2018). Gene therapy for neurological disorders: Progress and prospects. Nat. Rev. Drug Discov. *17*, 641–659. https://doi.org/10.1038/nrd.2018.110.
16. Xu, G., Zhang, R., Li, H., Yin, K., Ma, X., and Lou, Z. (2022). Structural basis for the neurotropic AAV9 and the engineered AAVPHP.eB recognition with cellular receptors. Mol. Ther. Methods Clin. Dev. *26*, 52–60. https://doi.org/10.1016/j.omtm.2022.05.009.
17. West, A.C., Federspiel, J.D., Rogers, K., and Khatri, A. (2023). Complement Activation by AAV-Neutralizing Antibody Complexes. Hum. Gene Ther. 1–29. https://doi.org/10.1089/hum.2023.018.
18. Barnes, C., Scheideler, O., and Schaffer, D. (2019). Engineering the AAV capsid to evade immune responses. Curr. Opin. Biotechnol. *60*, 99–103. https://doi.org/10.1016/j.copbio.2019.01.002.
19. Hiemenz, C., Pacios-Michelena, A., Helbig, C., Vezočnik, V., Strebl, M., Nikels, F., Hawe, A., Garidel, P., and Menzen, T. (2023). Characterization of Virus Particles and Submicron-Sized Particulate Impurities in Recombinant Adeno-Associated Virus Drug Product. J. Pharmaceut. Sci. *112*, 2190–2202. https://doi.org/10.1016/j.xphs.2023.05.009.
20. Grande, A.E., Li, X., Miller, L.M., Zhang, J., Draper, B.E., Herzog, R.W., Xiao, W., and Jarrold, M.F. (2023). Antibody Binding to Recombinant Adeno Associated Virus Monitored by Charge Detection Mass Spectrometry. Anal. Chem. *95*, 10864–10868. https://doi.org/10.1021/acs.analchem.3c02371.

21. Ebberink, E.H.T.M., Ruisinger, A., Nuebel, M., Thomann, M., and Heck, A.J.R. (2022). Assessing production variability in empty and filled adeno-associated viruses by single molecule mass analyses. Mol. Ther. Methods Clin. Dev. *27*, 491–501. https://doi.org/10.1016/j.omtm.2022.11.003.

22. Yarawsky, A.E., Zai-Rose, V., Cunningham, H.M., Burgner, J.W., DeLion, M.T., and Paul, L.N. (2023). AAV analysis by sedimentation velocity analytical ultracentrifugation: beyond empty and full capsids. Eur. Biophys. J. *52*, 353–366. https://doi.org/10.1007/s00249-023-01646-z.

23. Stagg, S.M., Yoshioka, C., Davulcu, O., and Chapman, M.S. (2022). Cryo-electron Microscopy of Adeno-associated Virus. Chem. Rev. *122*, 14018–14054. https://doi.org/10.1021/acs.chemrev.1c00936.

24. Sternisha, S.M., Wilson, A.D., Bouda, E., Bhattacharya, A., and VerHeul, R. (2023). Optimizing high-throughput viral vector characterization with density gradient equilibrium analytical ultracentrifugation. Eur. Biophys. J. *52*, 387–392. https://doi.org/10.1007/s00249-023-01654-z.

25. Hajba, L., and Guttman, A. (2020). Recent Advances in the Analysis Full/Empty Capsid Ratio and Genome Integrity of Adeno-associated Virus (AAV) Gene Delivery Vectors. Curr. Mol. Med. *20*, 806–813. https://doi.org/10.2174/1566524020999200730181042.

26. Selvaraj, N., Wang, C.K., Bowser, B., Broadt, T., Shaban, S., Burns, J., Saptharishi, N., Pechan, P., Golebiowski, D., Alimardanov, A., et al. (2021). Detailed Protocol for the Novel and Scalable Viral Vector Upstream Process for AAV Gene Therapy Manufacturing. Hum. Gene Ther. *32*, 850–861. https://doi.org/10.1089/hum.2020.054.

27. Richter, K., Wurm, C., Strasser, K., Bauer, J., Bakou, M., VerHeul, R., Sternisha, S., Hawe, A., Salomon, M., Menzen, T., and Bhattacharya, A. (2023). Purity and DNA content of AAV capsids assessed by analytical ultracentrifugation and orthogonal biophysical techniques. Eur. J. Pharm. Biopharm. *189*, 68–83. https://doi.org/10.1016/j.ejpb.2023.05.011.

28. Werle, A.K., Powers, T.W., Zobel, J.F., Wappelhorst, C.N., Jarrold, M.F., Lyktey, N.A., Sloan, C.D.K., Wolf, A.J., Adams-Hall, S., Baldus, P., and Runnels, H.A. (2021). Comparison of analytical techniques to quantitate the capsid content of adeno-associated viral vectors. Mol. Ther. Methods Clin. Dev. *23*, 254–262. https://doi.org/10.1016/j.omtm.2021.08.009.

29. Wörner, T.P., Snijder, J., Friese, O., Powers, T., and Heck, A.J.R. (2022). Assessment of genome packaging in AAVs using Orbitrap-based charge-detection mass spectrometry. Mol. Ther. Methods Clin. Dev. *24*, 40–47. https://doi.org/10.1016/j.omtm.2021.11.013.

30. Li, T., Gao, T., Chen, H., Demianova, Z., Wang, F., Luo, J., Yowanto, H., and Mollah, S. (2020). Determination of Full, Partial and Empty Capsid Ratios for Adeno-Associated Virus (AAV) Analysis. SCIEX Application Note, p1–p4.

31. Werling, N.J., Satkunanathan, S., Thorpe, R., and Zhao, Y. (2015). Systematic Comparison and Validation of Quantitative Real-Time PCR Methods for the Quantitation of Adeno-Associated Viral Products. Hum. Gene Ther. Methods *26*, 82–92. https://doi.org/10.1089/hgtb.2015.013.

32. Zölls, S., Tantipolphan, R., Wiggenhorn, M., Winter, G., Jiskoot, W., Friess, W., and Hawe, A. (2012). Particles in Therapeutic Protein Formulations, Part 1: Overview of Analytical Methods (John Wiley and Sons Inc), pp. 914–935. https://doi.org/10.1002/jps.23001.

33. Roesch, A., Zölls, S., Stadler, D., Helbig, C., Wuchner, K., Kersten, G., Hawe, A., Jiskoot, W., and Menzen, T. (2022). Particles in Biopharmaceutical Formulations, Part 2: An Update on Analytical Techniques and Applications for Therapeutic Proteins. J. Pharm. Sci. *111*, 933–950. https://doi.org/10.1016/j.xphs.2021.12.011.

34. Wu, D., and Piszczek, G. (2021). Standard protocol for mass photometry experiments. Eur. Biophys. J. *50*, 403–409. https://doi.org/10.1007/s00249-021-01513-9.

35. Lai, S.H., Tamara, S., and Heck, A.J.R. (2021). Single-particle mass analysis of intact ribosomes by mass photometry and Orbitrap-based charge detection mass spectrometry. iScience *24*, 103211. https://doi.org/10.1016/j.isci.2021.103211.

36. Grimm, D., Kern, A., Pawlita, M., Ferrari, F.K., Samulski, R.J., and Kleinschmidt, J.A. (1999). Titration of AAV-2 Particles via a Novel Capsid ELISA: Packaging of Genomes Can Limit Production of Recombinant AAV-2. Gene Ther. *6*, 1322–1330.

37. Gardner, M.R., Mendes, D.E., Muniz, C.P., Martinez-Navio, J.M., Fuchs, S.P., Gao, G., and Desrosiers, R.C. (2022). High concordance of ELISA and neutralization assays allows for the detection of antibodies to individual AAV serotypes. Mol. Ther. Methods Clin. Dev. *24*, 199–206. https://doi.org/10.1016/j.omtm.2022.01.003.

38. Klumpp-Thomas, C., Kalish, H., Drew, M., Hunsberger, S., Snead, K., Fay, M.P., Mehalko, J., Shunmugavel, A., Wall, V., Frank, P., et al. (2021). Standardization of ELISA protocols for serosurveys of the SARS-CoV-2 pandemic using clinical and at-home blood sampling. Nat. Commun. *12*, 113. https://doi.org/10.1038/s41467-020-20383-x.

39. Sanmiguel, J., Gao, G., and Vandenberghe, L.H. (2019). Quantitative and digital droplet-based AAV genome titration. In Methods in Molecular Biology (Humana Press Inc), pp. 51–83. https://doi.org/10.1007/978-1-4939-9139-6_4.

40. François, A., Bouzelha, M., Lecomte, E., Broucque, F., Penaud-Budloo, M., Adjali, O., Moullier, P., Blouin, V., and Ayuso, E. (2018). Accurate Titration of Infectious AAV Particles Requires Measurement of Biologically Active Vector Genomes and Suitable Controls. Mol. Ther. Methods Clin. Dev. *10*, 223–236. https://doi.org/10.1016/j.omtm.2018.07.004.

41. Veldwijk, M.R., Topaly, J., Laufs, S., Hengge, U.R., Wenz, F., Zeller, W.J., and Fruehauf, S. (2002). Development and optimization of a real-time quantitative PCR-based method for the titration of AAV-2 vector stocks. Mol. Ther. *6*, 272–278. https://doi.org/10.1006/mthe.2002.0659.

42. SAITO, S., KONDO, A., and UCHIDA, K. (2023). Investigating critical thermal parameters for pre-analytical preparation of adeno-associated virus vector genome titration by droplet digital polymerase chain reaction. Transl. Regul. Sci. *5*, 28–35. https://doi.org/10.33611/trs.2023-001.

43. Blay, E., Hardyman, E., and Morovic, W. (2023). PCR-based analytics of gene therapies using adeno-associated virus vectors: Considerations for cGMP method development. Mol. Ther. Methods. Clin. Dev. *31*, 101132. https://doi.org/10.1016/j.omtm.2023.101132.

44. Meierrieks, F., Kour, A., Pätz, M., Pflanz, K., Wolff, M.W., and Pickl, A. (2023). Unveiling the secrets of adeno-associated virus: novel high-throughput approaches for the quantification of multiple serotypes. Mol. Ther. Methods Clin. Dev. *31*, 101118. https://doi.org/10.1016/j.omtm.2023.101118.

45. He, X.Z., Powers, T.W., Huang, S., Liu, Z., Shi, H., Orlet, J.D., Mo, J.J., Srinivasan, S., Jacobs, S., Zhang, K., et al. (2023). Development of an icIEF assay for monitoring AAV capsid proteins and application to gene therapy products. Mol. Ther. Methods Clin. Dev. *29*, 133–144. https://doi.org/10.1016/j.omtm.2023.03.002.

46. Pañ Eda, A., Vanrell, L., Mauleon, I., Crettaz, J.S., Berraondo, P., Timmermans, E.J., Beattie, S.G., Twisk, J., Van Deventer, S., Prieto, J., et al. (2009). Effect of Adeno-Associated Virus Serotype and Genomic Structure on Liver Transduction and Biodistribution in Mice of Both Genders. Hum. Gene Ther. *20*, 908–917.

47. Hayes, D.B., and Dobnik, D. (2022). Commentary: Multiplex dPCR and SV-AUC Are Promising Assays to Robustly Monitor the Critical Quality Attribute of AAV Drug Product Integrity. J. Pharm. Sci. *111*, 2143–2148. https://doi.org/10.1016/j.xphs.2022.04.010.

48. Fu, Q., Lee, Y.S., Green, E.A., Wang, Y., Park, S.Y., Polanco, A., Lee, K.H., Betenbaugh, M., McNally, D., and Yoon, S. (2023). Design space determination to optimize DNA complexation and full capsid formation in transient rAAV manufacturing. Biotechnol. Bioeng. *120*, 3148–3162. https://doi.org/10.1002/bit.28508.

49. Xiang, Y.S., and Hao, G.G. (2023). Biophysical Characterization of Adeno-Associated Virus Capsid through the Viral Transduction Life Cycle. J. Genet. Eng. Biotechnol. *21*, 62. https://doi.org/10.1186/s43141-023-00518-5.

50. Aebischer, M.K., Bouvarel, T., Barrozo, E., Kochardt, D., Elger, C., Haindl, M., Ruppert, R., Guillarme, D., and D'Atri, V. (2023). Boosting the Separation of Adeno-Associated Virus Capsid Proteins by Liquid Chromatography and Capillary Electrophoresis Approaches. Int. J. Mol. Sci. *24*, 8503. https://doi.org/10.3390/ijms24108503.

51. Overturf, K. (2009). Quantitative PCR. In Molecular Research in Aquaculture (Wiley-Blackwell), pp. 39–61. https://doi.org/10.1002/9780813807379.ch3.

52. Maestro, S., Weber, N.D., Zabaleta, N., Aldabe, R., and Gonzalez-Aseguinolaza, G. (2021). Novel Vectors and Approaches for Gene Therapy in Liver Diseases. JHEP Rep. *3*, 100300. https://doi.org/10.1016/j.jhepr.2021.100300.

53. Blasco, H., Lalmanach, G., Godat, E., Maurel, M.C., Canepa, S., Belghazi, M., Paintaud, G., Degenne, D., Chatelut, E., Cartron, G., and Le Guellec, C. (2007). Evaluation of a peptide ELISA for the detection of rituximab in serum. J. Immunol. Methods *325*, 127–139. https://doi.org/10.1016/j.jim.2007.06.011.

54. Xu, R., Janson, C.G., Mastakov, M., Lawlor, P., Young, D., Mouravlev, A., Fitzsimons, H., Choi, K.L., Ma, H., Dragunow, M., et al. (2001). Quantitative comparison of expression with adeno-associated virus (AAV-2) brain-specific gene cassettes. Gene Ther. *8*, 1323–1332. https://doi.org/10.1038/sj.gt.3301529.

55. Kuck, D., Kern, A., and Kleinschmidt, J.A. (2007). Development of AAV serotype-specific ELISAs using novel monoclonal antibodies. J. Virol. Methods *140*, 17–24. https://doi.org/10.1016/j.jviromet.2006.10.005.

56. Cecchini, S., Negrete, A., and Kotin, R.M. (2008). Toward exascale production of recombinant adeno-associated virus for gene transfer applications. Gene Ther. *15*, 823–830. https://doi.org/10.1038/gt.2008.61.

57. Bepperling, A., and Best, J. (2023). Comparison of three AUC techniques for the determination of the loading status and capsid titer of AAVs. Eur. Biophys. J. *52*, 401–413. https://doi.org/10.1007/s00249-023-01661-0.

58. Marino, M., Zhou, L., Rincon, M.Y., Callaerts-Vegh, Z., Verhaert, J., Wahis, J., Creemers, E., Yshii, L., Wierda, K., Saito, T., et al. (2022). AAV-mediated delivery of an anti-BACE1 VHH alleviates pathology in an Alzheimer's disease model. EMBO Mol. Med. *14*, e09824. https://doi.org/10.15252/emmm.201809824.

59. Fischer, M.D., McClements, M.E., Martinez-Fernandez de la Camara, C., Bellingrath, J.S., Dauletbekov, D., Ramsden, S.C., Hickey, D.G., Barnard, A.R., and MacLaren, R.E. (2017). Codon-Optimized RPGR Improves Stability and Efficacy of AAV8 Gene Therapy in Two Mouse Models of X-Linked Retinitis Pigmentosa. Mol. Ther. *25*, 1854–1865. https://doi.org/10.1016/j.ymthe.2017.05.005.

60. Miller, N.L., Raman, R., Clark, T., and Sasisekharan, R. (2022). Complexity of Viral Epitope Surfaces as Evasive Targets for Vaccines and Therapeutic Antibodies. Front. Immunol. *13*, 904609–904615. https://doi.org/10.3389/fimmu.2022.904609.

61. Gurda, B.L., Raupp, C., Popa-Wagner, R., Naumer, M., Olson, N.H., Ng, R., McKenna, R., Baker, T.S., Kleinschmidt, J.A., and Agbandje-McKenna, M. (2012). Mapping a Neutralizing Epitope onto the Capsid of Adeno-Associated Virus Serotype 8. J. Virol. *86*, 7739–7751. https://doi.org/10.1128/jvi.00218-12.

62. Asaadi, Y., Jouneghani, F.F., Janani, S., and Rahbarizadeh, F. (2021). A comprehensive comparison between camelid nanobodies and single chain variable fragments. Biomark. Res. *9*, 87. https://doi.org/10.1186/s40364-021-00332-6.

63. Maruno, T., Ishii, K., Torisu, T., and Uchiyama, S. (2023). Size Distribution Analysis of the Adeno-Associated Virus Vector by the c(s) Analysis of Band Sedimentation Analytical Ultracentrifugation with Multiwavelength Detection. J. Pharmaceut. Sci. *112*, 937–946. https://doi.org/10.1016/j.xphs.2022.10.023.

64. Barnes, L.F., Draper, B.E., Chen, Y.T., Powers, T.W., and Jarrold, M.F. (2021). Quantitative analysis of genome packaging in recombinant AAV vectors by charge detection mass spectrometry. Mol. Ther. Methods Clin. Dev. *23*, 87–97. https://doi.org/10.1016/j.omtm.2021.08.002.

65. Li, Y., Struwe, W.B., and Kukura, P. (2020). Single molecule mass photometry of nucleic acids. Nucleic Acids Res. *48*, E97. https://doi.org/10.1093/nar/gkaa632.

66. Maruno, T., Usami, K., Ishii, K., Torisu, T., and Uchiyama, S. (2021). Comprehensive Size Distribution and Composition Analysis of Adeno-Associated Virus Vector by Multiwavelength Sedimentation Velocity Analytical Ultracentrifugation. J. Pharmaceut. Sci. *110*, 3375–3384. https://doi.org/10.1016/j.xphs.2021.06.031.

67. Gagnon, P., Goricar, B., Mencin, N., Zvanut, T., Peljhan, S., Leskovec, M., and Strancar, A. (2021). Multiple-monitor HPLC assays for rapid process development, in-process monitoring, and validation of AAV production and purification. Pharmaceutics *13*, 113–114. https://doi.org/10.3390/pharmaceutics13010113.

68. Khatwani, S.L., Pavlova, A., and Pirot, Z. (2021). Anion-exchange HPLC assay for separation and quantification of empty and full capsids in multiple adeno-associated virus serotypes. Mol. Ther. Methods Clin. Dev. *21*, 548–558. https://doi.org/10.1016/j.omtm.2021.04.003.

69. Cui, M., Lu, Y., Tang, C., Zhang, R., Wang, J., Si, Y., Cheng, S., and Ding, W. (2019). A generic method for fast and sensitive detection of adeno-associated viruses using modified AAV receptor recombinant proteins. Molecules *24*, 3973. https://doi.org/10.3390/molecules24213973.

70. Duong, T., McAllister, J., Eldahan, K., Wang, J., Onishi, E., Shen, K., Schrock, R., Gu, B., and Wang, P. (2023). Improvement of Precision in Recombinant Adeno-Associated Virus Infectious Titer Assay with Droplet Digital PCR as an Endpoint Measurement. Hum. Gene Ther. *34*, 742–757. https://doi.org/10.1089/hum.2023.014.

71. Leatham, B., Mcnall, K., Subramanian, H.K., Jacky, L., Alvarado, J., Yurk, D., Wang, M., Green, D.C., Tsongalis, G.J., Rajagopal, A., et al. (2023). A rapid, multiplex digital PCR assay for EGFR, KRAS, BRAF, ERBB2 variants and ALK, RET, ROS1, NTRK1 gene fusions in non-small cell lung cancer. Preprint at bioRxiv *34*, 742–757. https://doi.org/10.1101/2023.03.09.531949.

72. Xu, Y., Guo, P., Zhang, J., Chrzanowski, M., Chew, H., Firrman, J.A., Sang, N., Diao, Y., and Xiao, W. (2020). Effects of Thermally Induced Configuration Changes on rAAV Genome's Enzymatic Accessibility. Mol. Ther. Methods Clin. Dev. *18*, 328–334. https://doi.org/10.1016/j.omtm.2020.06.005.

73. Jenkins, R., Duggan, J.X., Aubry, A.F., Zeng, J., Lee, J.W., Cojocaru, L., Dufield, D., Garofolo, F., Kaur, S., Schultz, G.A., et al. (2015). Recommendations for Validation of LC-MS/MS Bioanalytical Methods for Protein Biotherapeutics. AAPS J. *17*, 1–16. https://doi.org/10.1208/s12248-014-9685-5.

74. Heckel, J., Martinez, A., Elger, C., Haindl, M., Leiss, M., Ruppert, R., Williams, C., Hubbuch, J., and Graf, T. (2023). Fast HPLC-based Affinity Method to Determine Capsid Titer and Full/Empty Ratio of Adeno-Associated Viral Vectors. Mol. Ther. Methods Clin. Dev. *31*, 101148. https://doi.org/10.1016/j.omtm.2023.101148.

75. Yang, Q.-E., Walton, R.W., Kudrolli, T., Denys, N., Lance, K., and Chang, A. (2022). Rapid Quality Control Assessment of Adeno-Associated Virus Vectors Via Stunner. GEN Biotechnology *1*, 300–310. https://doi.org/10.1089/genbio.2022.0007.

76. Srivastava, A., Mallela, K.M.G., Deorkar, N., and Brophy, G. (2021). Manufacturing Challenges and Rational Formulation Development for AAV Viral Vectors. J. Pharm. Sci. *110*, 2609–2624. https://doi.org/10.1016/j.xphs.2021.03.024.

77. Rayaprolu, V., Kruse, S., Kant, R., Venkatakrishnan, B., Movahed, N., Brooke, D., Lins, B., Bennett, A., Potter, T., McKenna, R., et al. (2013). Comparative Analysis of Adeno-Associated Virus Capsid Stability and Dynamics. J. Virol. *87*, 13150–13160. https://doi.org/10.1128/jvi.01415-13.

78. Wright, J.F. (2014). Product-related Impurities in Clinical-Grade Recombinant AAV Vectors: Characterization and Risk Assessment. Preprint at MDPI AG. Biomedicines *2*, 80–97. https://doi.org/10.3390/biomedicines2010080.

79. Dobnik, D., Kogovšek, P., Jakomin, T., Košir, N., Žnidarič, M.T., Leskovec, M., Kaminsky, S.M., Mostrom, J., Lee, H., and Ravnikar, M. (2019). Accurate quantification and characterization of adeno-associated viral vectors. Front. Microbiol. *10*, 1570. https://doi.org/10.3389/fmicb.2019.01570.

80. Shmidt, A.A., and Egorova, T.V. (2021). PCR-based analytical methods for quantification and quality control of recombinant adeno-associated viral vector preparations. Pharmaceuticals *15*, 23. https://doi.org/10.3390/ph15010023.

81. Gimpel, A.L., Katsikis, G., Sha, S., Maloney, A.J., Hong, M.S., Nguyen, T.N.T., Wolfrum, J., Springs, S.L., Sinskey, A.J., Manalis, S.R., et al. (2021). Analytical Methods for Process and Product Characterization of Recombinant Adeno-Associated Virus-Based Gene Therapies. Mol. Ther. Methods Clin. Dev. *20*, 740–754. https://doi.org/10.1016/j.omtm.2021.02.010.

82. Bennett, A., Patel, S., Mietzsch, M., Jose, A., Lins-Austin, B., Yu, J.C., Bothner, B., McKenna, R., and Agbandje-McKenna, M. (2017). Thermal Stability as a Determinant of AAV Serotype Identity. Mol. Ther. Methods Clin. Dev. *6*, 171–182. https://doi.org/10.1016/j.omtm.2017.07.003.

83. Kolbeck, P.J., Vanderlinden, W., Gemmecker, G., Gebhardt, C., Lehmann, M., Lak, A., Nicolaus, T., Cordes, T., and Lipfert, J. (2021). Molecular structure, DNA binding mode, photophysical properties and recommendations for use of SYBR Gold. Nucleic Acids Res. *49*, 5143–5158. https://doi.org/10.1093/nar/gkab265.

84. Tuma, R.S., Beaudet, M.P., Jin, X., Jones, L.J., Cheung, C.Y., Yue, S., and Singer, V.L. (1999). Characterization of SYBR gold nucleic acid gel stain: A dye optimized for use with 300-nm ultraviolet transilluminators. Anal. Biochem. *268*, 278–288. https://doi.org/10.1006/abio.1998.3067.

85. Schuck, P. (1998). Sedimentation analysis of noninteracting and self-associating solutes using numerical solutions to the Lamm equation. Biophys. J. *75*, 1503–1512. https://doi.org/10.1016/S0006-3495(98)74069-X.

86. Philo, J.S. (2023). SEDNTERP: a calculation and database utility to aid interpretation of analytical ultracentrifugation and light scattering data. Eur. Biophys. J. *52*, 233–266. https://doi.org/10.1007/s00249-023-01629-0.

87. Brautigam, C.A. (2015). Calculations and Publication-Quality Illustrations for Analytical Ultracentrifugation Data, 1st ed. (Elsevier Inc), pp. 109–133. https://doi.org/10.1016/bs.mie.2015.05.001.

OMTM, Volume *32*

Supplemental information

Quantification of full and empty particles of adeno-associated virus vectors via a novel dual fluorescence-linked immunosorbent assay

Sereirath Soth, Mikako Takakura, Masahiro Suekawa, Takayuki Onishi, Kiichi Hirohata, Tamami Hashimoto, Takahiro Maruno, Mitsuko Fukuhara, Yasuo Tsunaka, Tetsuo Torisu, and Susumu Uchiyama

Supplemental Figures

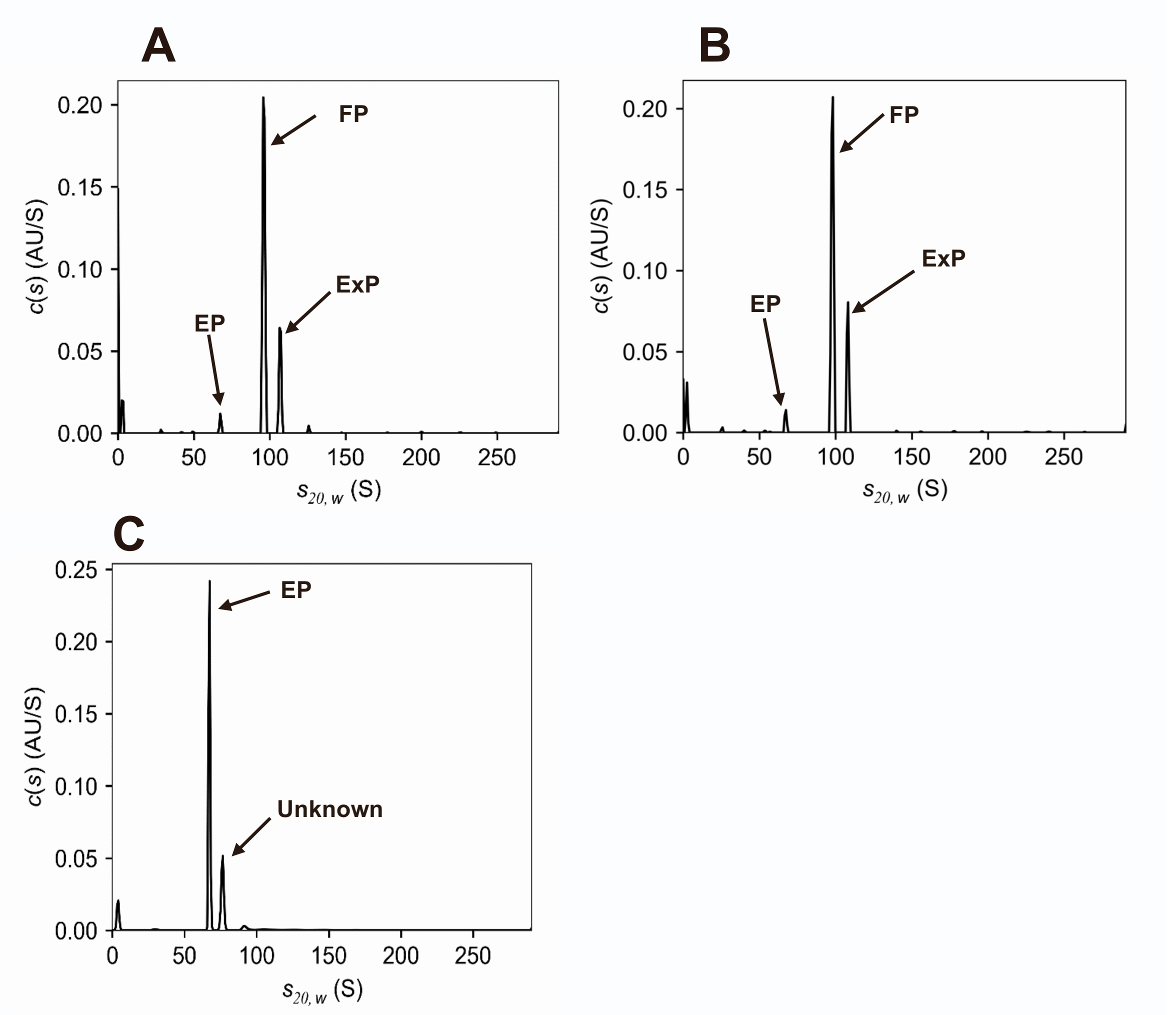

Figure S1. Representative sedimentation coefficient distributions in PBS/D_2O + 0.001% poloxamer-188 for AAV8-Lot1 to AAV8-Lot3 vector samples (see in Table S1).

(A) Sedimentation coefficient distribution of AAV8-Lot1 vector sample, which is used as the standard for dFLISA analysis.

(B) Sedimentation coefficient distribution of AAV8-Lot2 vector sample, which is used as sample for dFLISA analysis.

(C) Sedimentation coefficient distribution of AAV8-Lot3 vector sample, which is used as sample for dFLISA analysis.

The observed peaks are assigned as empty particle (EP), full particle (FP), or extra filled (ExP). [1,2]

It is important to note that the unknown peak was not counted as particle.

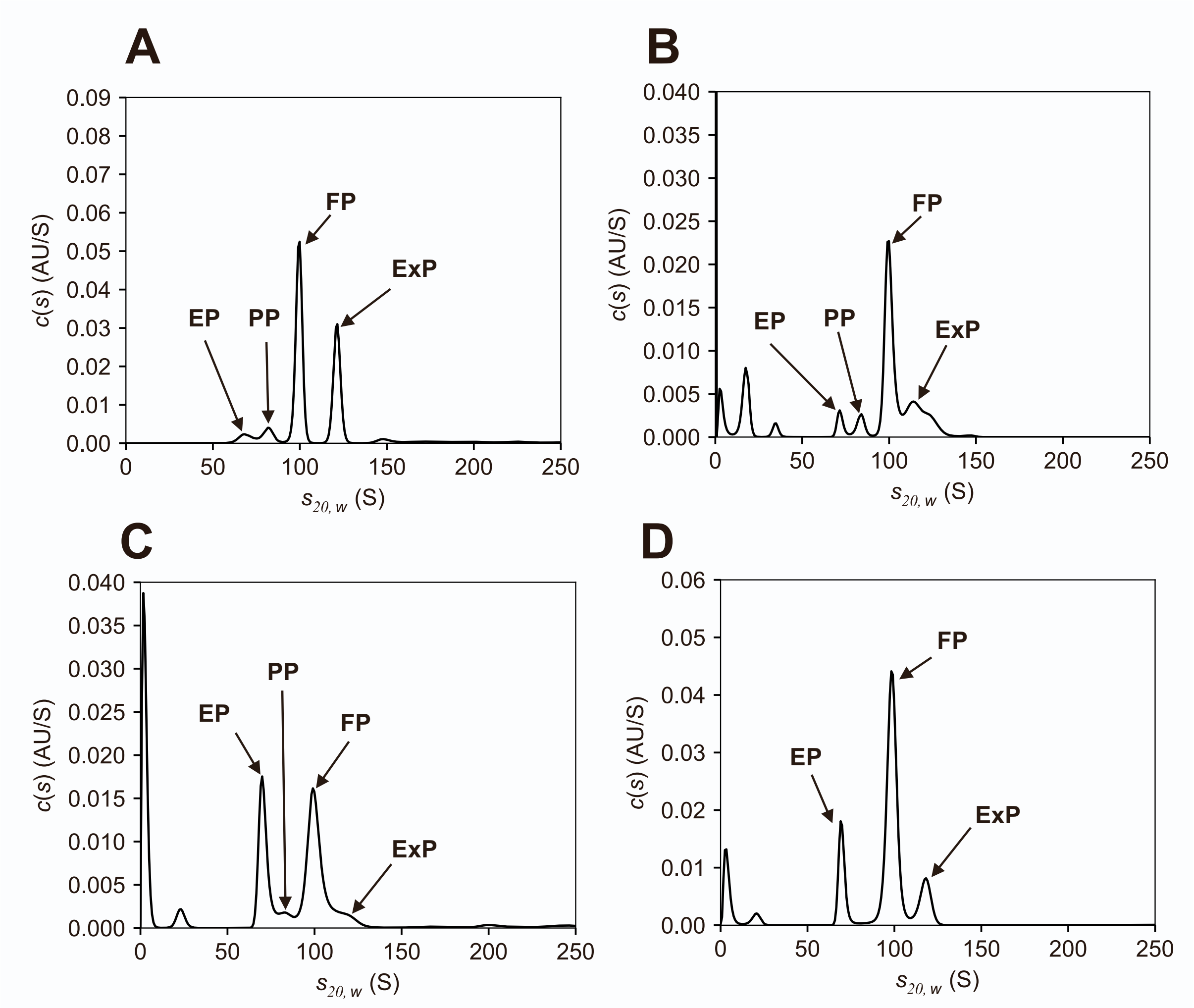

Figure S2. Representative sedimentation coefficient distributions in PBS/D_2O + 0.001% poloxamer-188 for AAV2-Lot3 to AAV2-Lot4 and AAV8-Lot5 to AAV8-Lot6 and vector samples (see in Table S2).

(A) Sedimentation coefficient distribution of AAV2-Lot3 vector sample, which is used as the Standard for dFLISA analysis.

(B) Sedimentation coefficient distribution of AAV2-Lot4 vector sample, which is used as the sample for dFLISA analysis.

(C) Sedimentation coefficient distribution of AAV8-Lot5 vector sample, which is used as the standard for dFLISA analysis.

(D) Sedimentation coefficient distribution of AAV8-Lot6 vector sample, which is used as the sample for dFLISA analysis.

The observed peaks are assigned as empty particle (EP), full particle (FP), extra filled particle (ExP) or partial particle (PP).

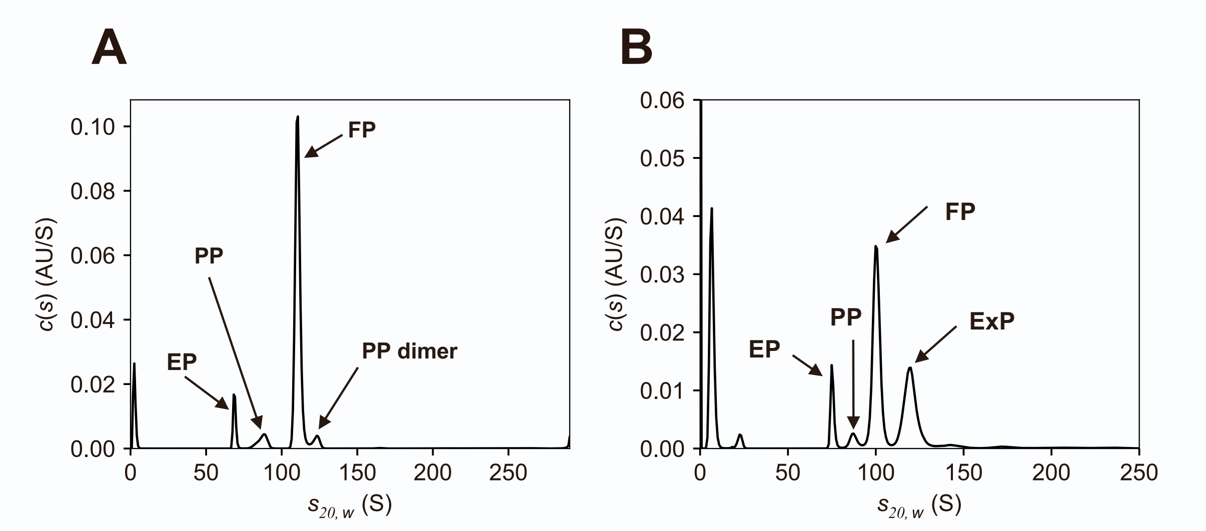

Figure S3. Representative sedimentation coefficient distributions in PBS/D_2O + 0.001% poloxamer-188 for AAV2-Lot1 to AAV2- Lot2 vector samples (see in Table S2).

(A) Sedimentation coefficient distribution of AAV2-Lot1 vector sample, which is used as the standard for dFLISA analysis. The observed peaks were identified as EP, PP, FP and PP dinner. PP is higher than LOQ, and the PP dimer is lower than LOQ of BS-AUC.

(B) Sedimentation coefficient distribution of AAV2-Lot2 vector sample, which is used as standard for dFLISA analysis. The observed peaks are assigned as empty particle (EP), full particle (FP); partial particle (PP) as shown in the figures.

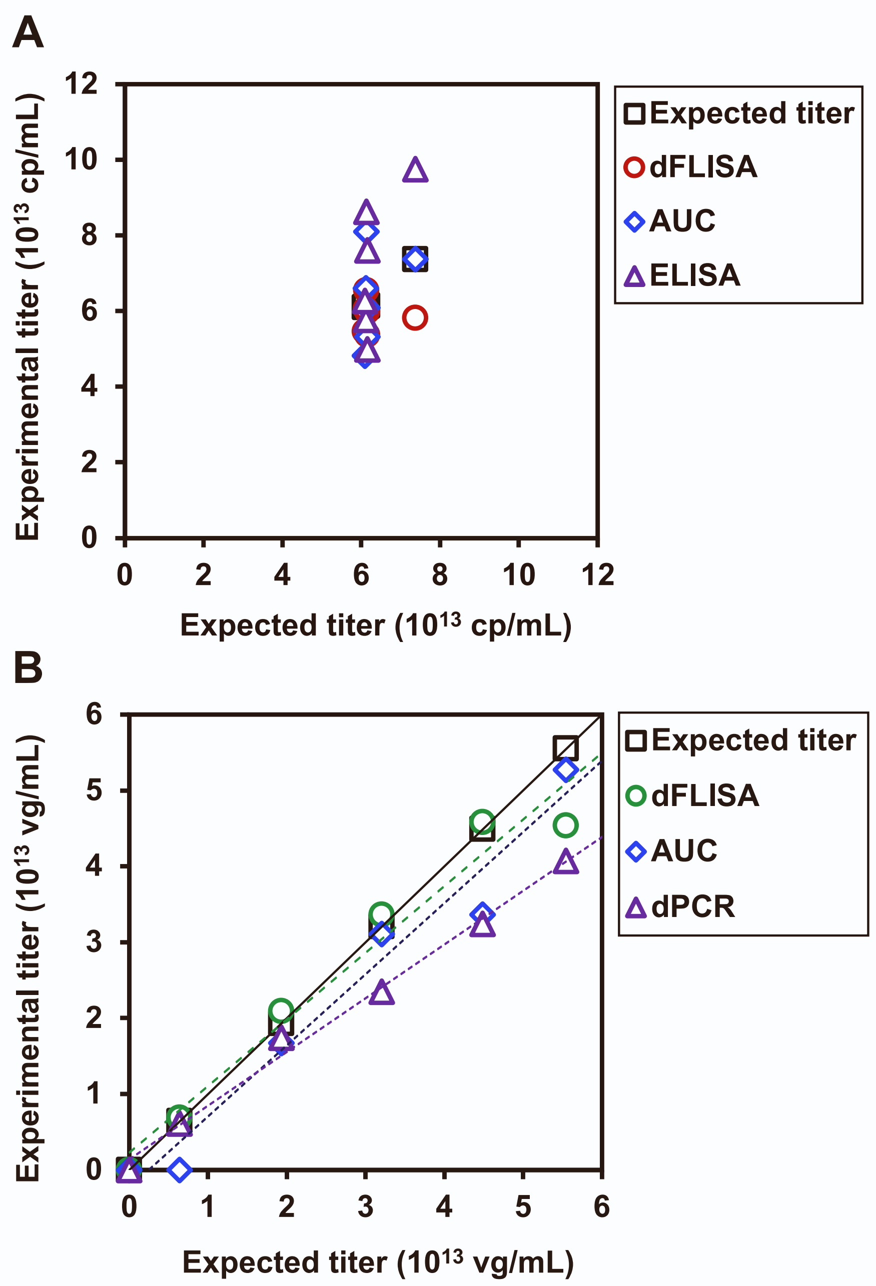

Figure S4. Linear correlation of total capsid titers (cp/mL) and genomic titer (vg/mL) of six mixed spike samples.

(A) Comparison of capsid titers (cp/mL) of six mixed spike samples analyzed by three techniques: dFLISA, BS-AUC, and ELISA. The capsid titer was adjusted to 6.16×10^{13} cp/mL for the mixed samples and to 6.09×10^{13} cp/mL for 0% FPs sample. The expected capsid titers (black square) were plotted on the horizontal axis, and the corresponding experimental capsid titer obtained by dFLISA

(green circle), BS-AUC (light blue rhombus) and ELISA (purple triangle) were plotted on the vertical axis.

(B) Comparison of genomic titers (vg/mL) of six mixed spike samples analyzed by three techniques: dFLISA, BS-AUC, and dPCR. The linear correlation of the expected genomic titers was plotted on the horizontal axis and the corresponding experimental genomic titer obtained by dFLISA (green circle), BS-AUC (light blue rhombus) and dPCR (purple triangle) were plotted on the vertical axis.

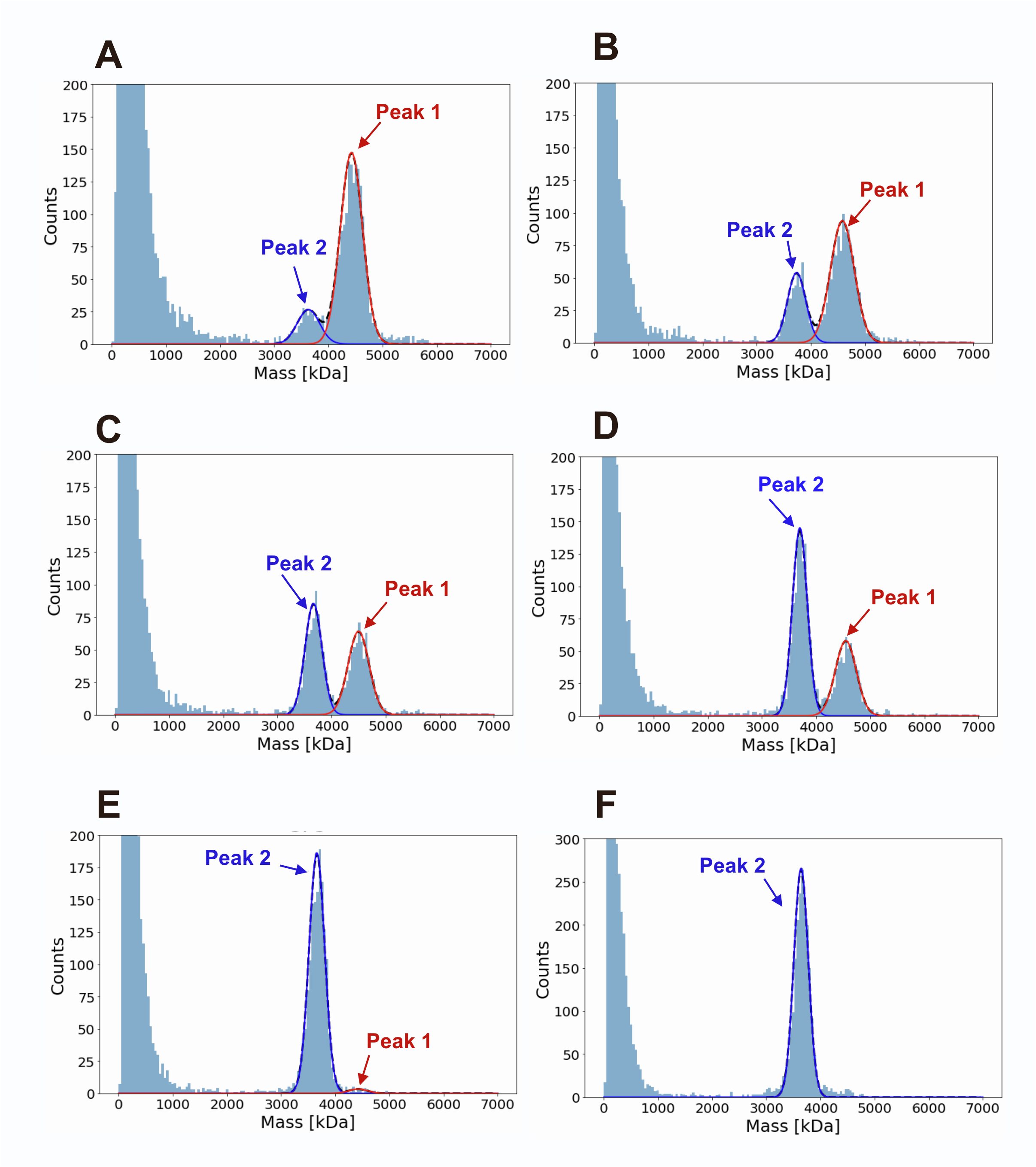

Figure S5. Histogram from MP analysis[3,5] of ssDNA packaged AAV8 capsids obtained from six mixed spike samples (90.1%, 73.1%, 52.3%, 31.5%, 10.5%, and 0% FPs).

(A) Constructed mass histograms of 90.1 % full capsid.

(B) Constructed mass histograms of 73.1 % full capsid.

(C) Constructed mass histograms of 52.3 % full capsid.

(D) Constructed mass histograms of 31.5 % full capsid.

(E) Constructed mass histograms of 10.5 % full capsid.

(F) Constructed mass histogram of 0 % full capsid.

Observed Peak1 (red) with mass corresponding to FP, while the observed Peak 2 (blue) with mass corresponding to EP. For each AAV8 sample, a single representative mass histogram is displayed. Gaussian distribution fit was applied to the histogram peaks. From these Gaussian fits, the percentage of full and empty AAV8 capsids were extracted.

EP, empty particle; FP; full particle; MP, mass photometry.

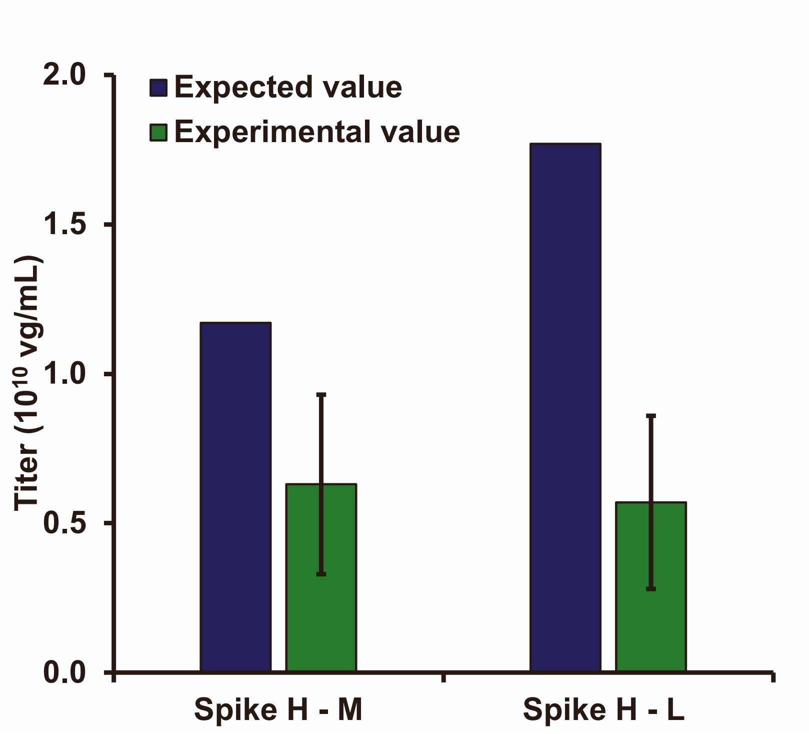

Figure S6. Spike-recovery test of dPCR.

Genomic titer quantification of crude samples by dPCR [7–9] were evaluated by spike-recovery test. The spike-recovery test was conducted as described in the method section: Quantification of crude sample by dFLISA and other method.

The different dilution factors for spike recovery were assessed by comparing the experimental values, as determined by the dPCR (dark green), with the expected values obtained by dFLISA (dark blue). The standard deviation (SD) of each parameter was obtained from the triplicated experiments.

H, high concentration spike; M, middle concentration spike; L, low concentration spike

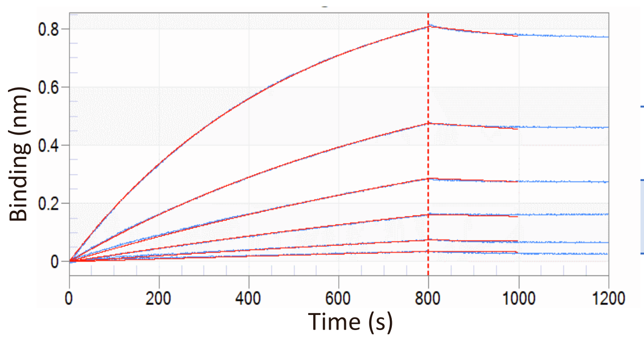

K_D (M)	k_{on} (1/Ms)	k_{off} (1/s)
2.73×10^{-11}	4.69×10^{6}	1.28×10^{-4}

Figure S7. Binding kinetics and affinity of AAV2 with anti-AAV VHH antibody measured by BLI

Biolayer Interferometry (BLI) measurements[4,6] were performed on Octet HTX system (Sartorius, Goettingen, Germany). Octet SAX biosensors (Sartorius) were hydrated by immersion in PBS for at least 10 min prior to use. Biotinylated anti-AAV VHH antibody (CaptureSelect™ Biotin Anti-AAVX Conjugate, Thermo Fisher Scientific) was diluted to 0.01 µg/mL and immobilized on the SAX biosensors. AAV2 samples were diluted in a 2-fold dilution series from 2.50×10^{11} vg/mL to 1.56×10^{10} vg/mL with Octet sample diluent (Sartorius). After the baseline measurement using Octet sample diluent, association and dissociation of AAV2 with the anti-AAV VHH antibody were measured at 30°C in an Octet 384-well tilted-bottom microplate (Sartorius) while shaking at 1000 rpm. Octet Analysis Studio software (ver. 12.2) was used for data analysis. The interaction between anti-AAV VHH antibody and AAV2 was analyzed by 1:1 fitting.

According to the manufacture's website of the 96-well plate, the maximum coating amount is 650 ng/cm^2. The bottom area of 96-well plates is approximately 0.33 cm^2, and the volume of AAV solution was 100 microliters. Thus, the coated concentration of VHH, whose molecular weight is 14 kDa, would be 150 nM. Using these values and the result of BLI, we calculated the binding efficiency of AAV based on the following formula:[10]

$$[\text{Complex}] = \frac{(K_D + [AAV] + [VHH]) - \sqrt{(K_D + [AAV] + [VHH])^2 - 4[AAV][VHH]}}{2}$$

$$\text{Binding efficiency} = \frac{[Complex]}{[AAV]} \times 100$$

The binding efficiency of VHH antibody and AAV2 was >99% over the entire range of the standard curve. Although there is no information about the affinity of VHH for AAV8, the binding efficiency would be >98% even if K_D value of VHH for AAV8 is 100 times larger than that for AAV2.

Supplemental Tables

Table S1. Summary of in-house AAV8 vectors used in this study, as determined by BS-AUC

Sample name	Serotype	Promoter	GOI	Number of nucleic acids	FP (10^{13} vg/mL)	EP (10^{13} cp/mL)	ExP (10^{13} vg/mL)	PP (10^{13} vg/mL)	FP (%)	EP (%)	ExP (%)	PP (%)	FP+ExP (%)	FP+ExP (10^{13} vg/mL)	Total FP+ExP+EP (10^{13} cp/mL)	Sample preparation
AAV8-Lot1	8	CMV	EGFP	2521	1.09	0.11	0.24	<LOQ	76.10	8.23	15.67	na	91.8	1.31	1.43	Standard
AAV8-Lot2	8	HCRhAAT	FIX	2712	4.75	0.61	0.79	<LOQ	77.15	9.90	12.95	na	90.1	5.55	6.16	Sample
AAV8-Lot3	8	na	na	na	na	7.37	na	na	na	100	na	na	na	na	7.37	Sample

BS-AUC, band sedimentation analytical ultracentrifugation.
GOI, gene of interest; CMV, cytomegalovirus promoter; HCRhAAT, hepatic control region and human a1 antitrypsin promoter; EGFP, enhanced green fluorescent protein; FIX, factor IX.
cp, capsid particle; vg, viral genome; EP, empty particle; ExP, extra filled particle; FP, full particle; PP, partial particle; LOQ, Limit of quantification; na, not applicable.

Table S2. Summary of AAVs vectors purchased from VectorBuilder in this study, as determined by BS-AUC

Sample name	Serotype	Promoter	GOI	Number of nucleic acids	FP (10^{12} vg/mL)	EP (10^{12} cp/mL)	ExP (10^{12} vg/mL)	PP (10^{12} vg/mL)	FP (%)	EP (%)	ExP (%)	PP (%)	FP+ExP (%)	FP+ExP (10^{12} vg/mL)	Total FP+ExP+EP (10^{12} cp/mL)	Sample preparation
AAV2-Lot1	2	CMV	EGFP	2521	1.87	0.27	0.94	<LOQ	60.5	8.9	30.9	na	91.1	2.82	3.09	Sample
AAV2-Lot2	2	CMV	EGFP	3681	3.33	0.40	<LOQ	<LOQ	89.1	10.9	na	na	89.1	3.33	3.73	Sample
AAV2-Lot3	2	CMV	EGFP	2521	10.3	1.21	6.12	<LOQ	58.5	6.9	34.6	na	93.1	16.5	17.7	Standard
AAV2-Lot4	2	CMV	EGFP	2521	6.12	0.75	2.76	<LOQ	63.6	7.8	28.6	na	92.2	8.88	9.63	Sample
AAV8-Lot5	8	CMV	EGFP	2521	7.43	2.34	<LOQ	<LOQ	72.2	22.7	na	na	77.3	7.95	10.3	Standard
AAV8-Lot6	8	CMV	EGFP	2521	7.83	6.60	<LOQ	<LOQ	53.6	45.1	na	na	54.9	8.02	14.6	Sample

BS-AUC, band sedimentation analytical ultracentrifugation.
GOI, gene of interest.
CMV, cytomegalovirus promoter.
EGFP, enhanced green fluorescent protein.
cp, capsid particle; vg, viral genome EP, empty particle; ExP, extra filled particle; FP, full particle; LOQ, Limit of quantification; PP, partial particle; na, not applicable.

Table S3. Precision and accuracy of the dFLISA of capsid titer (cp/mL)

Sample	Expected value	Experimental value				SD (10^{10} cp/mL)	CV (%)	Accuracy (%)
	Concentration (10^{10} cp/mL)	Concentration (10^{10} cp/mL)						
	Concentration	Day 1	Day 2	Day 3	Average titer			
1	15.4	15.7	15.9	16.1	15.9	0.21	1.3	102.8
2	7.70	6.98	6.48	6.45	6.64	0.29	4.4	87.6
3	3.85	3.50	3.31	3.23	3.35	0.14	4.2	87.6
4	1.93	1.81	1.53	1.50	1.61	0.17	10.6	84.1
5	0.96	0.84	0.67	0.76	0.76	0.08	10.8	76.0
6	0.48	0.29	0.39	0.35	0.34	0.04	14.4	74.3
7	0.24	0	0	0.16	0	0	0	0

Results represent the mean values from 3-day experiments, in which each sample was analyzed in duplicate wells.
The samples were initially diluted 400-fold, followed by a 2-fold serial dilution.
CV, coefficient of variation; cp, capsid particle; SD, standard deviation.

Table S4. Precision and accuracy of the dFLISA of genomic titer (vg/mL)

Sample	Expected value	Experimental value				Full capsid ratio (%)	SD (10^{10} vg/mL)	CV (%)	Accuracy (%)
	Concentration (10^{10} vg/mL)	Concentration (10^{10} vg/mL)							
	Concentration	Day 1	Day 2	Day 3	Average titer				
1	13.9	13.7	14.0	13.3	13.7	85.8	0.31	2.3	98.4
2	6.94	6.60	6.58	6.37	6.52	98.2	0.12	1.9	93.9
3	3.47	3.27	3.10	2.89	3.09	92.2	0.18	6.1	89.0
4	1.73	1.80	1.47	1.14	1.47	91.3	0.33	22.6	84.7
5	0.86	0.69	0.35	0.31	0.45	60.1	0.21	46.1	52.7
6	0.43	0	0	0	0	0	0	0	0
7	0.21	0	0.54	0	0	0	0	0	0

Results represent the mean values from 3-day experiments, in which each sample was analyzed in duplicate wells.
The samples were initially diluted 400-fold, followed by a 2-fold serial dilution.
CV, coefficient of variation; SD, standard deviation; vg, viral genome.

Table S5. Determination LOQ of dFLISA for capsid titer detection

Blank Intensity (10^4)					Concentration calculated from blank intensity + 10 SD (10^{10} cp/mL)
Day 1	Day 2	Day 3	Average intensity	SD	
1.65	2.91	2.05	2.20	0.29	0.60

The limit of quantification (LOQ) of capsid quantification are estimated from fluorescence intensities of blank.
Results represent the mean results of 3-day experiments in which each sample was analyzed in duplicate wells.
cp, capsid particle; SD, standard deviation.

Table S6. Determination LOQ of dFLISA for genomic titer detection

Blank Intensity (10^4)					Concentration calculated from blank intensity + 10 SD (10^{10} vg/mL)
Day 1	Day 2	Day 3	Average intensity	SD	
10.0	9.10	9.20	9.56	1.27	1.70

The limit of quantification (LOQ) of genome quantification are estimated from fluorescence intensities of blank.
Results represent the mean results of 3-day experiments in which each sample was analyzed in duplicate wells.
SD, standard deviation.
vg, viral genome.

Table S7. Comparison of capsid titers (cp/mL) of six mixed spike samples using orthogonal techniques: dFLISA, BS-AUC, and ELISA

Sample	Expected value	Experimental value		
	AUC (10^{13} cp/mL)	dFLISA (10^{13} cp/mL)	AUC (10^{13} cp/mL)	ELISA (10^{13} cp/mL)
90.1% full	6.16	6.36	6.09	7.60
73.1% full	6.15	7.10	5.32	4.99
52.3% full	6.13	7.77	8.11	8.63
31.5% full	6.12	7.17	6.60	5.77
10.5% full	6.10	6.46	4.82	6.26
0% full	7.37	5.71	7.37	9.75

AUC, analytical ultracentrifugation

dFLISA, dual fluorescence-linked immunosorbent assay.

ELISA, enzyme-linked immunosorbent assay.

cp, capsid particle.

Table S8. Comparison of genomic titers (vg/mL) of six mixed spike samples using orthogonal techniques: dFLISA, BS-AUC, and dPCR

Sample	Expected value	Experimental value		
	AUC (10^{13} vg/mL)	dFLISA (10^{13} vg/mL)	AUC (10^{13} vg/mL)	dPCR (10^{13} vg/mL)
90.1% full	5.55	5.46	5.27	4.07
73.1% full	4.49	5.51	3.36	3.24
52.3% full	3.21	4.04	3.11	2.35
31.5% full	1.93	2.51	1.67	1.74
10.5% full	0.64	8.39	nd[a]	0.61
0% full	0	0	0	0

AUC, analytical ultracentrifugation

dFLISA, dual fluorescence-linked immunosorbent assay.

dPCR, digital chain polymerase reaction.

[a]nd, not detected; vg, viral genome.

Table S9. Comparison of fluorescence intensity of AAV2 with different genome lengths by dFLISA

Genomic titer		Fluorescence intensity[c]			
(10^{10} vg/mL)	scDNA[a] (10^5)	ssDNA[b] (10^5)	Ratio (scDNA/ssDNA)	Average value	SD
1.41	11.11	5.93	1.87		
0.70	5.95	3.22	1.85	1.86	0.015
0.35	3.49	1.89	1.85		

[a]scDNA (3681 base), self-complementary DNA.

[b]ssDNA (2521 base), single-stranded DNA.

[c]Calculated from standard curve of AAV vectors with scDNA and ssDNA.

SD, standard deviation.

References

1. Maruno, T., Ishii, K., Torisu, T., and Uchiyama, S. (2023). Size Distribution Analysis of the Adeno-Associated Virus Vector by the c(s) Analysis of Band Sedimentation Analytical Ultracentrifugation with Multiwavelength Detection. J Pharm Sci *112*, 937–946. https://doi.org/10.1016/j.xphs.2022.10.023.
2. Hirohata, K., Yamaguchi, Y., Maruno, T., Shibuya, R., Torisu, T., Onishi, T., Chono, H., Mineno, J., Yuzhe, Y., Higashiyama, K., et al. (2024). Applications and Limitations of Equilibrium Density Gradient Analytical Ultracentrifugation for the Quantitative Characterization of Adeno-Associated Virus Vectors. Anal Chem *96*, 642–651. https://doi.org/10.1021/acs.analchem.3c01955.
3. Hiemenz, C., Baumeister, N., Helbig, C., Hawe, A., Babutzka, S., Michalakis, S., Friess, W., and Menzen, T. (2023). Genome length determination in adeno-associated virus vectors with mass photometry. Mol Ther Methods Clin Dev *31*. https://doi.org/10.1016/j.omtm.2023.101162.
4. Meierrieks, F., Kour, A., Pätz, M., Pflanz, K., Wolff, M.W., and Pickl, A. (2023). Unveiling the secrets of adeno-associated virus: novel high-throughput approaches for the quantification of multiple serotypes. Mol Ther Methods Clin Dev *31*. https://doi.org/10.1016/j.omtm.2023.101118.
5. Wagner, C., Fuchsberger, F.F., Innthaler, B., Lemmerer, M., and Birner-Gruenberger, R. (2023). Quantification of Empty, Partially Filled and Full Adeno-Associated Virus Vectors Using Mass Photometry. Int J Mol Sci *24*. https://doi.org/10.3390/ijms241311033.
6. Fu, Y., Choudhary, D., Liu, N., Moon, Y., Abdubek, P., Sweezy, L., Rosconi, M., Palackal, N., and Pyles, E. (2023). Comprehensive biophysical characterization of AAV-AAVR interaction uncovers serotype- and pH-dependent interaction. J Pharm Biomed Anal *234*. https://doi.org/10.1016/j.jpba.2023.115562.
7. Shmidt, A.A., and Egorova, T. V. (2022). PCR-based analytical methods for quantification and quality control of recombinant adeno-associated viral vector preparations. Preprint at MDPI, https://doi.org/10.3390/ph15010023 https://doi.org/10.3390/ph15010023.
8. Dobnik, D., Kogovšek, P., Jakomin, T., Košir, N., Žnidarič, M.T., Leskovec, M., Kaminsky, S.M., Mostrom, J., Lee, H., and Ravnikar, M. (2019). Accurate quantification and characterization of adeno-associated viral vectors. Front Microbiol *10*. https://doi.org/10.3389/fmicb.2019.01570.
9. Kojabad, A.A., Farzanehpour, M., Galeh, H.E.G., Dorostkar, R., Jafarpour, A., Bolandian, M., and Nodooshan, M.M. (2021). Droplet digital PCR of viral DNA/RNA, current progress, challenges, and future perspectives. Preprint at John Wiley and Sons Inc, https://doi.org/10.1002/jmv.26846 https://doi.org/10.1002/jmv.26846.
10. Marsh Editor, J.A. Protein Complex Assembly Methods and Protocols Methods in Molecular Biology 1764.
